# Preliminary Insight of Pyrrolylated-Chalcones as New Anti-Methicillin-Resistant *Staphylococcus aureus* (Anti-MRSA) Agents

**DOI:** 10.3390/molecules26175314

**Published:** 2021-09-01

**Authors:** Mohanapriya Gunasekharan, Tae-Ik Choi, Yaya Rukayadi, Muhammad Alif Mohammad Latif, Thiruventhan Karunakaran, Siti Munirah Mohd Faudzi, Cheol-Hee Kim

**Affiliations:** 1 Department of Chemistry, Faculty of Science, Universiti Putra Malaysia, Serdang 43400, Selangor, Malaysia; mohanapriyagunasekharan@gmail.com; 2 Department of Biology, Chungnam National University, 99 Daehak-ro, Yuseong-gu, Daejeon 34134, Korea; c860523@naver.com; 3 Natural Medicines and Product Research Laboratory, Institute of Bioscience, Universiti Putra Malaysia, Serdang 43400, Selangor, Malaysia; yaya_rukayadi@upm.edu.my; 4 Department of Food Science, Faculty of Food Science and Technology, Universiti Putra Malaysia, Serdang 43400, Selangor, Malaysia; 5 Centre of Foundation Studies for Agricultural Science, Universiti Putra Malaysia, Serdang 43400, Selangor, Malaysia; aliflatif@upm.edu.my; 6 Centre for Drug Research, Universiti Sains Malaysia, Gelugor 11800, Pulau Pinang, Malaysia; thiruventhan@usm.my

**Keywords:** synthesis, pyrrolylated-chalcones, anti-MRSA, molecular docking, zebrafish toxicity

## Abstract

Bacterial infections are regarded as one of the leading causes of fatal morbidity and death in patients infected with diseases. The ability of microorganisms, particularly methicillin-resistant *Staphylococcus aureus* (MRSA), to develop resistance to current drugs has evoked the need for a continuous search for new drugs with better efficacies. Hence, a series of non-PAINS associated pyrrolylated-chalcones (**1**–**15**) were synthesized and evaluated for their potency against MRSA. The hydroxyl-containing compounds (**8**, **9**, and **10**) showed the most significant anti-MRSA efficiency, with the MIC and MBC values ranging from 0.08 to 0.70 mg/mL and 0.16 to 1.88 mg/mL, respectively. The time-kill curve and SEM analyses exhibited bacterial cell death within four hours after exposure to **9**, suggesting its bactericidal properties. Furthermore, the docking simulation between **9** and penicillin-binding protein 2a (PBP2a, PDB ID: 6Q9N) suggests a relatively similar bonding interaction to the standard drug with a binding affinity score of −7.0 kcal/mol. Moreover, the zebrafish model showed no toxic effects in the normal embryonic development, blood vessel formation, and apoptosis when exposed to up to 40 µM of compound **9**. The overall results suggest that the pyrrolylated-chalcones may be considered as a potential inhibitor in the design of new anti-MRSA agents.

## 1. Introduction

Bacterial infections are one of the significant threats to global health, mainly owing to the resistance of microorganisms towards antibiotics [1]. According to the Centre for Disease Control and Prevention (CDC) in the year 2017, more than 2 million diseases and 23,000 deaths occur annually due to antibiotic resistance in the United States. In the past two decades, the development of new antimicrobials for the treatment of Gram-positive bacterial infections has become a necessity [2]. *Staphylococcus aureus* is a Gram-positive bacterium that is found on the human skin and can invade the bone joints, respiratory system, and endovascular tissues through the bloodstream [3]. Patients with chronic diseases, i.e., adolescents, elder people, and patients undergoing treatment have a higher tendency to be infected by methicillin-resistant *S. aureus* (MRSA). In 1950, methicillin was found to be effective against *S. aureus*-related infections [4]. Unfortunately, by 1961, *S. aureus* was found to be resistant to methicillin. Since then, the number of hospital-acquired and community-acquired MRSA infections and deaths have increased to a number almost similar to that of the number of AIDS and tuberculosis deaths [5].

Although commercially designed antibiotics that treat bacterial infection-related diseases such as methicillin and vancomycin are widely available, these antibiotics however are gradually losing their efficiency due to the constantly evolving bacterial resistance towards antibiotics. Therefore, the need for developing new antibacterial agents that are effective against new strains of microorganisms has drastically increased worldwide.

Chalcones (1,3-diaryl-2-propenone) are acyclic compounds containing an α, β-unsaturated carbonyl group appended by two aromatic rings and are commonly synthesized via acid- or base-catalyzed Aldol or Claisen–Schmidt condensation reactions in the laboratory [6]. Chalcones and its derivatives have displayed a wide range of biological activities including antibacterial, antifungal, anti-inflammatory, antimalarial, anticancer, antiviral, anti-tubercular, anti-hyperglycemic [6], and antioxidant activities [7]. Some studies have also reported on chalcones with potential antimicrobial properties against fungi and bacteria, and even chalcones that are resistant to bacteria [8]. These activities have rendered chalcones as potential compounds for the development of new anti-MRSA agents.

**Figure 1:**
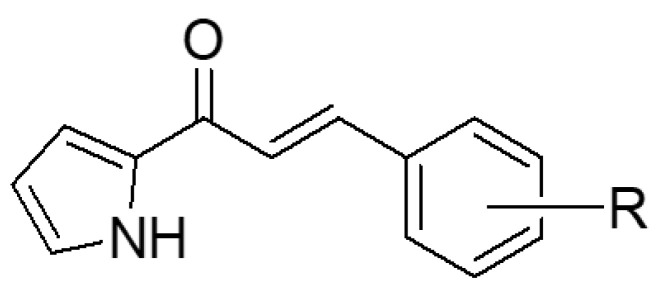
Molecular structure of chalcone (1,3-diaryl-2-propenone).

The recent discovery of chalcones that possess high anti-infective potentials and the therapeutic importance of nitrogen-containing marketed antibiotics, has led to the designation of a new chalconoid derivative, the pyrrolylated-chalcone (Figure 1), whereby the pyrrole moiety is incorporated into the chalcone scaffold. This fusion is hypothesized to further improve the binding interactions with the active site of MRSA, thus enhancing the targeted anti-MRSA activity.

As a continuation of our endeavors towards the exploration of the therapeutic potential of pyrrolylated-chalcones as new anti-MRSA hit-candidates, we herein evaluate the anti-MRSA properties of the pyrrolylated-chalcones (**1**–**15**) using in vitro disc diffusion, MIC, MBC, time-kill, and SEM analyses. The active molecules were then analyzed using molecular docking studies to further understand the binding mode and affinity of the compounds that exhibit the most potent and prominent MRSA inhibition. In addition, the most active compound **9**, was preliminarily profiled for in vivo toxicities using the zebrafish animal model.

## 2. Results and Discussion

### 2.1. Chemistry

**Scheme 1:**
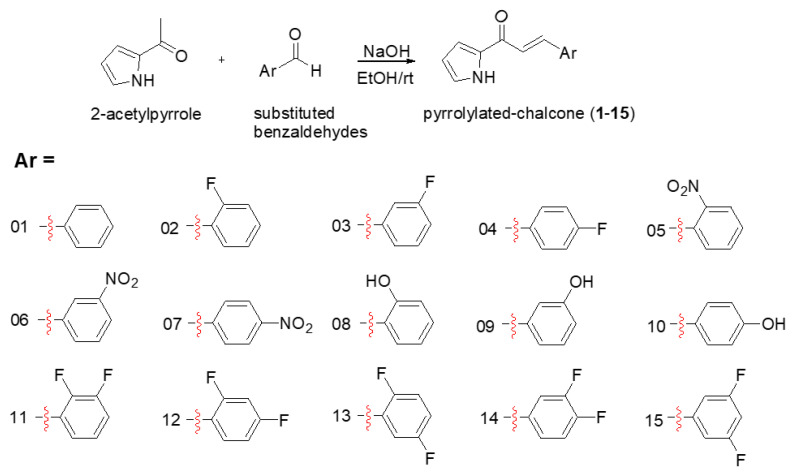
General synthetic preparation for pyrrolylated-chalcone analogues (**1**–**15**).

In this present study, a series of non-associated pan-assay interference compounds (PAINS) (see Supplementary Materials), the pyrrolylated-chalcones, including new analogues **5** and **11**–**15** were synthesized via Claisen–Schmidt condensation by reacting 2-acetylpyrrole with the appropriate fluoro-, nitro-, and hydroxyl-containing benzaldehydes in ethanolic sodium hydroxide for 24 h at room temperature (Scheme 1) [9]. All the synthesized compounds were purified by gravitational column chromatography and recrystallization techniques, followed by structural elucidation via spectroscopic analyses, including ^1^H-, ^13^C-, and ^19^F-NMR, Fourier transform infra-red (FT-IR), and direct infusion-mass spectrometry (DI-MS), as well as high-resolution mass spectrometry (HRMS) for new molecules. The spectroscopic data of the newly synthesized analogues (**5** and **11**–**15**), general physical properties (Table S1), as well as the predicted physicochemical and drug-likeness properties (Table S2) of the synthesized molecules (**1**–**15**) were appended in Supplementary Materials.

Compounds **1**–**15** exhibited the peaks of α- and β- methine moieties (δ_H_ 7.28–12.30 ppm and 7.67–12.30 ppm; δ_C_ 119.5–128.8 ppm and 131.1–141.1 ppm) with *J* values of 15.7–16.1 Hz, indicated the *trans* configuration for the conjugated olefinic system. Meanwhile, the pyrrole and phenolic proton(s) were spotted in the range of δ_H_ 6.17–7.64 ppm and 6.93–8.67 ppm.

The presence of electron-withdrawing groups (-F and -NO_2_), particularly the difluoro-substituted benzene gave a higher product yield, ranging from 65.4 to 90.2%. On the other hand, the hydroxylated compounds (electron-donating group) recorded lower yields (<12.9%). Taking this into account, we concluded that the shortening of the C=O bond due to the inductive effect exerted by the electron-withdrawing functionalities greatly improves the yield of the end products [10].

It is important to highlight that though many analogues of pyrrolylated-chalcones have been previously synthesized for various bioactivities [9,11,12,13,14], we would like to explore further the therapeutic uses of the synthesized chalcones particularly as anti-MRSA agents in this present study. Little has been reported on the in-depth anti-MRSA potential of pyrrolylated-chalcones thus far.

### 2.2. In Vitro Anti-MRSA Activity

Following that, the prospective antibacterial activity of the synthesized compounds against the standard ATCC 700699 and three clinical isolates (172918, 180620, and 180865) of *S. aureus* bacteria strains was first determined through the disc diffusion assay (DDA). Compounds with significant bacterial inhibition were then further tested via the minimum inhibitory concentration (MIC) and minimum bactericidal concentration (MBC) assays to confirm their bioactivity. The anti-MRSA efficiency results are displayed in Table 1.

#### 2.2.1. Disc Diffusion Assay

Based on the preliminary DDA screening results, the hydroxyl-containing compounds **8**, **9**, and **10** showed the most significant anti-MRSA activity with a recorded inhibition zone ranging between 7.50–10.00 mm compared to the standard chlorhexidine (CHX) (14.00–15.00 mm). Although compounds **8**, **9**, and **10** exhibited lower anti-MRSA potential than the standard drug, CHX, we decided that it was still beneficial to proceed with the determination of their MIC and MBC values against the tested standard and clinical *S. aureus* strains. Meanwhile, the other molecules which compose the nitro- and fluoro- groups (compounds **1**–**7** and **11**–**15**) showed weak bacterial inhibition, signifying their poor antibacterial efficacy.

Owing to its strong bactericidal power and stability, the antiseptic CHX was used as the standard control in this experiment in comparison to other common anti-MRSA drugs including vancomycin, ampicillin, and streptomycin. Moreover, CHX is frequently used in hospitals to reduce MRSA infections, particularly for bathing patients in intensive care units and preventing the spread of hospital-acquired diseases [15] and thereby further justify its practical utilization.

The structure–activity relationship (SAR) revealed that the hydroxyl group, regardless of their position (*ortho*/*meta*/*para*) on the phenyl ring, was responsible for the improved MRSA inhibitory activity. The tendency of the -OH moiety to form a strong hydrogen bond to the important amino acid residues on the active sites of the receptor might have contributed to their significant antibacterial property. This result is in accordance with Stompor and Zarowska (2016) and Xu et al. (2019), which stated that the free hydroxyl group in position(s) *ortho* and/or *para* appears to be significant to anti-MRSA efficacy on its own, or in combination with antibiotics [16,17]. Inversely, the inhibition of *S. aureus* was found to be diminished in the electron-withdrawing substituted analogues, following the trend concluded by Kozlowska et al. (2019), Stompor and Zarowska (2016) and Ma et al. (2011), which suggested the lack of a hydroxyl group and the substitution of the halogen (Cl-, Br-) and nitro moieties into the chemical scaffold of interest may have resulted in the inactivation of the compounds with regards to *S. aureus* [16,18,19]. In addition, the mono- and new difluorinated chalcones (**2**–**4** and **11**–**15**) were designed and synthesized with the aim to increase the number of H-bond to the amino acid residues of targeted bacterial sites and thus improve the bioactivity, and to enhance the pharmacokinetic and pharmacodynamic (PK/PD) properties including the membrane permeability, solubility, metabolic pathways of the analogues [20]. However, such predicted improved properties were unable to significantly increase their targeted anti-MRSA efficacy, reflecting the importance of the electron-donating hydroxyl group as the key functional fragments that are responsible for the MRSA inhibition.

#### 2.2.2. Determination of Minimum Inhibitory Concentration (MIC) and Minimum Bactericidal Concentration (MBC)

The anti-MRSA activities of the most active molecules (compounds **8**, **9**, and **10**) were then further validated by determining their MIC and MBC values. Based on Table 1, compound **8** exhibited the most significant MIC and MBC values, whereby it only required a low concentration of 0.08 ± 0.0 mg/mL to inhibit the growth of the clinical bacteria 172918 strain and a slightly lower concentration (0.16 ± 0.0 mg/mL) to express either bactericidal or bacteriostatic effect. It is noteworthy to mention that the MIC and MBC results provide limited information on the kinetics of the antimicrobial action. Hence, the time-killing assay was performed to find a correlation between the rate of bactericidal activity with the incubation time and concentration of the antimicrobial agent [21]. On a separate note, though the recorded MIC and MBC values do not appear to be promising by current clinical standards, it should be highlighted that these preliminary results could be useful for subsequent hit-to-lead optimization to further improve the potency and bacterial resistance of the new compounds. Lastly, due to a shortage of sample availability of compounds **8**–**10** and their slightly lower diameter inhibition compared to other MRSA strains, no further MIC and MBC analyses against clinical bacteria 180620 strain were conducted.

#### 2.2.3. Determination of Time-Kill Curve

The most suitable method to determine the bactericidal or bacteriostatic mechanism of action is through the time-kill curve analysis. It is a reliable tool that is utilized to obtain information on the dynamic interaction between an antimicrobial agent and microbial strain [22]. The time-kill curve may also reveal the relationship between concentration and time in eliciting an antimicrobial effect. The antibacterial activity was measured as a reduction of the total count of CFU/mL by 99.9% (≥3 Log_10_) compared to the original bacterial inoculum [23]. The antimicrobial agent was viewed as bacteriostatic at the lowest concentration that diminishes the original inoculum size 0–3 Log_10_ CFU/mL, and bactericidal if the inoculum size was decreased by >3 Log_10_ CFU/mL [24,25].

The tested concentrations of compounds **8**, **9**, and **10** are 0× MIC, 1× MIC, 2× MIC, and 4× MIC, as listed in Table 2. The selected concentrations used are equal to the MIC, twice the MIC, and four times the MIC values of the tested compound. Plots of the time-kill curve for MRSA ATCC 700699 and 172918 of compounds **8**, **9**, and **10** at varying concentrations (0× MIC to 4× MIC) were constructed to determine the correlation between MIC and the bactericidal activity, respectively, in which each plot displayed the endpoint after four hours of incubation.

**Figure 2:**
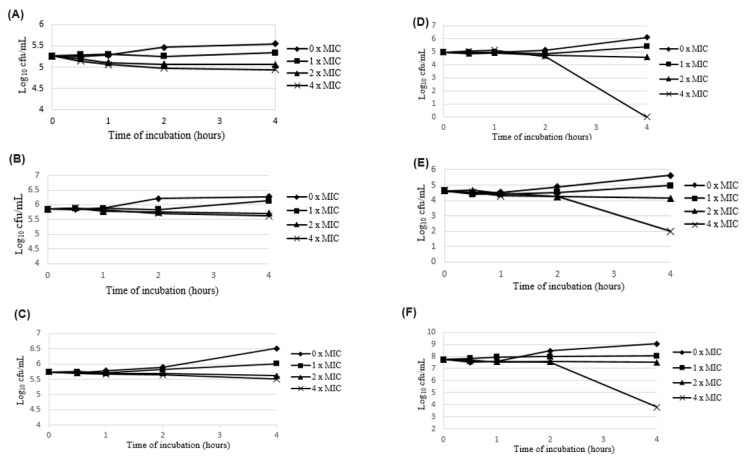
Time-kill curve of MRSA ATCC 700699 after exposure to (**A**) compound **8** and (**B**) compound **9** at 0× MIC (0 mg/mL), 1× MIC (0.156 mg/mL), 2× MIC (0.313 mg/mL), and 4× MIC (0.625 mg/mL), as well as (**C**) compound **10** at 0× MIC (0 mg/mL), 1× MIC (0.313 mg/mL), 2× MIC (0.625 mg/mL), and 4× MIC (1.25 mg/mL). Time-kill curve of clinical MRSA 172918 after exposure to (**D**) compound **8** at 0× MIC (0 mg/mL), 1× MIC (0.078 mg/mL), 2× MIC (0.156 mg/mL), and 4× MIC (0.312 mg/mL), (**E**) compound **9** at 0× MIC (0 mg/mL), 1× MIC (0.156 mg/mL), 2× MIC (0.313 mg/mL), and 4× MIC (0.625 mg/mL), and (**F**) compound **10** at 0× MIC (0 mg/mL), 1× MIC (0.313 mg/mL), 2× MIC (0.625 mg/mL), and 4× MIC (1.25 mg/mL).

The time-kill kinetic profiles of compounds **8**, **9**, and **10** (Figure 2A–C) displayed an abrupt decrease in the number of *S. aureus* ATCC 700699 colonies, in which a 3 Log_10_ reduction in viable cell count relative to the initial inoculum at 1× MIC value was observed after four hours’ exposure. Therefore, it is suggested that compounds **8**, **9**, and **10** may act as bactericidal agents based on the results. Furthermore, it was observed that compounds **8**, **9**, and **10** killed the standard MRSA ATCC and clinical MRSA172918 strains in a concentration-dependent manner (Figure 2A–F).

### 2.3. Molecular Docking Studies

Consequently, the bioactive compounds **8**, **9**, and **10** were docked into the active sites of PBP2a (PDB ID: 6Q9N) to elucidate the key molecular interactions that might be responsible for their significant anti-MRSA properties, and the results were compared to the standard drugs, CHX, and quinazoline. Two types of rigid docking procedures, the blind and site-specific, have been performed as tabulated in Table 3.

The generated results indicate binding affinities of −8.8 kcal/mol and −8.9 kcal/mol for the corresponding blind and site-specific docking analyses of quinazolinone allosteric inhibitor, an original ligand in the PBP2a crystals. Through the blind docking experiment, quinazolinone exhibited a similar pose and comparable binding affinity value as previously reported ligands in the same active site of the crystal proteins [26]. These findings, hence, validate the docking parameters utilized in this study.

**Figure 3:**
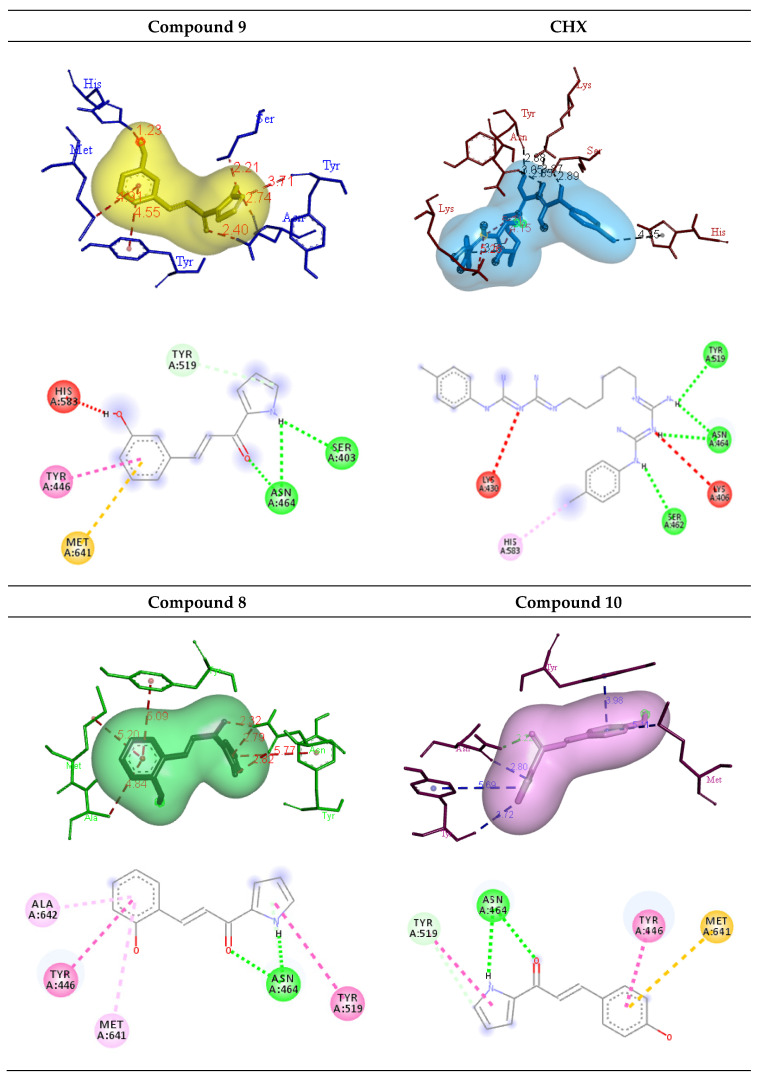
3D and 2D diagrams of the binding interaction of hydroxylated compounds **8**, **9**, and **10** (green line: H bond; light green line; carbon H bond/π-donor H bond; red line: donor-donor; yellow line: π-sulphur; pink line: π-π stacked/π-π T-shaped; light pink line: π-alkyl) and CHX (green line: H bond; red line: positive-positive; light purple line: π -alkyl) with the amino acid residues on the active site of the PBP2a receptor.

It is worth noting that compound **9** recorded the highest binding affinity at −7.0 kcal/mol among the bioactive hydroxylated analogues. Analogue **9** also showed almost similar interactions as CHX (−8.2 kcal/mol) with regards to the amino acid residues of the active sites of PBP2a, relative to **8** and **10**. Based on the molecular docking analysis (Figure 3), the -NH of pyrrole and carbonyl functionalities of compound **9** form multiple H-bonding with amino acid residues Ser403 and Asn464. Meanwhile, the pyrrole and phenyl moieties were C-H bonded (light green line) with the Tyr519 residue, as well as hydrophobically interacting with the Met641 and Tyr446 residues via π-sulphur and π-π stacking, respectively. All the binding interactions involved are tabulated in Table 4.

For the activity comparison, CHX was docked on the same active sites of PBP2a. Overall, CHX shows better affinity compared to compound **9**. Dong et al. (2019) suggested that the length of the alkyl chain and the number of guanidino groups on CHX may have contributed to a significant increase in inhibitory efficiency, specifically towards human O-GlcNAcase (hOGA), an enzyme that might be responsible for diabetes, cancer, and Alzheimer’s disease [27]. The presence of π-alkyl interactions could be responsible for the normalizing of the dipole moment of the drug by transferring the charge to its adjacent amino acids. According to the results, strong H-bond interactions (green dashed line) were recorded between the -NH functionality and Asn464, Tyr519, and Ser462 residues. Meanwhile, the π-alkyl interaction (light purple dashed line) between the chlorine atom and His 583 further strengthens the hydrophobic interaction of the ligand in the receptor-binding pocket.

Meanwhile, slightly lower binding affinities were observed in compounds **8** (−6.9 kcal/mol) and **10** (−6.7 kcal/mol) compared to compound **9**. Similarly, both compounds formed strong hydrogen bonds with Asn464 in the PBP2a binding site through the -NH of pyrrole and carbonyl moieties of the respective analogues **8** and **10** (Figure 3). However, it is presumed that a lack of an essential interaction with His583, together with the multiple hydrophobic interactions with the amino acid residues of PBP2a leads to the reduction in the binding affinity, thus lowering their anti-MRSA potential.

Hence, the morphological changes of the MRSA-bacterial cells when treated with this promising antimicrobial agent, analogue 9 was further investigated using the scanning electron microscopy (SEM) analysis.

### 2.4. Scanning Electron Microscope (SEM) Analysis

**Figure 4:**
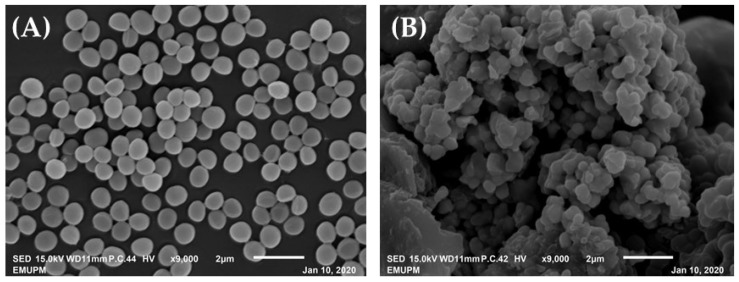
Scanning electron micrograph of (**A**) untreated and (**B**) treated with 1% of compound **9** on the standard MRSA ATCC 700699 cells for two hours.

**Figure 5:**
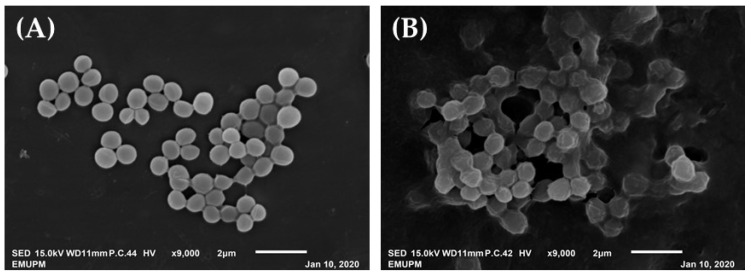
Scanning electron micrograph of (**A**) untreated and (**B**) treated with 1% of compound **9** on the clinical MRSA 172918 cells for two hours.

The cell structures of the untreated standard and clinical 172918 *S. aureus* strains are shown in Figure 4A and Figure 5A. These untreated cells served as a control and occurred as compact evenly shaped grape-like cluster cells with a round to oval cocci that have a smooth surface. Meanwhile, Figure 4B and Figure 5B illustrate the morphology of both standard and clinical 172918 *S. aureus* cells after a two-hour exposure to compound **9**. The bacterial cells were observed to be larger, irregular in shape, and partially disintegrated. Based on the results, it was determined that the bacterial morphology was altered from its smooth cell surface to shrunken and distorted after the treatment, suggesting the bactericidal property of compound **9**.

### 2.5. In Vivo Toxicity Profile in Zebrafish Model

We then employ an animal-based testing approach to preliminary evaluate the toxicologic profile of the most bioactive chemical in the series. Zebrafish (*Danio rerio*) have been extensively used as a toxicological model for drug screening purposes to treat various human diseases [28]. Based on the phylogenetic analyses of fish and human genomes, humans and zebrafishes share approximately 70% similar morphology and physiology in the nervous, cardiovascular, and digestive systems [29]. Additionally, the zebrafish genome is completely sequenced, and 84% of its genes were homologous to disease-related genes found in the human genome [30]. Furthermore, zebrafish can also provide valuable data for other vertebrate animals, allowing researchers to link biochemical, genetic, and cellular hypotheses to high-performance observations at structural, functional, and behavioural levels [31]. In the last two decades, the zebrafish embryotoxicity test or fish embryotoxicity test, has been gaining interest as it provides a total and well-defined developmental period for a vertebrate embryo, enabling the study of its early life stages [32]. Although both adults and embryos are proven useful for toxicology studies, embryos are more ideal and significant for toxicity profiling due to the transparency of the egg, which allows for easy detection and evaluation of the respective developmental phases and endpoints during the toxicity test [33,34]. The zebrafish model has technological and economic advantages over mammal models, particularly rodents [31,35] in reducing the number of substances and expenses required when using animals in drug tests and production [36].

To describe the toxic effects of analogue **9**, we first treated the zebrafish embryos with various dosages of the tested compound (10, 20, 40, 75, 150, 300, 600, and 1200 µM) starting from 6 hpf via the bath immersion technique. At 30 and 54 hpf, morphological comparisons were made between the exposed and control embryos to observe any morphological abnormalities.

**Figure 6:**
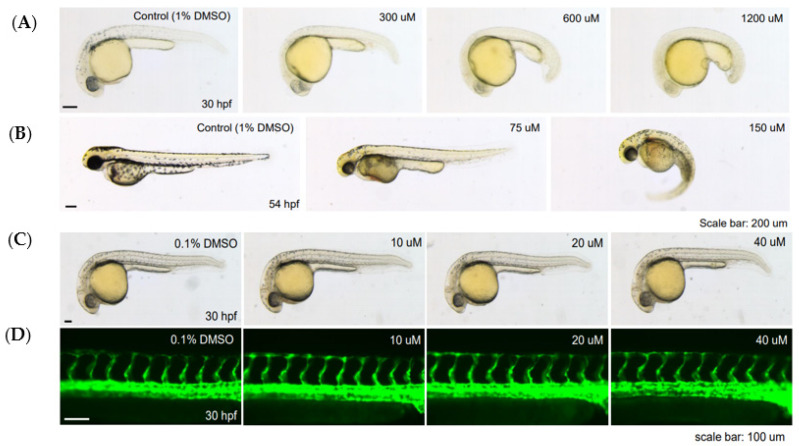
Morphological features of zebrafish embryos at different developmental stages. (**A**) At 30 hpf after a 24-h exposure to analogue **9** at concentrations of 300, 600, and 1200 µM, (**B**) at 54 hpf after a 48-h exposure to analogue **9** at concentrations of 75 and 150 µM, as well as (**C**) at 30 hpf after a 24-h exposure to analogue **9** at concentrations of 10, 20, and 40 µM. (**D**) Effects of analogue **9** at concentrations 10, 20, and 40 µM on blood vessel development in transgenic zebrafish, *Tg*(*kdrl:egfp*) at 30 hpf, same embryos as in (**C**). Vasculogenesis and angiogenesis in the trunk region were normal in both the control and analogue **9**-treated zebrafish.

Based on Figure 6C, the results revealed that the **9**-treated embryos were alive at 30 hpf with no significant morphological defects detected up to 40 µM in a 24-h treatment. We then further investigated the toxicity of compound **9** via a transgenic line of zebrafish that particularly expresses green fluorescent protein (GFP) in their vascular endothelial cells, *Tg*(*kdrl:egfp*) to gain significant insight into the pathological blood vessel formation of the embryos. No adverse effects were observed towards the normal development of the vasculature up to 40 µM on the 9-exposed late gastrula 10 hpf *Tg*(*kdrl:egfp*) embryos, as illustrated in Figure 6D. However, the assayed embryos show some developmental delays when treated with high concentrations; ≥300 µM in a 24-h treatment (Figure 6A) and ≥75 µM in a 48-h treatment of compound **9** (Figure 6B), respectively.

**Figure 7:**
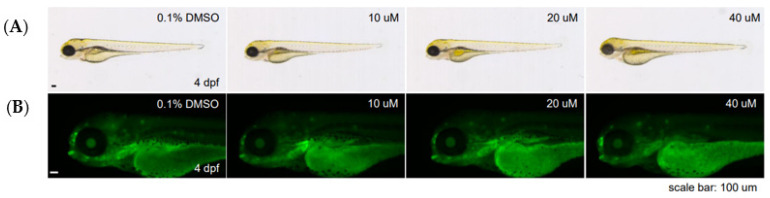
(**A**) Effects of compound **9** on later stage zebrafish larvae (4 dpf) at their respective concentrations of 10, 20, and 40 μM. (**B**) Detection of apoptosis or cell death via acridine orange, fluorescent vital dye staining. Non-specific signals in yolk and eye lens are autofluorescence shown in both the control and compound **9**-treated animals.

To further assess the apoptotic properties of **9**, the treated embryos were assayed at 10, 20, and 40 µM concentrations in a 24-h treatment via a fluorescent vital dye, acridine orange (AO) to mark dead and/or apoptotic cells at 96 hpf. Apoptosis is an essential component in a normal developmental process of various tissues and organ systems and is often characterized by a specific cellular shrinkage, membrane blebbing, nuclear condensation, and nuclear fragmentation, all of which are well-known hallmarks of this type of cell death [37]. Based on Figure 7, the AO-positive cells were sparsely detected in the bodies of the control and **9**-treated transgenic zebrafish at all tested concentrations in a similar pattern. The results signify that compound **9** has no negative interventions on the normal development of zebrafish embryos compared to the DMSO-vehicle control larvae.

## 3. Materials and Methods

### 3.1. In Silico Pan-Assay Interference Compounds (PAINS) Identification

All pyrrolylated-chalcone chemical structures were drawn using ChemDraw Ultra 12.0 and subsequently converted to SMILES (Simplified Molecular Input Line Entry System) format. The SMILES formulas were then converted into Symyx SDF (Spatial Data File) file format as recommended by the FAF-Drugs4 Bank Formatter (http://mobyle.rpbs.univ-paris-diderot.fr/cgi-bin/portal.py#forms::Bank-Formatter, accessed on 30 September 2020), and subsequently submitted into the FAF-Drugs4 server (http://mobyle.rpbs.univ-paris-diderot.fr/cgi-bin/portal.py#forms::FAF-Drugs4, accessed on 30 September 2020) for the filtration of PAINS using the PAINS filters A, B, and C [38]. The generated results are available at the following address (https://mobyle.rpbs.univ-paris-diderot.fr/cgi-bin/portal.py?form=admetox#jobs::FAF-Drugs4.U06888724708080, accessed on 30 September 2020).

### 3.2. General

All the chemicals and reagents used in the synthesis and biological evaluation experiments were purchased from Merck (Kenilworth, NJ, USA), Alfa Aesar (Haverhill, MA, USA), Sigma Aldrich (St. Louis, MO, USA), Acros Organics (Carlsbad, CA, USA), and Systerm (Shah Alam, Malaysia). The melting points of the synthesized compounds were determined using XSP-12 model 500× Fisher–Johns aluminium heating stage melting point apparatus equipped with an adjustable magnifier Model 12-144 (American Laboratory Trading, Cambridge, MA, USA). 1D (^1^H-NMR, ^13^C-NMR, and ^19^F-NMR) and 2D (COSY, HSQC, and HMBC) NMR spectra were recorded using Bruker AV 500 MHz and Bruker’s 700 MHz ASCEND^TM^ NMR spectrometer (Bruker, Karlsruhe, Germany). The mass spectra for known compounds (including the new analogue **5**, due to the sample unavailability for UHPLC-ESI-HRMS) were determined using a Shimadzu GCMS-QP2010 Plus (Shimadzu, Kyoto, Japan), whilst the UHPLC-ESI-HRMS spectra of the new analogues **11**–**15** was measured with a Thermo Fisher Scientific^TM^ Model Q Exactive^TM^ Hybrid Quadrupole-Orbitrap mass spectrometer (Thermo Fisher Scientific, Waltham, MA, USA), in which both analyses were performed via direct injection. The FT-IR spectrum was recorded on a Perkin Elmer RX1 FT-IR spectrometer (Perkin Elmer, Waltham, MA, USA) in the mid-IR region (400–4000 cm^−1^). The methicillin-resistant *S. aureus* (MRSA) ATCC 700699 was purchased from American Type Culture Collection (ATCC) (ATCC, Manassas, VA, USA), while the three clinical MRSA strains (172918, 180620, and 180865) were obtained from the Microbial Collection of the Natural Medicines and Product Research Laboratory, Institute of Bioscience, UPM.

### 3.3. Synthesis

The known pyrrolylated-chalcones (**1**–**4** and **6**–**10**) were synthesized following the method as described by Mohd Faudzi et al. (2020) [9]. The general and spectrometry data, including the percentage yield and melting point, of the synthesized molecules **1**–**15** was tabulated in Table S1 of Supplementary Materials.

#### 3.3.1. General Procedure for the Synthesis of New 3-(2-Nitrophenyl)-1-(1*H*-pyrrol-2-yl) Prop-2-en-1-one (**5**)

Firstly, 10 mL of NaOH (6 M) was dissolved in 6 mL of water and cooled at 0 °C in an ice bath, followed by the addition of 10 mL of 95% ethanol into the diluted NaOH solution, of which was carried out slowly. The reaction flask was then removed from the ice bath and was left at room temperature. Following that, an equimolar quantity of 2-acetylpyrrole (1.091 g/1.00 mmol) and 2-nitrobenzaldehyde (0.1511 g/1.00 mmol) was slowly added to the mixture. The reaction mixture was stirred for 2 h at room temperature and cooled at 0 °C for 24 h. The precipitates formed were filtered, washed with cold water, and recrystallized with ethanol. The resulting product was a grey cotton (2.90%); mp 193–194 °C; IR (UATR) 3240 (NH), 1643 (C=O), 1581 (C=C), 1508 and 1404 (NO_2_) cm^−1^; ^1^H NMR (700 MHz, DMSO-*d*_6_) δ: 6.37 (*m*, 1H, Py-H), 7.37 (*t*, *J* = 3.1 Hz, 1H, Py-H), 7.42 (*td*, *J* = 1.1 and 7.4 Hz, 1H, Ar-H), 7.64 (*s*, 1H, Py-H), 7.77 (*td*, *J* = 1.2 and 7.6 Hz, 1H, Ar-H), 8.00 (*dd*, *J* = 1.6 and 7.6 Hz, 1H, Ar-H), 8.63 (*d*, *J* = 8.3 Hz, 1H, Ar-H), 10.0 (*s*, 1H, NH), 12.30 (*br. s*, 2H, CH=CH); ^13^C NMR (175 MHz, DMSO-*d*_6_) δ: 112.2, 120.0, 123.7, 124.9, 128.6, 130.2, 136.2, 136.6, 138.6, 161.2, 173.5, 196.5; *m*/*z* (DI-MS) 242 (M^+^, C_13_H_10_N_2_O_3_ requires 242).

#### 3.3.2. General Procedure for the Synthesis of New Difluorinated Compounds (**11**–**15**)

Two mmol of 2-acetylpyrrole and substituted benzaldehyde were dissolved in 5 mL of 95% ethanol and the mixture was stirred for 5 min, followed by a dropwise addition of 1 mL of NaOH (6 M). The reaction mixture was then left to stir overnight at room temperature. Once the reaction was completed, crushed ice was added to quench the reaction. Following that, diluted HCl was added to neutralize the reaction mixture before being further extracted with ethyl acetate (3 × 10 mL). The organic layer was collected, dried with anhydrous MgSO_4_, and evaporated under reduced pressure. The crude products were purified by column chromatography and/or recrystallization techniques to yield pure compounds [9]. The chemical characterization was only discussed for the newly synthesized molecules.

##### 3-(2,3-Difluorophenyl)-1-(1*H*-pyrrol-2-yl) Prop-2-en-1-one (**11**)

Following the general procedure stated in Section 3.3.2, 2-acetylpyrrole (0.2183 g/2.00 mmol) reacted with 2,3-difluorobenzaldehyde (218 μL/2.00 mmol) and was recrystallized with ethanol. The resulting product was yellow in colour (65.37%); mp 142–143 °C; IR (UATR) 3230 (NH), 1643 (C=O), 1579 (C=C), 1280 (C-F) cm^−1^; ^1^H NMR (500 MHz, acetone-*d*_6_) δ: 6.34 (*m*, 1H, Py-H), 7.27 (*m*, 1H, Py-H), 7.30 (*m*, 1H, Ar-H), 7.32 (*m*, 1H, Py-H), 7.40 (*m*, 1H, Ar-H), 7.75 (*d*, *J* = 15.9 Hz, 1H, CH=CH), 7.78 (*dd*, *J* = 1.5 and 6.5 Hz, 1H, Ar-H), 7.85 (*d*, *J* = 15.8, 1H, CH=CH), 11.15 (*br. s*, 1H, NH); ^13^C NMR (125 MHz, acetone-*d*_6_) δ: 110.3, 116.9, 118.2, 123.8, 124.8, 125.4, 126.2, 131.2, 133.2, 148.9, 150.7, 177.2; ^19^F NMR (470 MHz, acetone-*d*_6_) δ: −140.2 and −143.6; HRMS (ESI): *m*/*z* calcd for C_13_H_9_F_2_NO-H^−^: 232.0580 [M − H]^−^; found: 232.0577.

##### 3-(2,4-Difluorophenyl)-1-(1*H*-pyrrol-2-yl) Prop-2-en-1-one (**12**)

Following the general procedure stated in Section 3.3.2, 2-acetylpyrrole (0.2183 g/2.00 mmol) reacted with 2,4-difluorobenzaldehyde (218 μL/1.99 mmol) and was purified with column chromatography, Hexane: EtOAc (7:3) and recrystallized with acetone. The resulting product was a pale white solid (90.23%); mp 118–119 °C; IR (UATR) 3251 (NH), 1643 (C=O), 1571 (C=C), 1273 (C-F) cm^−1^; ^1^H NMR (500 MHz, acetone-*d*_6_) δ: 6.33 (*br. s*, 1H, Py-H), 7.15 (*m*, 2H, Ar-H), 7.26 (*br. s*, 1H, Py-H), 7.29 (*br. s*, 1H, Py-H), 7.70 (*d*, *J* = 15.8, 1H, CH=CH), 7.83 (*d*, *J* = 15.8, 1H, CH=CH), 8.06 (*dd*, *J* = 7.1 and 8.3 Hz 1H, Ar-H), 11.13 (*br. s*, 1H, NH); ^13^C NMR (125 MHz, acetone-*d*_6_) δ: 104.2, 110.2, 112.1, 116.7, 119.8, 124.6, 125.9, 130.3, 131.5, 133.2, 161.6, 163.8, 177.4; ^19^F NMR (470 MHz, acetone-*d*_6_) δ: −108.3 and −112.9; HRMS (ESI): *m*/*z* calcd for C_13_H_9_F_2_NO-H^−^: 232.0580 [M − H]^−^; found: 232.0579.

##### 3-(2,5-Difluorophenyl)-1-(1*H*-pyrrol-2-yl) Prop-2-en-1-one (**13**)

Following the general procedure stated in Section 3.3.2, 2-acetylpyrrole (0.2183 g/2.00 mmol) reacted with 2,5-difluorobenzaldehyde (218 μL/2.00 mmol) and was recrystallized with methanol. The resulting product was a pale white solid (85.28%); mp 124–125 °C; IR (UATR) 3228 (NH), 1651 (C=O), 1487 (C=C), 1271 (C-F) cm^−1^; ^1^H NMR (500 MHz, acetone-*d*_6_) δ: 6.34 (*m*, 1H, Py-H), 7.28 (*m*, 1H, Py-H), 7.31 (*dd*, *J* = 4.7 and 9.2 Hz, 2H, Ar-H), 7.36 (*m*, 1H, Py-H), 7.78 (*d*, *J* = 15.8, 1H, CH=CH), 7.81 (*m*, 1H, Py-H), 7.84 (*d*, *J* = 16.1, 1H, CH=CH), 11.14 (*br*. *s*, 1H, NH); ^13^C NMR (125 MHz, acetone-*d*_6_) δ: 110.3, 114.2, 117.0, 117.5, 118.1, 124.6, 126.1, 126.2, 131.1, 133.2, 157.2, 159.2, 177.2; ^19^F NMR (470 MHz, acetone-*d*_6_) δ: −119.8 and −123.4; HRMS (ESI): *m*/*z* calcd for C_13_H_9_F_2_NO-H^−^: 232.0580 [M − H]^−^; found: 232.0570.

##### 3-(3,4-Difluorophenyl)-1-(1*H*-pyrrol-2-yl) Prop-2-en-1-one (**14**)

Following the general procedure stated in Section 3.3.2, 2-acetylpyrrole (0.2183 g/2.00 mmol) reacted with 3,4-difluorobenzaldehyde (218 μL/1.98 mmol) and was recrystallized with EtOH: Acetone (5:5). The resulting product was a yellow solid (47.51%); mp 152–153 °C; IR (UATR) 3253 (NH), 1645 (C=O), 1570 (C=C), 1269 (C-F) cm^−1^; ^1^H NMR (500 MHz, acetone-*d*_6_) δ: 6.33 (m, 1H, Py-H), 7.24 (m, 1H, Py-H), 7.34 (m, 1H, Py-H), 7.42 (m, 1H, Ar-H), 7.64 (m, 1H, Ar-H), 7.66 (d, *J* = 16.1, 1H, CH=CH), 7.70 (d, *J* = 15.7, 1H, CH=CH), 7.88 (m, 1H, Ar-H), 11.08 (br. s, 1H, NH); ^13^C NMR (125 MHz, acetone-*d*_6_) δ: 110.2, 116.3, 116.8, 117.7, 124.0, 125.7, 125.8, 133.2, 133.3, 138.4, 149.7, 151.7, 177.4; ^19^F NMR (470 MHz, acetone-*d*_6_) δ: −137.6 and −139.5; HRMS (ESI): *m*/*z* calcd for C_13_H_9_F_2_NO-H^−^: 232.0580 [M − H]^−^; found: 232.0569.

##### 3-(3,5-Difluorophenyl)-1-(1*H*-pyrrol-2-yl) Prop-2-en-1-one (**15**)

Following the general procedure stated in Section 3.3.2, 2-acetylpyrrole (0.2183 g/2.00 mmol) reacted with 3,5-difluorobenzaldehyde (218 μL/1.99 mmol) and was recrystallized with methanol. The resulting product was a yellow solid (69.72%); mp 151–152 °C; IR (UATR) 3243 (NH), 1649 (C=O), 1581 (C=C), 1301 (C-F) cm^−1^; ^1^H NMR (500 MHz, acetone-*d*_6_) δ: 6.34 (*m*, 1H, Py-H), 7.09 (*tt*, J = 2.3 and 9.1 Hz 1H, Ar-H), 7.26 (*m*, 1H, Py-H), 7.39 (*m*, 1H, Py-H), 7.50 (*br. d*, *J* = 2.1, 1H, Ar-H), 7.52 (*br d*, *J* = 2.1, 1H, Ar-H), 7.67 (*d*, *J* = 15.7, 1H, CH=CH), 7.79 (*d*, *J* = 15.7, 1H, CH=CH), 11.10 (*br. s*, 1H, NH); ^13^C NMR (125 MHz, acetone-*d*_6_) δ: 104.5, 110.3, 110.9, 111.1, 117.0, 125.5, 126.1, 133.3, 138.1, 139.3, 162.3, 164.3, 177.3; ^19^F NMR (470 MHz, acetone-*d*_6_) δ: −111.1; HRMS (ESI): *m*/*z* calcd for C_13_H_9_F_2_NO-H^−^: 232.0580 [M − H]^−^; found: 232.0569.

### 3.4. In Vitro Evaluation of the Anti-MRSA Activity

#### 3.4.1. Disc Diffusion Assay

The synthesized compounds were screened for their anti-MRSA potential against the standard ATCC and three clinical MRSA bacterial strains via a disc diffusion assay (DDA). The DDA was conducted following the method suggested by the Clinical and Laboratory Standard Institute (CLSI) 2012 [39]. A single uniform colony of inoculum was prepared and immediately spread on an MHA plate with a sterile cotton swab. Sterile filter paper discs with a diameter of 6 mm were placed on top of the culture, each paper discs were permeated with 10 µL of the respective test compounds (1 mg/100 µL) at a 1% concentration. For activity comparisons, 0.1% CHX and 10% DMSO were used as positive and negative controls. After a 24-h incubation time at 37 °C, the inhibition zones were measured and recorded in millimetres. The assay was carried out once in duplicates (*n* = 1 × 2).

#### 3.4.2. Determination of Minimum Inhibitory Concentration and Minimum Bactericidal Concentration

The minimum inhibitory concentration (MIC) was determined by a two-fold broth microdilution method using a 96-well round-bottom microtiter plate (Greiner Bio-One, Frickenhausen, Germany) with an inoculum suspension of all the bacteria strains at approximately 10^6^ to 10^8^ CFU/mL [21]. Each well on the first column was filled with 100 μL of MHB, which serves as the negative growth control, while each well on the second column was filled with 100 μL of the bacterial suspension as a positive control for cell growth. The microdilution was performed at varying concentrations of the test samples, ranging from 5 mg/mL (in column 12) to 0.01 mg/mL (in column 3). The concentrations of the positive control, CHX ranged from 500 μg/mL (in column 12) to 0.97 μg/mL (in column 3). Subsequently, the plates were incubated for 24 h at 37 °C. The MIC value was determined by the lowest compound concentration that produces no visible growth in the wells [40].

Following that, the minimum bactericidal concentration (MBC) was determined by sub-culturing the 10 µL suspension from each MIC well (columns 1–12) onto an MHA plate. The plates were then incubated at 37 °C for 24 h or until bacteria growth was observed at the growth control plates. MBC was defined as the minimum concentration of tested samples that is needed to kill bacteria on the MHA plates. The MIC and MBC values were expressed as the means ± standard deviation (SD); *n* = 2 × 3.

#### 3.4.3. Determination of Time-Kill Curve

The time-kill assay for compounds **8**, **9**, and **10** was carried out against the ATCC and clinical MRSA strains according to the method described by the CLSI (2012) [39], with a few modifications. Firstly, approximately 10^6^ CFU/mL of inoculum suspension was prepared and was used to dilute the tested compounds to obtain final concentrations of 0× MIC, 1× MIC, 2× MIC, and 4× MIC for each bacterial strain. Then, 10 µL of each solution (a compound with inoculum suspension) at 0 h, 0.5 h, 1 h, 2 h, and 4 h were serially diluted (10-6-10-2) with 1% of PBS and spread onto the MHA plates using a sterile cotton swab with replicates (*n* = 1 × 2) and was incubated at 37 °C overnight. After 24 h, the number of colonies present on the plates were measured and recorded as CFU/mL and a Log_10_ CFU/mL over time graph was plotted. The results obtained signifies bactericidal activity, whereby a reduction of 99.9% (≥3 Log_10_) of the total number of CFU/mL in the original inoculum was expected. The time-kill assay was carried out twice in triplicates (*n* = 2 × 3).

### 3.5. Molecular Docking Studies

The crystallographic structure of Penicillin-Binding protein 2a (PBP2a) from MRSA ATCC700699 was retrieved from the Protein Data Bank (www.rcsb.org; PDB ID: 6Q9N. accessed on 25 February 2021). Molecular docking was performed to elucidate the possible binding interactions of the most active compounds (compounds **8**, **9**, and **10**) to the standard drug, chlorhexidine (CHX) with the MRSA protein using AutoDockTools 1.5.4 (http://mgltools.scripps.edu, accessed on 25 February 2021) and AutoDock Vina (http://vina.scripps.edu, accessed on 25 February 2021) programs. The ligand and water molecules were removed, and hydrogen atoms were added to the 6Q9N.pdb crystal structure before the docking analysis. The water molecules removal step was performed to ease the computations and to clear the binding site that can distort the search [10]. AutoDockTools 1.5.4 was used to add the missing hydrogen atoms for the formation of the hydrogen bond network leading to molecular docking [41]. The 3D structure of the most active inhibitors (compounds **8**, **9**, and **10**) was built using Avagadro while the 3D structure of the standard drug, CHX was obtained from the PubChem website (https://pubchem.ncbi.nlm.nih.gov, accessed on 25 February 2021). These structures were geometrically optimized by energy minimization using the General AMBER Force Field (GAFF) potential function and were prepared by assigning the non-polar hydrogen using AutoDockTools 1.5.4. AutoDockTools 1.5.4 was used to generate the docking input files and to analyze the rigid docking results. Docking calculations were performed with an automated docking software, AutoDock Vina [42]. The final output obtained from the docking procedure is a set of conformations ranked correspondingly to their scoring function values. Meanwhile, Discovery Studio Visualizer 4.0 was used to visualize the interaction between enzymes and ligands in both 2D and 3D representations.

### 3.6. Scanning Electronic Microscope Analysis

Fresh methicillin-resistant *S. aureus* (MRSA) ATTC700699 and three clinical isolates (172918, 180620, and 180865) of *S. aureus* bacteria were treated with compound **9** and incubated at 37 °C in MHB for one hour. The pellets were collected by ALC microcentrifuge 4214 at 12,000× *g* for 10 min (ALC, Milan, Italy) and were fixed with 2.5% glutaraldehyde for 4–6 h at 4 °C. Then, the pellets were washed with 0.1 M sodium cacodylate buffer for 10 min thrice. Fixing of the pellets was performed using 1% osmium tetroxide for two hours at 4 °C and re-washed thrice with 0.1 M sodium cacodylate buffer for 10 min each time. Following that, the pellets were dehydrated using 35, 50, 75, and 95% acetone for 10 min each concentration. Finally, the pellets were dehydrated for 15 min using 100% acetone, and this process was repeated three times. The cell suspension was transferred into an albumin-coated aluminium foil specimen basket and placed in a critical dryer for 30 min. The specimen was then stacked on a stub and then sputter-coated with gold. Examination of the bacterial morphology was observed using a JEOL JSM 6400 scanning electron microscope (JEOL Ltd., Tokyo, Japan) and the images were captured.

### 3.7. In Vivo Toxicity in Zebrafish Embryos

#### 3.7.1. Zebrafish Husbandry and Embryo Collection

The embryos were produced through natural mating of wild-type (WT) and transgenic *Tg*(*kdrl:egfp*) adult zebrafish provided by the Zebrafish Center for Disease Modeling, Korea. The mated zebrafish were kept in a mixed male and female enclosure (ratio of 2:3), with 14 h of light: 10 h of darkness-controlled photoperiod within an ambient temperature of 28.5 °C. The embryos were collected 30 min after the onset of light, washed with distilled water, rinsed with embryo media (15 mM NaCl, 0.5 mM KCl, 1 mM MgSO_4_, 0.15 mM KH_2_PO_4_, 0.05 mM Na_2_HPO_4_, 1 mM CaCl_2_, 0.7 mM NaHCO_3_, pH 7.0), and incubated at 28 °C. The unfertilized embryos were discarded to prevent fungal development. The fertilized embryos were examined and chosen under a standard dissection Leica S6E microscope (Leica, Wetzlar, Germany). All animal experiments were carried out in accordance with the approved guidelines and regulations of the Institutional Animal Care and Use Committee (IACUC) at Chungnam National University [43].

#### 3.7.2. Acute Toxicity Testing on Zebrafish Embryos

To assess the acute toxicity on early embryonic growth, ten zebrafish embryos at 6 hpf were inserted into each well of a 24-well plate, containing 1 mL of embryo medium. Analogue **9** was firstly dissolved in DMSO to prepare a 40 mM stock solution and further diluted with embryonic water at 10, 20, 40, 75, 150, 300, 600, and 1200 uM concentrations. The compound solution was changed every day to keep the solutions fresh. To procure microscopic images, zebrafish embryos were dechorionated using forceps, anesthetized in Tricaine (Sigma-Aldrich, St. Louis, MO, USA), and placed in 3% methylcellulose. Embryos were viewed via a Leica M205 FCA microscope (Leica, Wetzlar, Germany), while the digital images were captured using a Leica DFC450C digital camera (Leica) and processed with the Leica Application Suite (Leica, Wetzlar, Germany) [43].

#### 3.7.3. Evaluation of Abnormalities on the Formation of Blood Vessels

Zebrafish embryos collected from the blood vessel-specific EGFP fluorescent transgenic zebrafish line, *Tg*(*kdrl:egfp*), were either treated with 0.1% DMSO as a control or treated with different concentrations (10, 20, and 40 µM) of compound **9**. The exposure to **9** commenced from the late gastrula stage at 10 hpf and was examined at 30 hpf when normal blood vessel formation was prominent in the control zebrafish. For bioimaging purposes, the assayed embryos were placed in 3% methylcellulose on a glass slide and live animal images were taken by a CELENA^®^ S Digital Imaging System (Logos Biosystems, Anyang, Korea) [43].

#### 3.7.4. Evaluation of Apoptosis

The measurement of cell death in the wild-type zebrafish larvae after exposure to compound **9** was performed using the vital fluorescent dye acridine orange (AO) method, which is commonly used as a marker of apoptotic cells in zebrafish. AO staining is a rapid method that has been shown to be highly selective for apoptotic cells in zebrafish embryos. The live zebrafish larvae were firstly immersed in the egg water containing 4 µg/mL of AO (Sigma-Aldrich, St. Louis, MO, USA) for 20 min, washed with the egg water five times for 5 min each, anesthetized with tricaine and mounted in 3% methyµcellulose, before being examined by stereomicroscopy and fluorescent microscopy [44,45].

#### 3.7.5. Statistical Analysis

Statistical analyses were conducted using SPSS (SPSS v. 25.0). The effects of each treatment group over the control were determined based on a one-way analysis of variance (ANOVA). Data were represented as the mean ± standard error mean using GraphPad Prism (GraphPad Software, Inc., San Diego, CA, USA), and were significantly different when *p* ≤ 0.05. To ensure the generated values were independent, the analysed data were based on a per well basis to prevent any interaction biases among the embryos [42].

## 4. Conclusions

In conclusion, a series of non-PAINS related pyrrolylated-chalcone analogues (**1**–**15**) were successfully synthesized and tested for their potential as anti-MRSA agents in vitro. The results revealed that the hydroxyl-containing pyrrolylated-chalcones (**8**, **9**, and **10**) exhibit remarkable MRSA inhibition. Furthermore, the time-kill curve plot shows that MRSA strains treated with compound **9** resulted in the death of all bacterial cells within four hours. The docking results revealed that ligand **9** showed similar bonding interactions to the standard drug, chlorhexidine (CHX) in the PBP2a active site, with their binding affinity scores of −7.0 kcal/mol and −8.2 kcal/mol, respectively. Further validations with SEM highlight the bactericidal characteristic of compound **9**, as the evenly shaped *S. aureus* cells were turned into irregular shapes and were also partially disintegrated, post-treatment. In addition, no harmful effects were observed in the normal embryonic development, blood vessel formation, and apoptosis processes during a 24-h in vivo zebrafish model treatment with up to 40 µM of compound **9**. Results of the study suggest that compound **9** (meta-hydroxy) is a promising compound for the inhibition of MRSA. Furthermore, all the collected data can be used as preliminary information for hit-to-lead optimization, enzyme-based in vitro efficiency measurement, in vivo efficacy, and safety assessment in bigger mammalian models and mechanisms of action to speed up the discovery of new antibiotics.

## Figures and Tables

**Table 1:** In vitro anti-MRSA activity evaluation of pyrrolylated-chalcone analogues (**1**–**15**) against standard and clinical *Staphylococcus aureus* strains.

	MRSA ATCC (700699)			Clinical MRSA (172918)			Clinical MRSA (180620)			Clinical MRSA (180865)		
	Zone of Inhibition (mm)	MIC (mg/mL)	MBC (mg/mL)	Zone of Inhibition (mm)	MIC (mg/mL)	MBC (mg/mL)	Zone of Inhibition (mm)	MIC (mg/mL)	MBC (mg/mL)	Zone of Inhibition (mm)	MIC (mg/mL)	MBC (mg/mL)
**1**	n.a	-	-	n.a	-	-	n.a	-	-	n.a	-	-
**2**	n.a	-	-	n.a	-	-	n.a	-	-	n.a	-	-
**3**	n.a	-	-	n.a	-	-	n.a	-	-	n.a	-	-
**4**	n.a	-	-	n.a	-	-	n.a	-	-	n.a	-	-
**5**	n.a	-	-	n.a	-	-	n.a	-	-	n.a	-	-
**6**	n.a	-	-	n.a	-	-	n.a	-	-	n.a	-	-
**7**	n.a	-	-	n.a	-	-	n.a	-	-	n.a	-	-
**8**	9.00 ± 1.4	0.23 ± 0.1	0.31 ± 0.0	8.50 ± 0.7	0.08 ± 0.0	0.16 ± 0.0	8.50 ± 0.7	^#^	^#^	8.00 ± 0.0	0.70 ± 0.8	0.94 ± 0.4
**9**	8.00 ± 1.4	0.23 ± 0.1	0.47 ± 0.2	8.50 ± 0.7	0.70 ± 0.8	1.41 ± 1.6	9.00 ± 0.0	^#^	^#^	9.00 ± 0.0	0.31 ± 0.0	0.63 ± 0.0
**10**	9.00 ± 0.0	0.47 ± 0.2	1.56 ± 1.3	8.50 ± 0.7	0.31 ± 0.0	1.88 ± 0.9	10.00 ± 0.0	^#^	^#^	7.50 ± 0.0	0.23 ± 0.1	1.88 ± 0.9
**11**	n.a	-	-	n.a	-	-	n.a	-	-	n.a	-	-
**12**	n.a	-	-	n.a	-	-	n.a	-	-	n.a	-	-
**13**	n.a	-	-	n.a	-	-	n.a	-	-	n.a	-	-
**14**	n.a	-	-	n.a	-	-	n.a	-	-	n.a	-	-
**15**	n.a	-	-	n.a	-	-	n.a	-	-	n.a	-	-
CHX *	14.00 ± 1.4	0.03 ± 0.0	0.20 ± 0.2	14.50 ± 0.7	0.03 ± 0.0	0.07 ± 0.1	15.00 ± 0.0	-	-	14.50 ± 0.7	0.12 ± 0.1	0.23 ± 0.1

n.a: not active (no inhibition). * Standard drug, chlorohexidine was used for the anti-MRSA assay (concentration of 1%). ^#^ Due to sample availability and complex purification of compounds **8**–**10**, as well as their slightly lower diameter inhibition compared to other MRSA strains, therefore, these molecules were not tested against clinical bacteria 180620 strain for MIC and MBC analyses.

**Table 2:** Concentrations of compounds **8**, **9**, and **10** used (0 × MIC, 1 × MIC, 2 × MIC and 4 × MIC) for the time-kill test.

Compound	*S. aureus* Strain	0× MIC (mg/mL)	1× MIC * (mg/mL)	2× MIC (mg/mL)	4× MIC (mg/mL)
**8**	MRSA ATCC 700699	0	0.156	0.313	0.625
	Clinical MRSA 172918	0	0.078	0.156	0.313
**9**	MRSA ATCC 700699	0	0.156	0.313	0.625
	Clinical MRSA 172918	0	0.156	0.313	0.625
**10**	MRSA ATCC 700699	0	0.313	0.625	1.250
	Clinical MRSA 172918	0	0.313	0.625	1.250

* The MIC values used were obtained through the MIC assay and are tabulated in Table 1.

**Table 3:** Concentrations of compounds **8**, **9**, and **10** used (0 × MIC, 1 × MIC, 2 × MIC and 4 × MIC) for the time-kill test.

Blind Docking					
Compound	8	9	10	CHX	Quinazolinone
Binding affinity (Kcal/mol)	−6.9	−7.0	−6.7	−7.0	−8.8
Amino acid residues	Ser461 Tyr 446 Met 641 Asn 464 Tyr 519	Tyr 446 Met 641 Ser 403 Asn 464 Tyr 519 His 583	Tyr 366 Gln 396 His 251 Lys 281 Lys 285 Leu 286 Pro 497	Gly 282 Lys 281 Phe 498 Asp 491 Ser 494 Ile 492 Leu 286 Pro 497	Asn 236 Phe 211 Thr 210 Trp 205 Pro 213 Glu 170
Site-Specific Docking					
Binding affinity (Kcal/mol)	−6.9	−7.0	−6.7	−8.2	−8.9
Amino acid residues	Ala 642 Tyr 446 Met 641 Asn 464 Tyr 519	Tyr 446 Met 641 Asn 464 Tyr 519	Tyr 446 Met 641 Asn 464 Tyr 519	Lys 430 Lys 406 Ser 462 Asn 464 Tyr 519 His 583	Asn 464 Gln 521 Tyr 446 Met 641 His 583 Gln 613

**Table 4:** Data of interactions resulting from the molecular docking of compounds **8**, **9**, **10**, and CHX into PBP2a.

Compound 9				CHX			
Amino Acid Residue	Interactions	With	Bonding Distance (Å)	Amino Acid Residue	Interactions	With	Bonding Distance (Å)
Ser403	H-Bond	NH	2.21	Lys430	Unfavorable positive-positive	NH	5.26
Asn464	H-Bond	NH	2.74	Asn464	H-Bond	NH	3.05
	H-Bond	C=O	2.40		H-Bond	NH	2.85
Tyr519	C-H bond	Pyrrole	3.71	Tyr519	H-Bond	NH	2.88
His583	Unfavorable Donor-donor	OH	1.23	His583	π-alkyl	Cl	4.35
Met641	π-sulphur	Benzene	4.91	Lys406	Unfavorable positive-positive	NH	3.87
Tyr446	π-π stacked	Benzene	4.55	Ser462	H-Bond	NH	2.89
**Compound 8**				**Compound 10**			
Ala642	π-alkyl	Benzene	4.84	Tyr446	π-π T-shapedπ-π stacked	Benzene	3.98
Tyr446	π-π T-shaped	Benzene	5.09	Met641	π-sulphur	Benzene	5.11
Met641	π-alkyl	Benzene	5.20	Asn464	H-Bond	NH	2.80
Asn464	H-Bond	NH	2.82		H-Bond	C=O	2.22
	H-Bond	C=O	2.32	Tyr519	C-H Bond	Pyrrole	5.69
	π-donor H-Bond	Pyrrole	2.79		π-π T-shapedπ-π stacked	Pyrrole	3.72
Tyr519	π-π T-shaped	Pyrrole	5.77				

## Data Availability

Not applicable.

## Supplementary Materials

The following are available online. Table S1: Spectrometric and general data of pyrrolylated-chalcone analogues (**1**–**15**), Table S2: The Lipinski rule values of pyrrolylated-chalcone analogues generated from FAF-Drugs4.

## Sample Availability

Not available.
