# Supplementary material for: Preliminary Insight of Pyrrolylated-Chalcones as New Anti-Methicillin-Resistant Staphylococcus aureus (Anti-MRSA) Agents
Source: Molecules. 2021 Sep 1;26(17):5314. doi: 10.3390/molecules26175314 (PMC8434082; doi:10.3390/molecules26175314)
Supplement: Supplementary file 1 [file molecules-26-05314-s001.zip › molecules-1337951-supplementary.pdf]

SUPPLEMENTARY MATERIALS

Preliminary insight of pyrrolylated-chalcones as new anti-methicillin-resistant Staphylococcus aureus (anti-MRSA) agents

Mohanapriya Gunasekharan[1,a], Tae-Ik Choi[2,a], Yaya Rukayadi[3,4], Muhammad Alif Mohammad Latif[5], Thiruventhan Karunakaran[6], Siti Munirah Mohd Faudzi[1,3,*], Cheol-Hee Kim[2,*]

1 Department of Chemistry, Faculty of Science, Universiti Putra Malaysia, 43400 UPM, Serdang, Selangor, Malaysia.
2 Department of Biology, Chungnam National University, 99 Daehak-ro, Yuseong-gu, Daejeon 34134, Korea
3 Natural Medicines and Product Research Laboratory, Institute of Bioscience, Universiti Putra Malaysia, Serdang, 43400, Selangor, Malaysia
4 Department of Food Science, Faculty of Food Science and Technology, Universiti Putra Malaysia, 43400 UPM, Serdang, Selangor, Malaysia.
5 Centre of Foundation Studies for Agricultural Science, Universiti Putra Malaysia, Serdang, 43400, Selangor, Malaysia.
6 Centre for Drug Research, Universiti Sains Malaysia,11800 USM, Pulau Pinang, Malaysia.
a These authors contributed equally
* Correspondence: sitimunirah@upm.edu.my (S.M.M.F); zebrakim@cnu.ac.kr (C.H.K.)

1. Synthesis

General procedure for the synthesis of 1-4 and 6-15

In a round-bottomed flask, 2 mmol of 2-acetylpyrrole and 2 mmol of substituted benzaldehydes were dissolved in 5 mL of ethanol, prior the dropwise addition of 1 mL of 6 M NaOH after the resulting mixture was stirred for 5 minutes. The reaction mixture was then left to stir overnight at room temperature. The progress of the reaction was monitored through the TLC spotting. Once the reaction is completed, the crushed ice was added to quench the reaction. Following that, the diluted 1 M HCl was added until the reaction mixture reaches pH 7, before further extracted with ethyl acetate (3 × 10 mL). The organic layer was collected, dried over with anhydrous $MgSO_4$ and evaporated under reduced pressure. The crude products (**1-15**) were purified by column chromatography and/or recrystallization technique to yield the pure compounds. The spectroscopic results for new chalconoids (**5**, **11-15**) including the 1D (^{1}H-,^{13}C-, and ^{19}F-NMR) and 2D NMR (HSQC and HMBC), as well as FT-IR, DI-MS and HR-MS were listed in the following sub-section. Meanwhile, the general and spectrometry data, as well as the predicted physicochemical and drug likeness properties of the synthesized molecules **1-15** were tabulated in Table S1 and S2, respectively.

Procedure for the synthesis of 5

10 mL of 6 M NaOH were dissolved in 6 ml water and cooled at 0˚ C in the ice bath, prior the slowly-addition of 10 ml ethanol into the diluted NaOH solution. The reaction flask was then removed from the ice bath and was set at room temperature. Following that, an equimolar quantity of 2-acetylpyrrole and 2-nitrobenzaldehyde were added slowly to the mixture. The reaction mixture was stirred for 2 hr at room temperature prior cooled at 0˚ C for 24 hrs. The precipitates formed were filtered, washed with cold water, and recrystallized with ethanol. The crystals obtained were dried at 70˚ C and kept in room temperature.

Structural characterization of new compounds 5 and 11-15

3-(2-Nitrophenyl)-1-(1H-pyrrol-2-yl) prop-2-en-1-one (**5**)

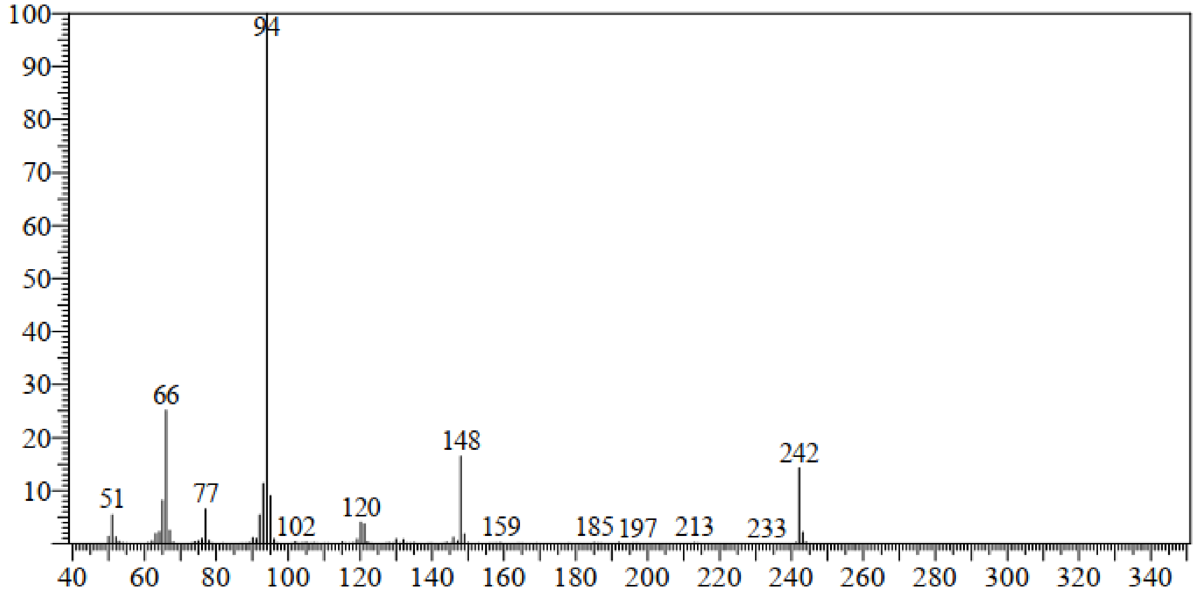

DI-MS m/z 242 [M$^+$]

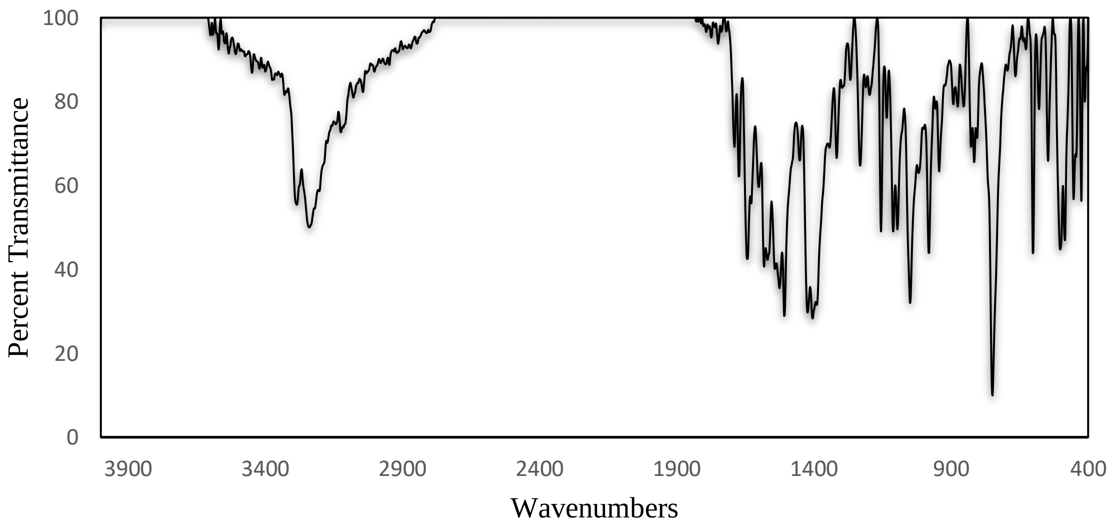

FT-IR

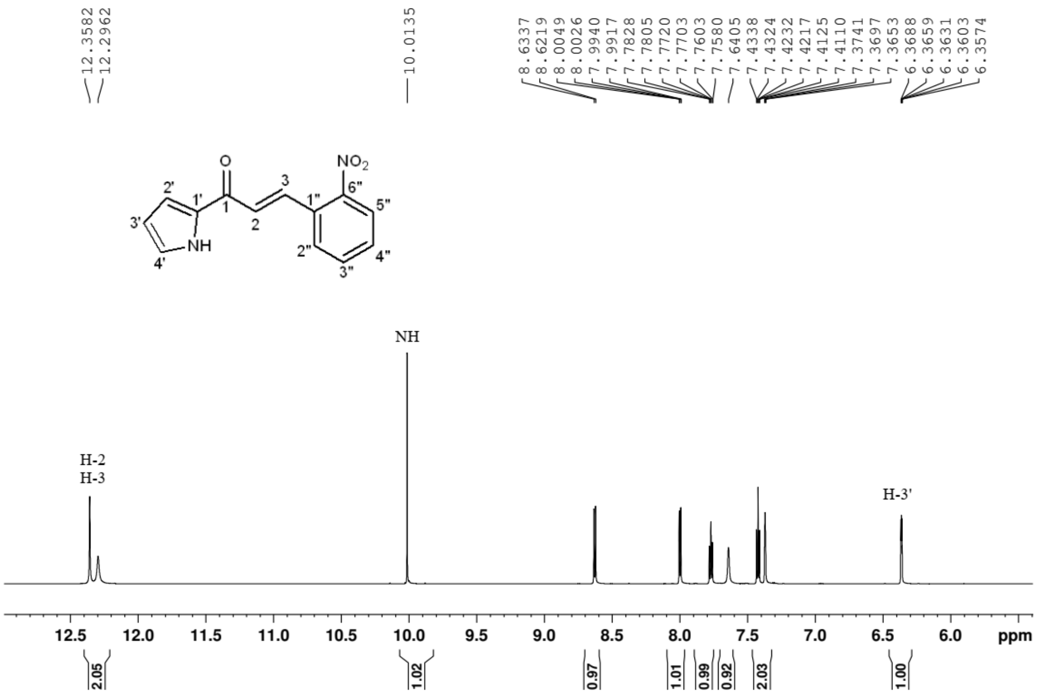

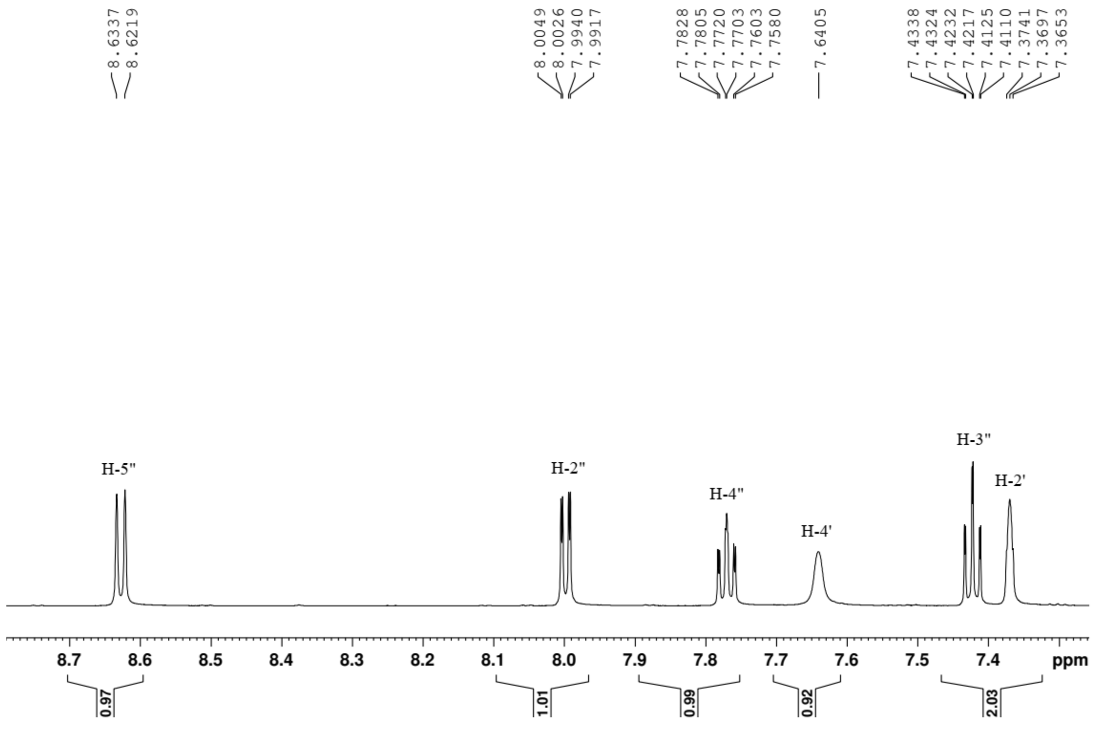

^{1}H-NMR (expansion)

^{1}H-NMR (700 MHz, DMSO-d$_6$) δ: 6.37 (*m*, 1H, Py-H), 7.37 (*t*, *J*=3.1 Hz, 1H, Py-H), 7.42 (*td*, *J*=1.1 & 7.4 Hz, 1H, Ar-H), 7.64 (*s*, 1H, Py-H), 7.77 (*td*, *J*=1.2 & 7.6 Hz, 1H, Ar-H), 8.00 (*dd*, *J*=1.6 & 7.6 Hz, 1H, Ar-H), 8.63 (*d*, *J*=8.3 Hz, 1H, Ar-H), 10.0 (*s*, 1H, NH), 12.30 (*br. s*, 2H, CH=CH);

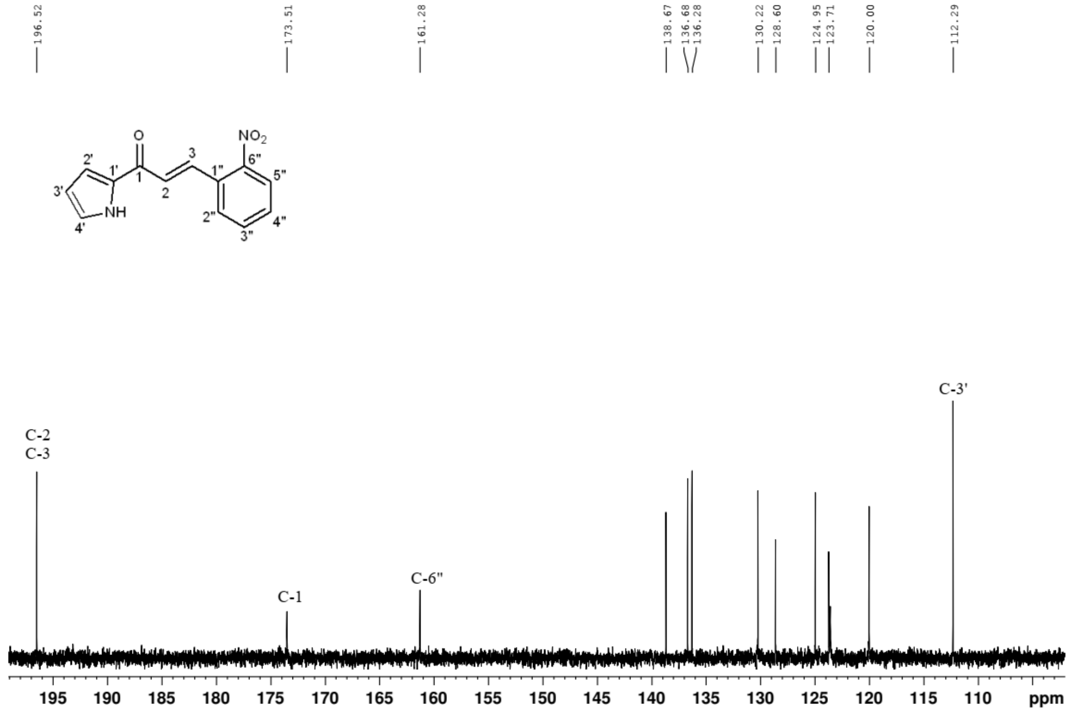

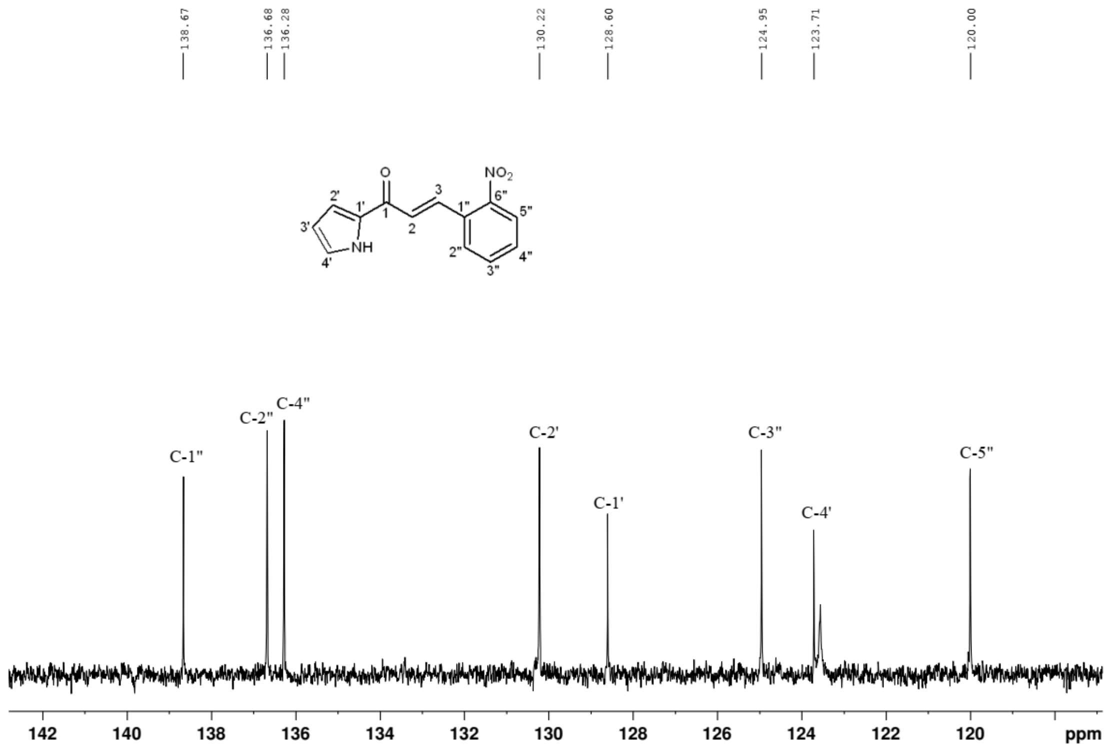

^{13}C-NMR (expansion)

^{13}C-NMR (175 MHz, DMSO-d$_6$) δ: 112.2, 120.0, 123.7, 124.9, 128.6, 130.2, 136.2, 136.6, 138.6, 161.2, 173.5, 196.5

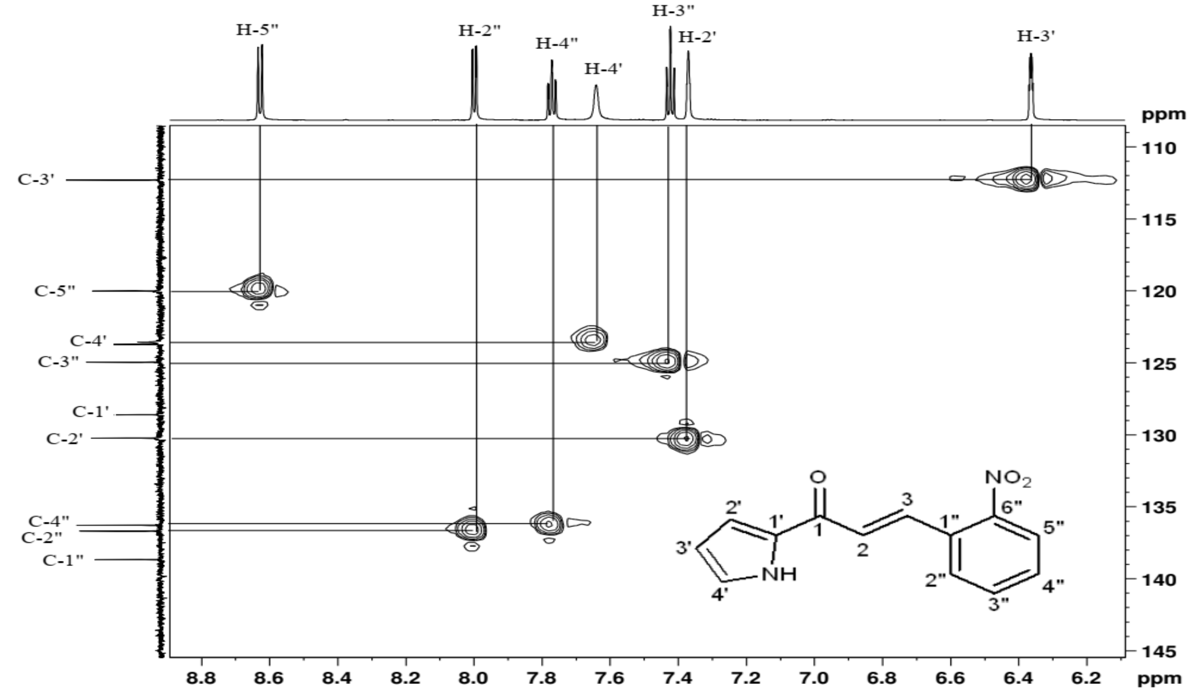

HSQC

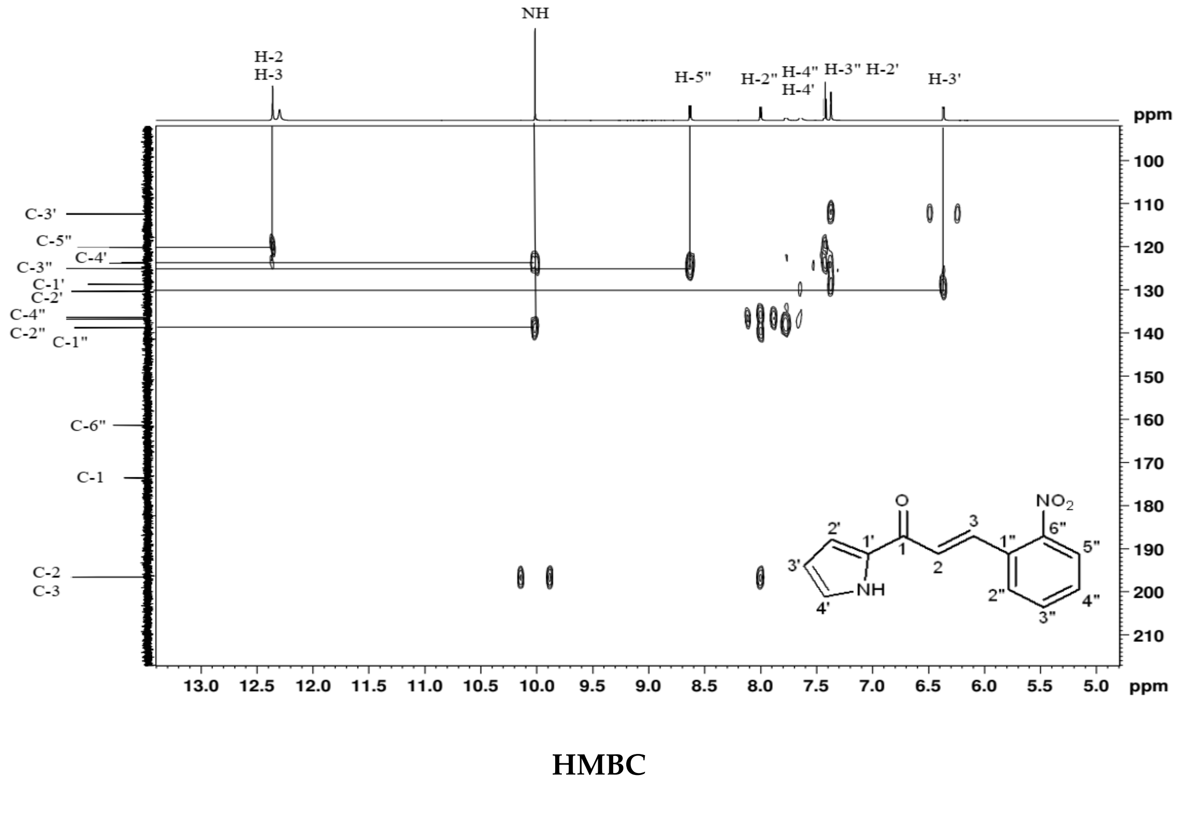

HMBC

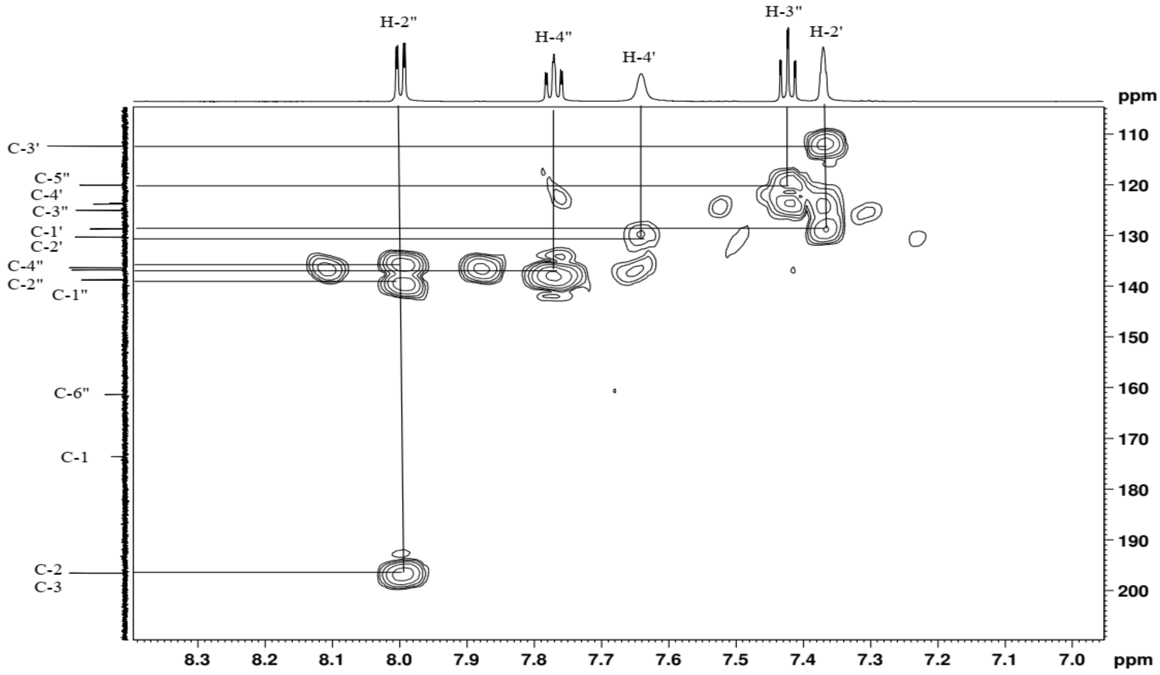

HMBC (expansion)

*3-(2, 3-Difluorophenyl)-1-(1H-pyrrol-2-yl) prop-2-en-1-one (**11**)*

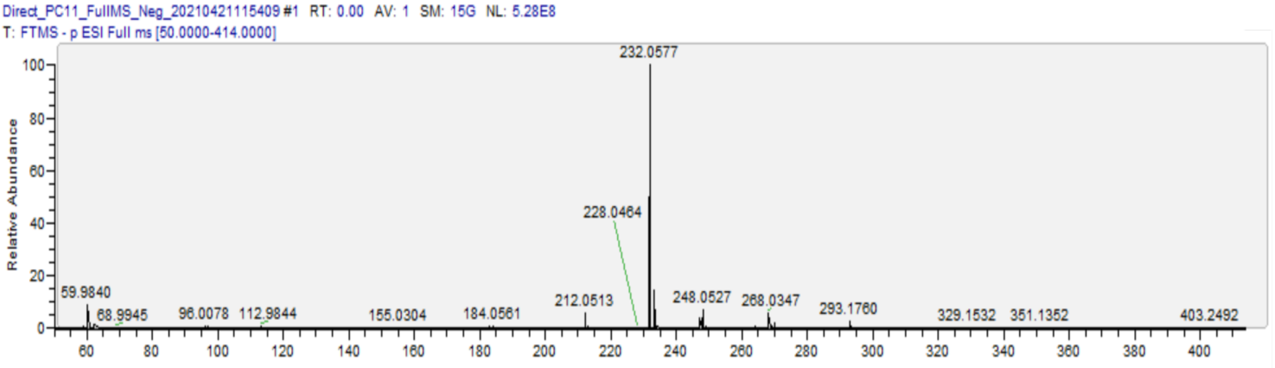

HRMS (ESI) [M-H]$^{-}$: found: 232.0577

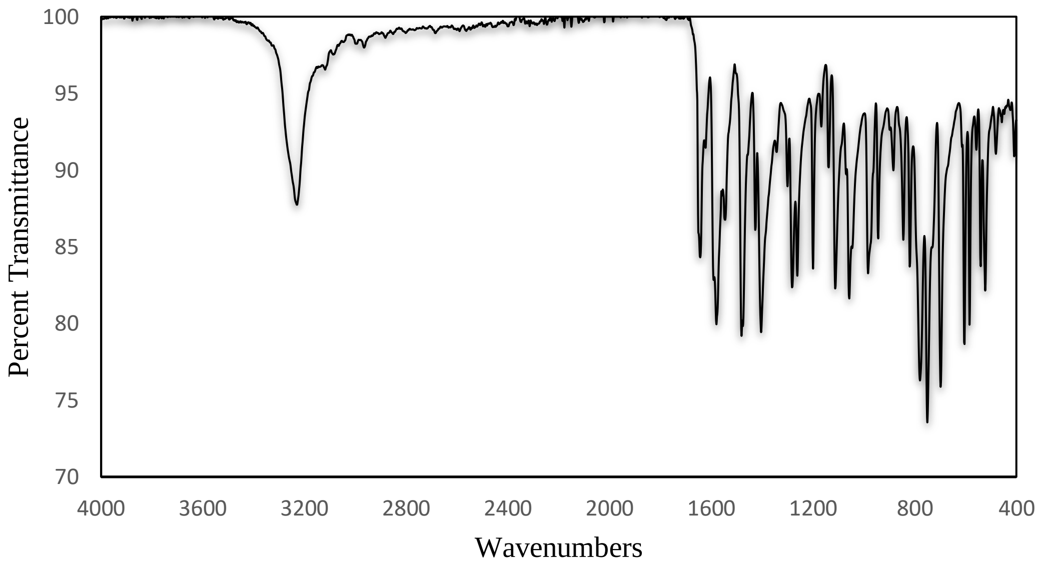

FT-IR

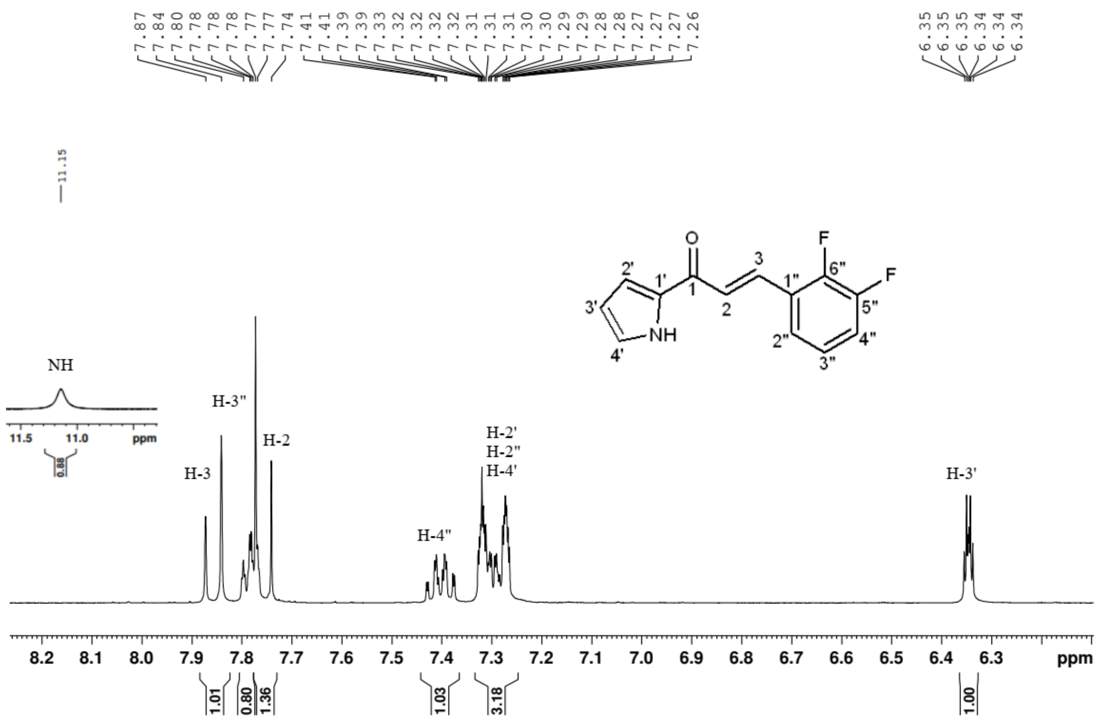

^{1}**H-NMR** (500 MHz, acetone-d6) δ : 6.34 (m, 1H, Py-H), 7.27 (m, 1H, Py-H), 7.30 (m, 1H, Ar-H), 7.32 (m, 1H, Py-H), 7.40 (m, 1H, Ar-H), 7.75 (d, J=15.9 Hz, 1H, CH=CH), 7.78 (dd, J=1.5 & 6.5 Hz, 1H, Ar-H), 7.85 (d, J=15.8 , 1H, CH=CH), 11.15 (br. s, 1H, NH)

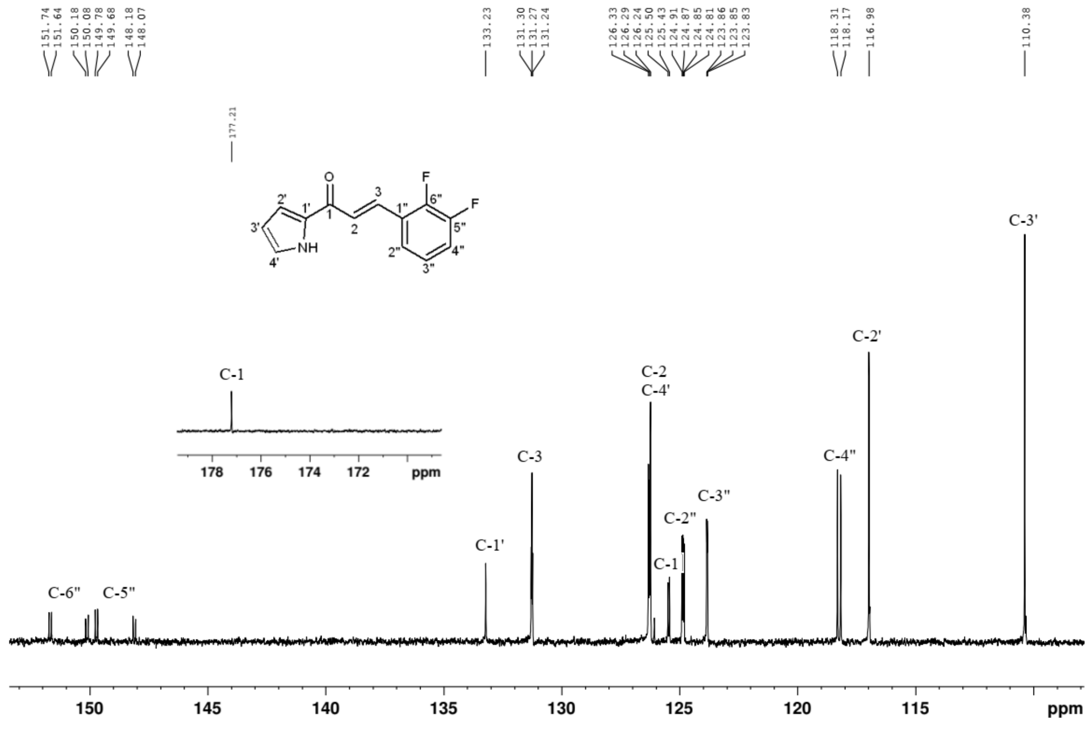

^{13}**C-NMR** (125 MHz, acetone-d$_6$) δ: 110.3, 116.9, 118.2, 123.8, 124.8, 125.4, 126.2, 131.2, 133.2, 148.9, 150.7, 177.2

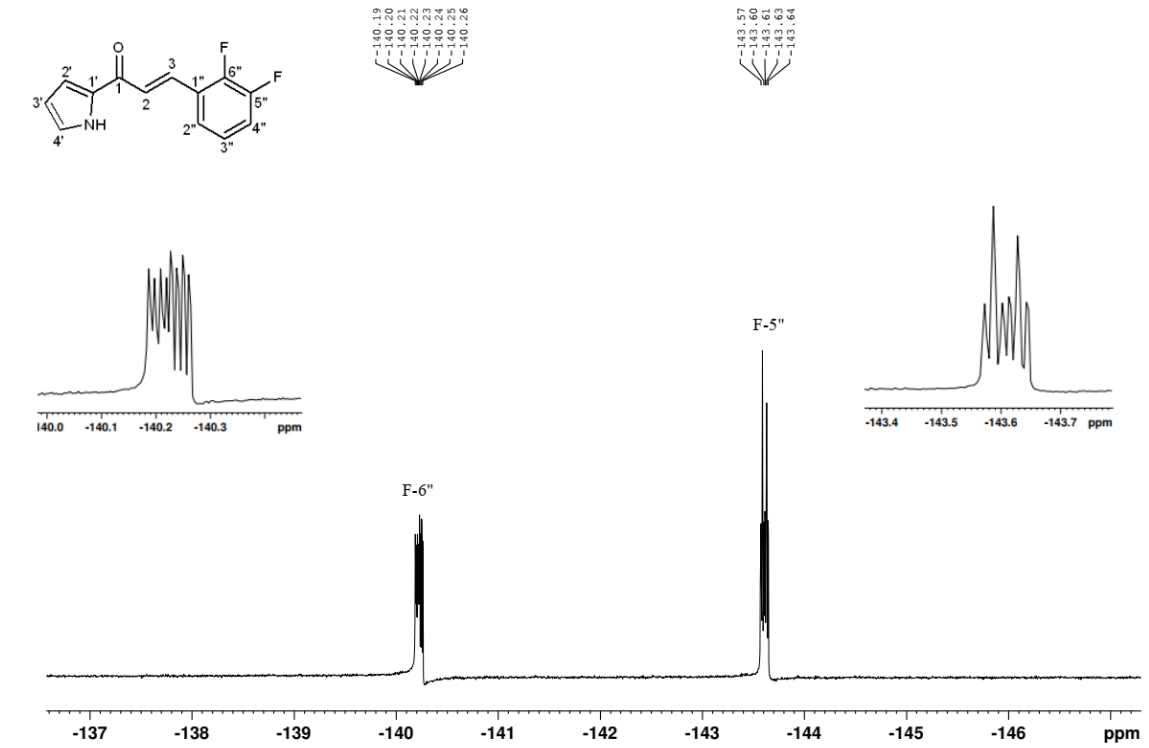

^{19}**F-NMR** (470 MHz, acetone-d_6) δ: -140.2 & -143.6

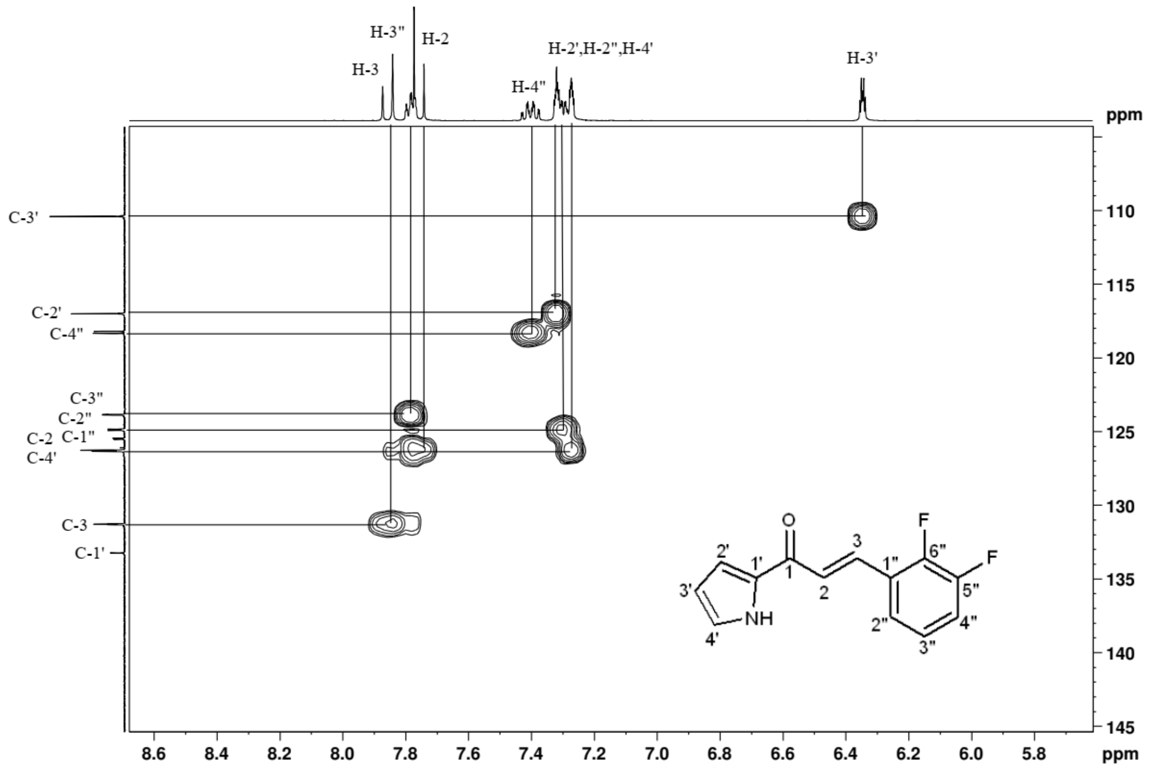

HSQC

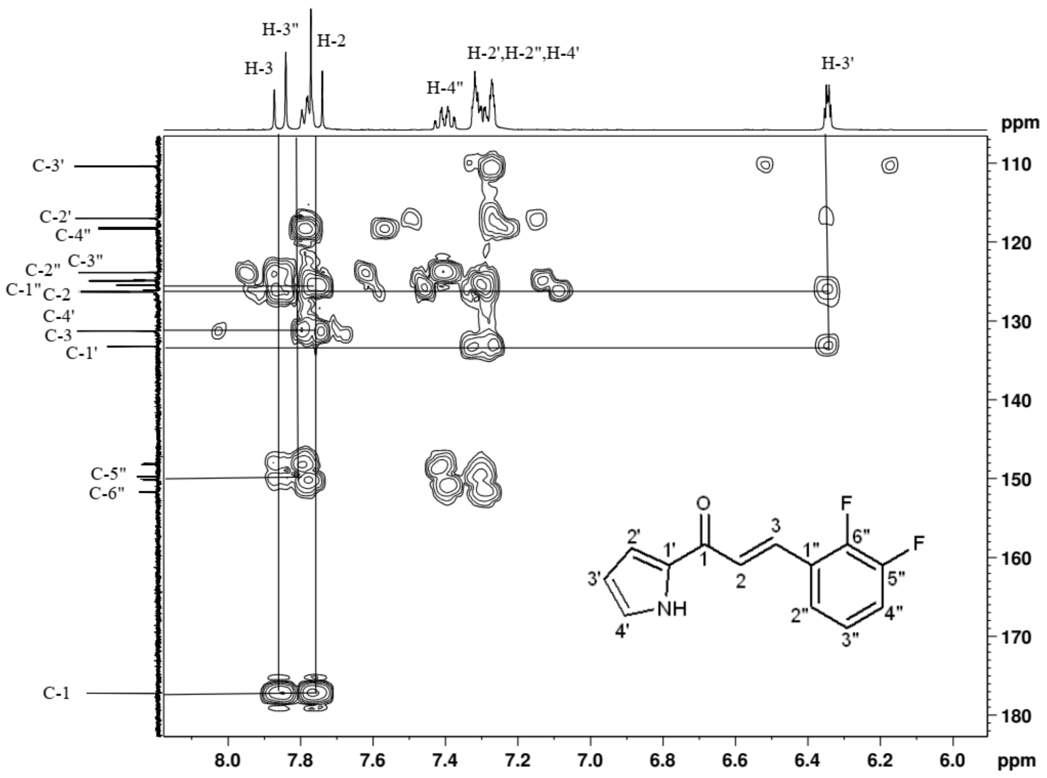

HMBC

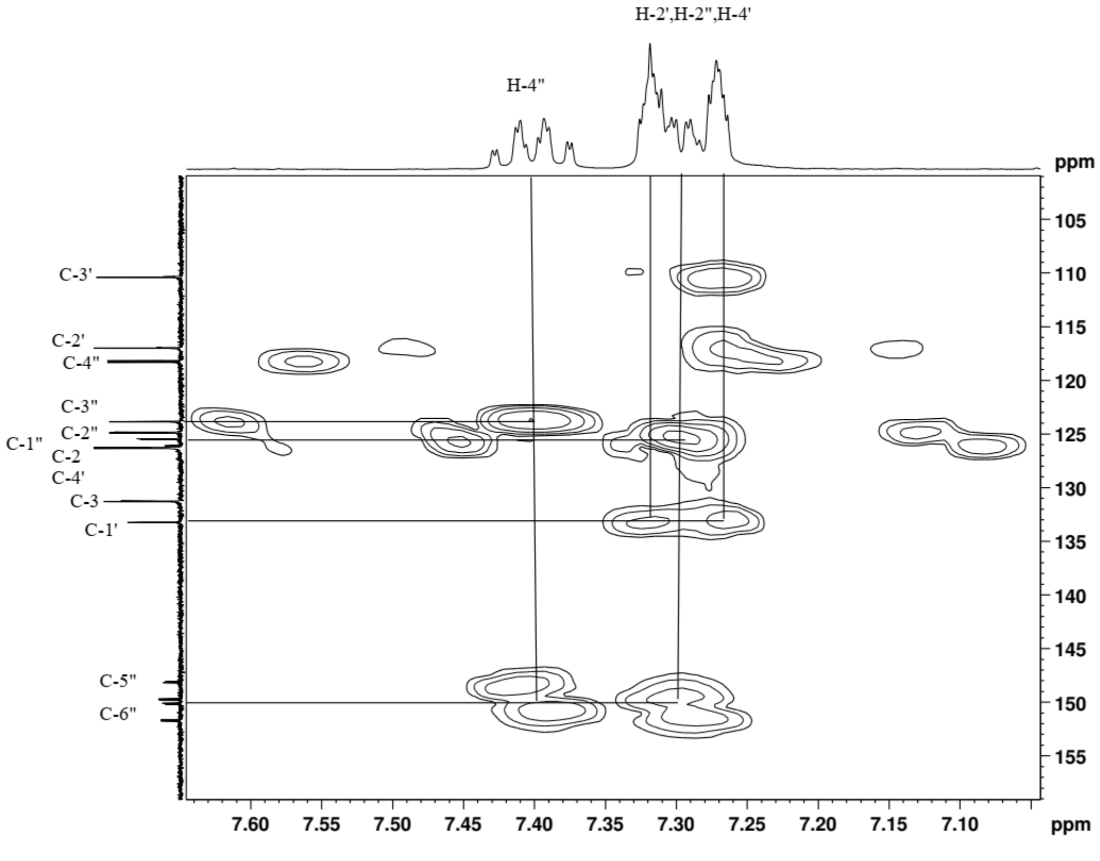

HMBC (expansion)

*3-(2, 4-Difluorophenyl)-1-(1H-pyrrol-2-yl) prop-2-en-1-one (**12**)*

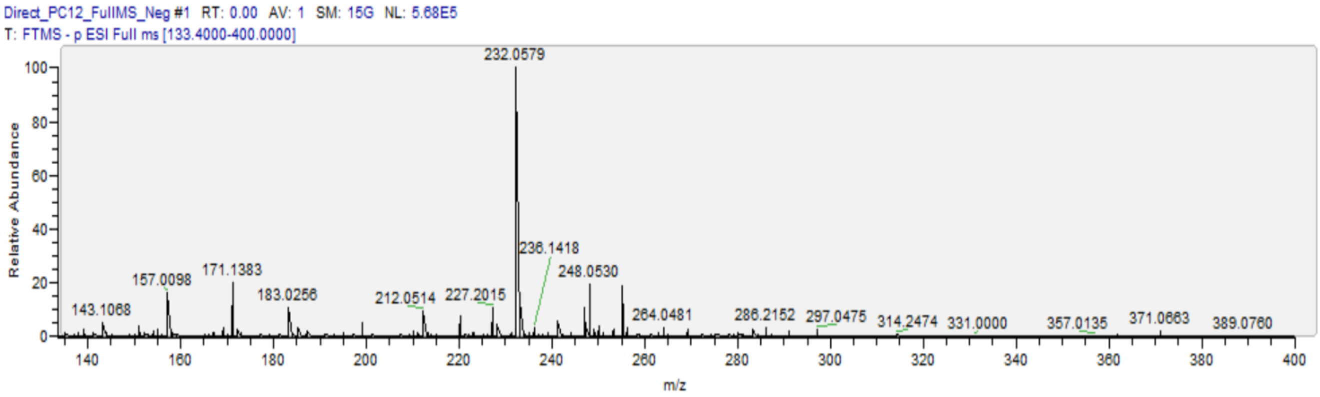

HRMS (ESI) [M-H]$^-$: found: 232.0579

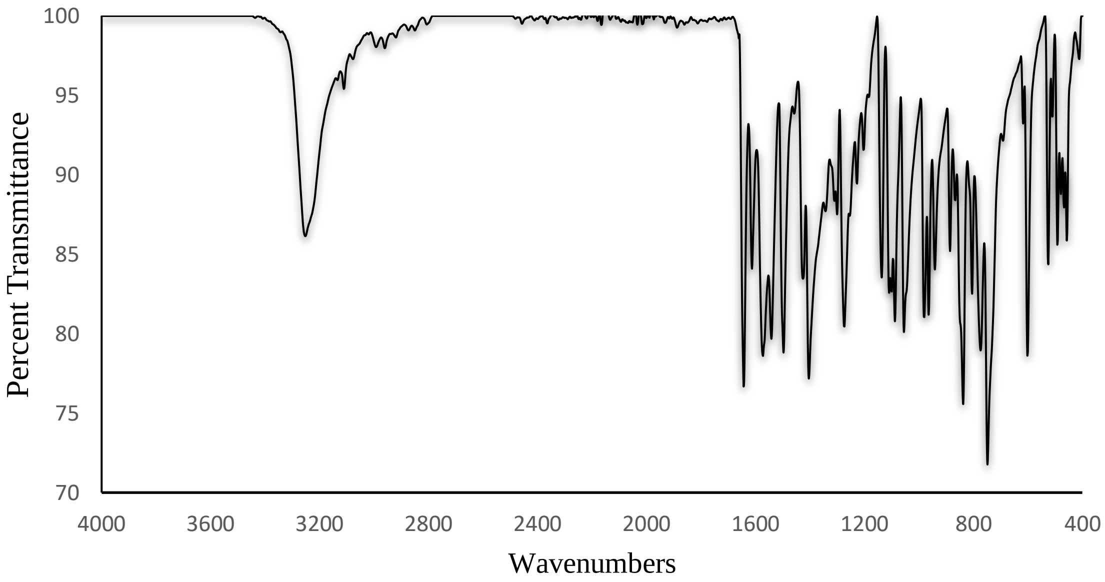

FT-IR

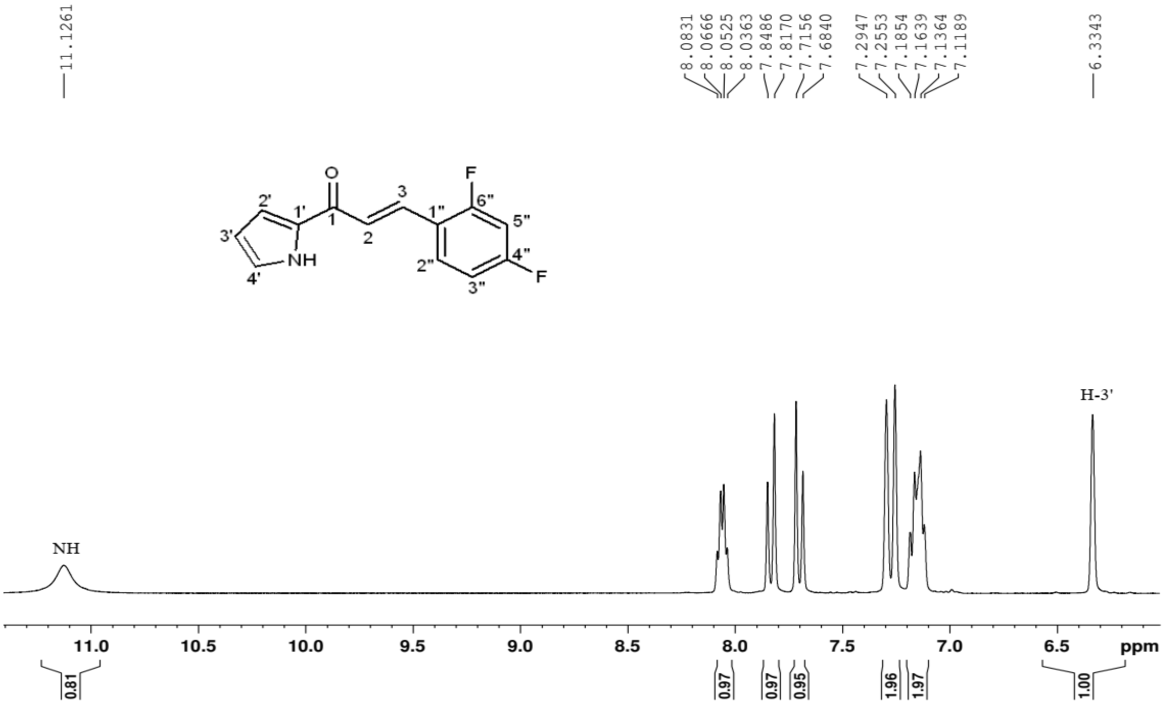

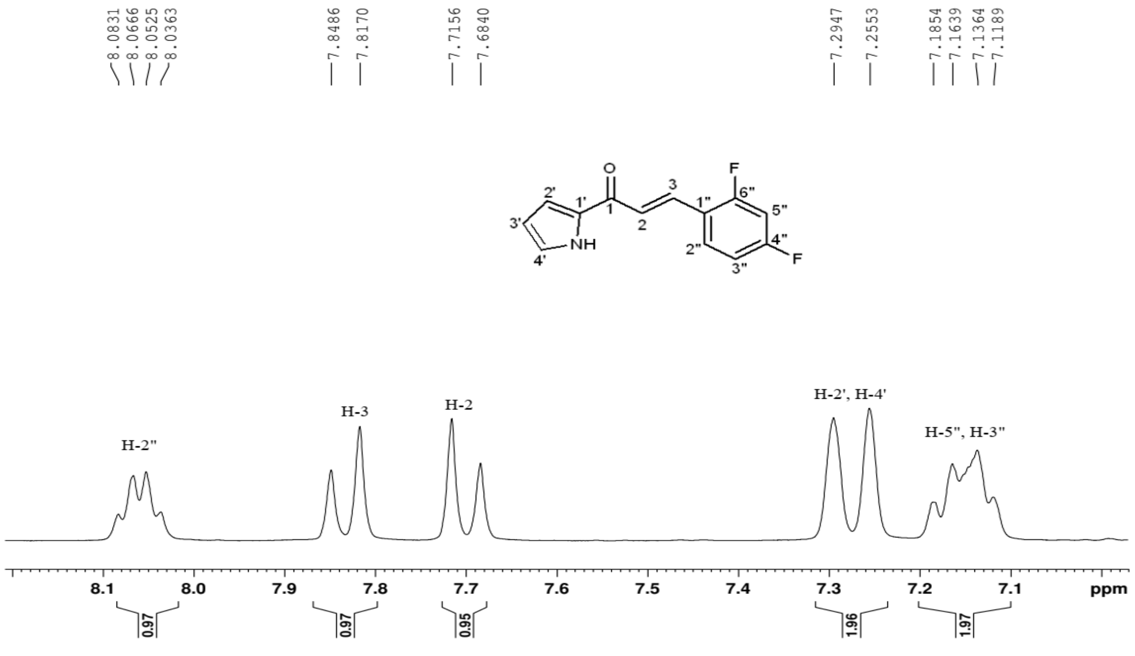

¹H NMR (expansion)

¹H NMR (500 MHz, acetone-d_6) δ : 6.33 (*br. s*, 1H, Py-H), 7.15 (*m*, 2H, Ar-H), 7.26 (*br. s*, 1H, Py-H), 7.29 (*br. s*, 1H, Py-H), 7.70 (*d*, *J*=15.8, 1H, CH=CH), 7.83 (*d*, *J*=15.8, 1H, CH=CH), 8.06 (*dd*, *J*=7.1 & 8.3 Hz 1H, Ar-H), 11.13 (*br. s*, 1H, NH)

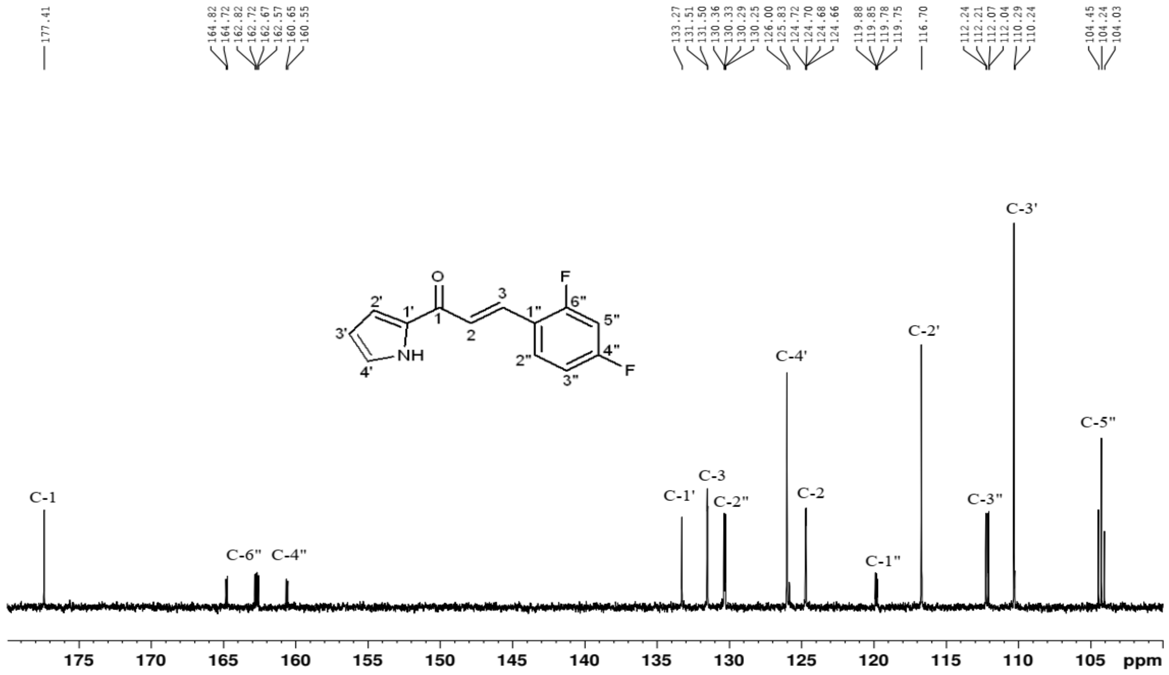

^{13}C NMR (125 MHz, acetone-d$_6$) δ : 104.2, 110.2, 112.1, 116.7, 119.8, 124.6, 125.9, 130.3, 131.5, 133.2, 161.6, 163.8, 177.4

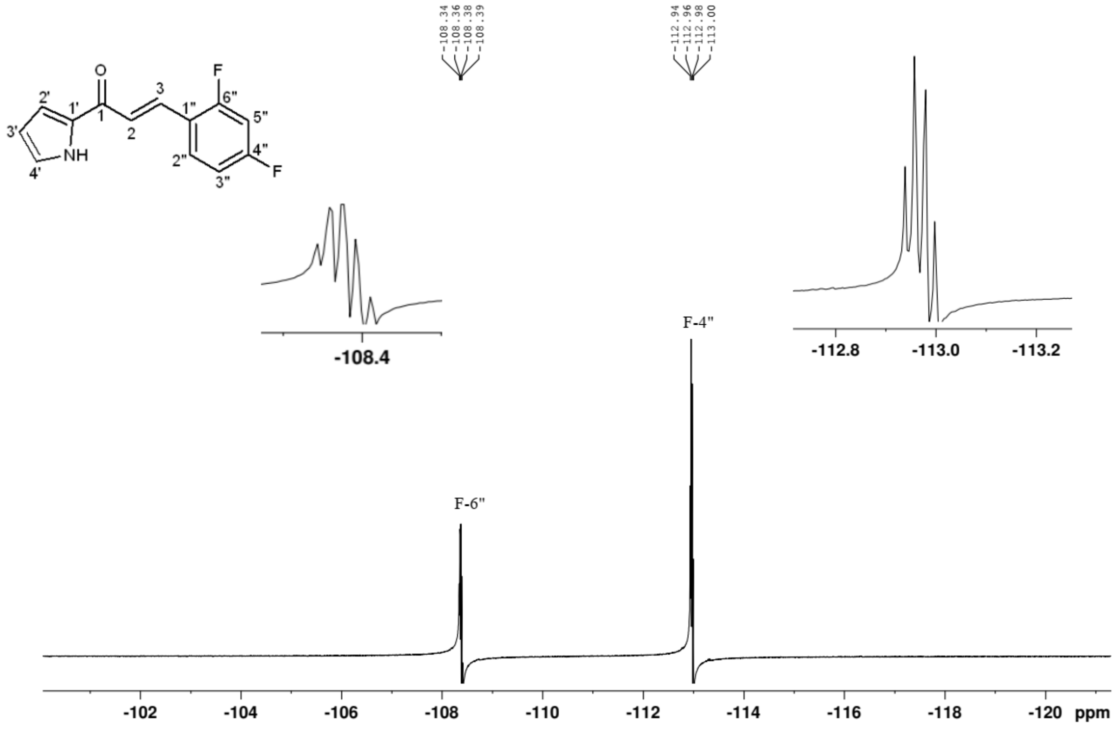

^{19}F NMR (470 MHz, acetone-d$_6$) δ: -108.3 & -112.9

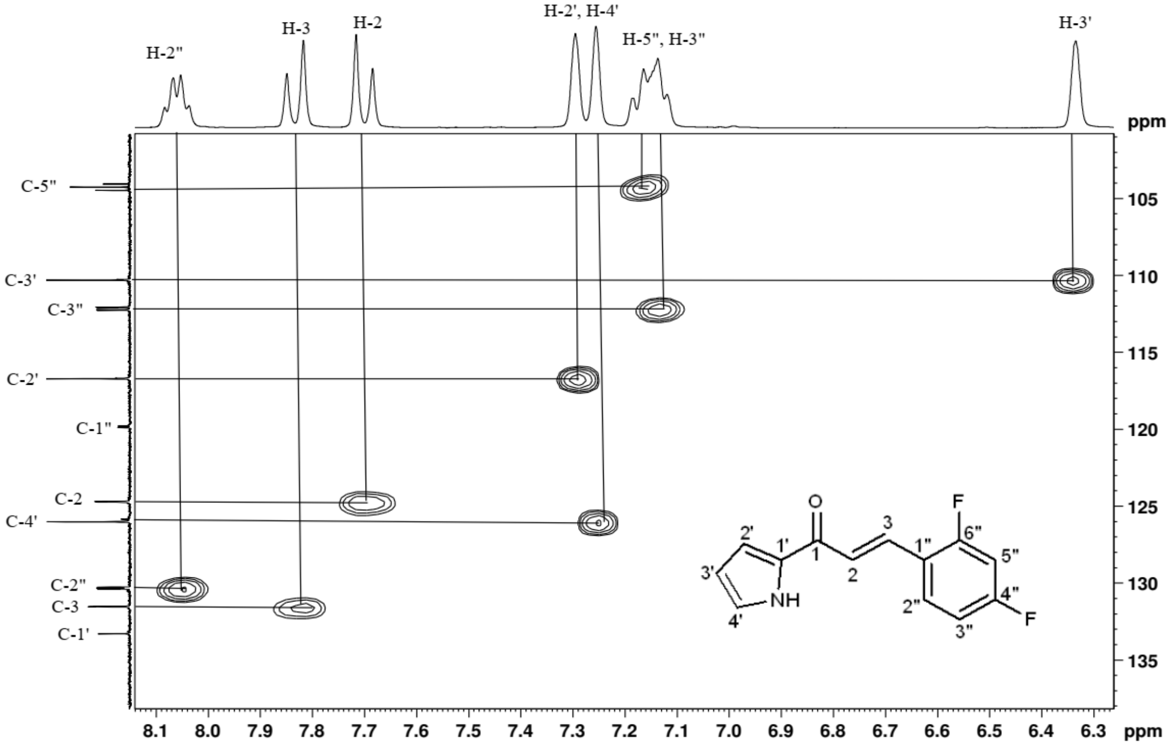
H-2"
H-3
H-2
H-2', H-4'
H-5", H-3"
H-3'
ppm
C-5"
C-3'
C-3"
C-2'
C-1"
C-2
C-4'
C-2"
C-3
C-1'
105
110
115
120
125
130
135
ppm
8.1 8.0 7.9 7.8 7.7 7.6 7.5 7.4 7.3 7.2 7.1 7.0 6.9 6.8 6.7 6.6 6.5 6.4 6.3
ppm
O
F
NH
1
2
3
1'
2'
3'
4'
1"
2"
3"
4"
5"
6"

HSQC

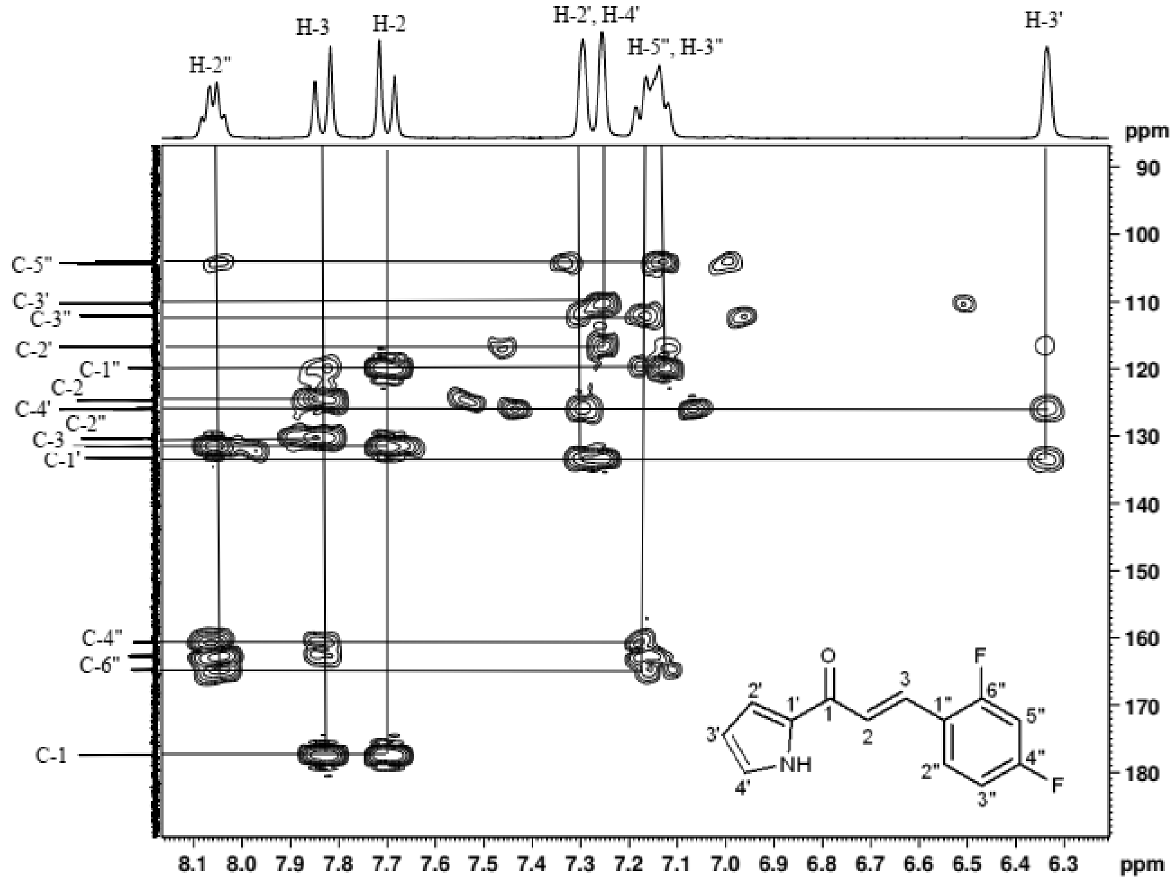
H-2"
H-3
H-2
H-2', H-4'
H-5", H-3"
H-3'
ppm
C-5"
C-3'
C-3"
C-2'
C-1"
C-2
C-4'
C-2"
C-3
C-1'
C-4"
C-6"
C-1
90
100
110
120
130
140
150
160
170
180
ppm
8.1 8.0 7.9 7.8 7.7 7.6 7.5 7.4 7.3 7.2 7.1 7.0 6.9 6.8 6.7 6.6 6.5 6.4 6.3
ppm
O
F
NH
1
2
3
1'
2'
3'
4'
1"
2"
3"
4"
5"
6"

HMBC

*3-(2, 5-Difluorophenyl)-1-(1H-pyrrol-2-yl) prop-2-en-1-one (**13**)*

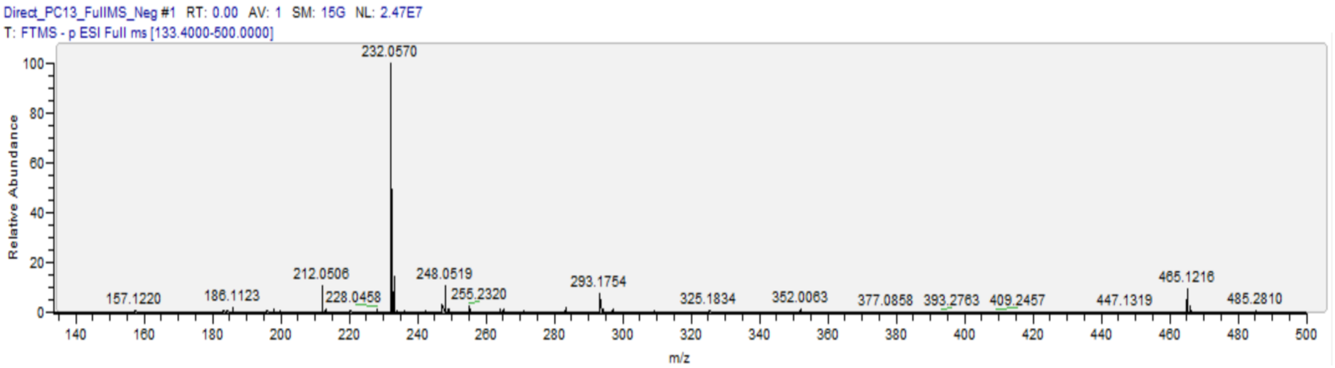

HRMS (ESI) [M-H]$^-$: found: 232.0570

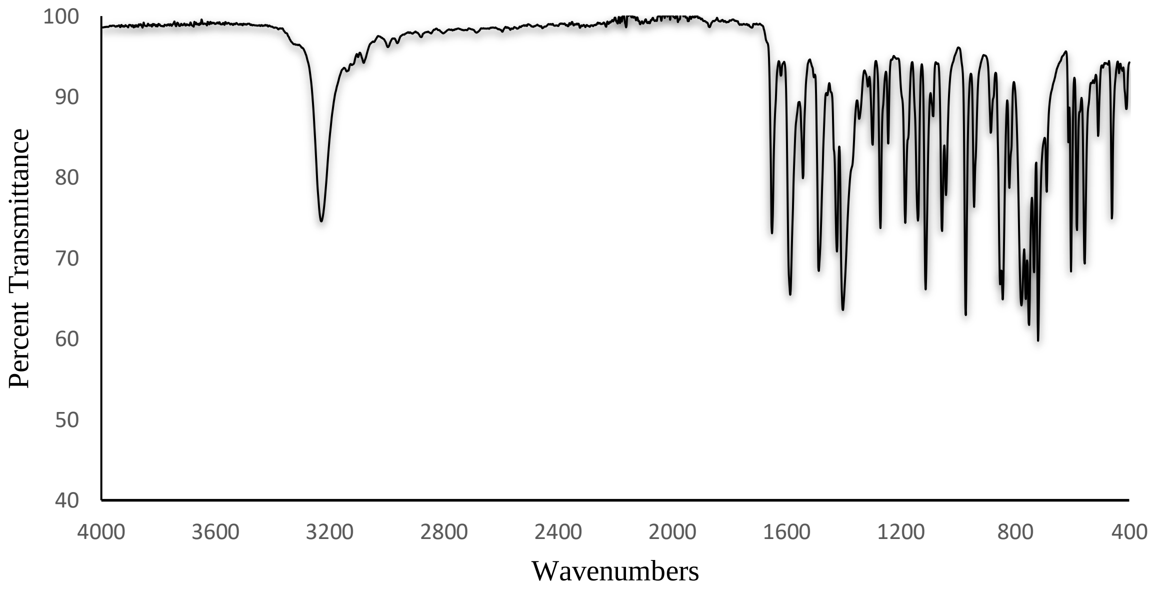

FT-IR

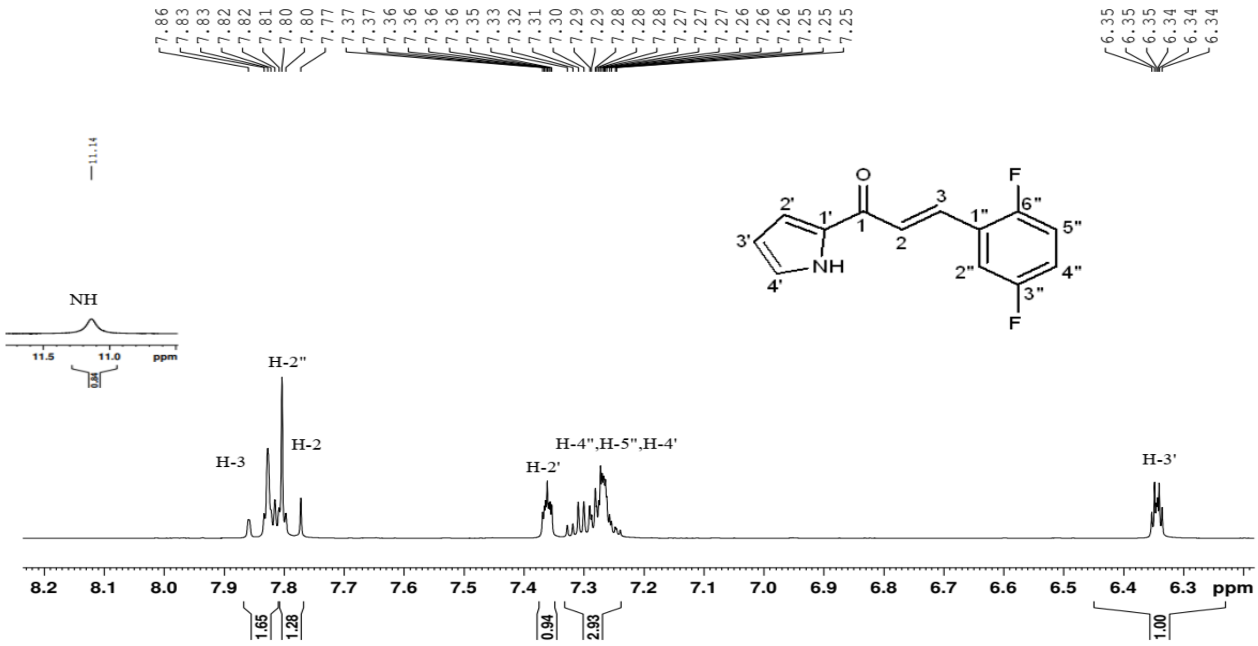

[1]**H NMR** (500 MHz, acetone-d$_6$) δ : 6.34 (*m*, 1H, Py-H), 7.28 (*m*, 1H, Py-H), 7.31 (*dd*, *J*=4.7 & 9.2 Hz, 2H, Ar-H), 7.36 (*m*, 1H, Py-H), 7.78 (*d*, *J*=15.8, 1H, CH=CH), 7.81 (*m*, 1H, Py-H), 7.84 (*d*, *J*=16.1, 1H, CH=CH), 11.14 (*br*. *s*, 1H, NH)

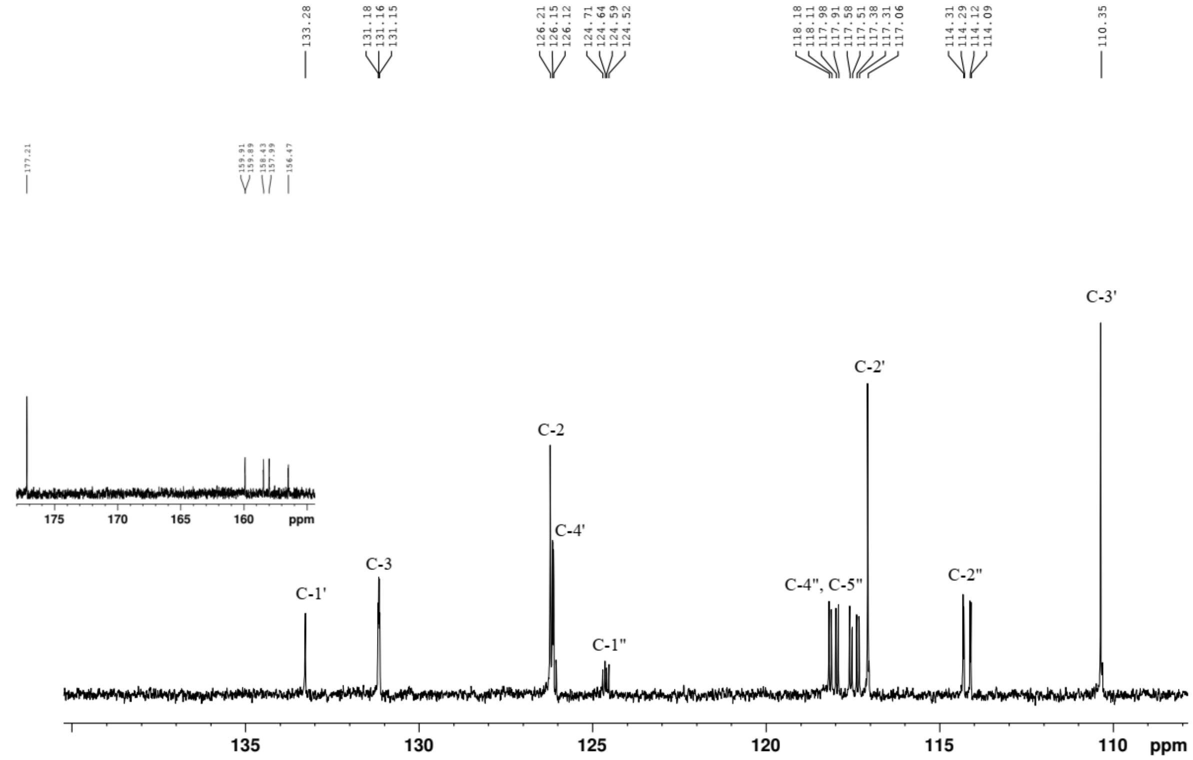

[13]**C NMR** (125 MHz, acetone-d$_6$) δ: 110.3, 114.2, 117.0, 117.5, 118.1, 124.6, 126.1, 126.2, 131.1, 133.2, 157.2, 159.2, 177.2

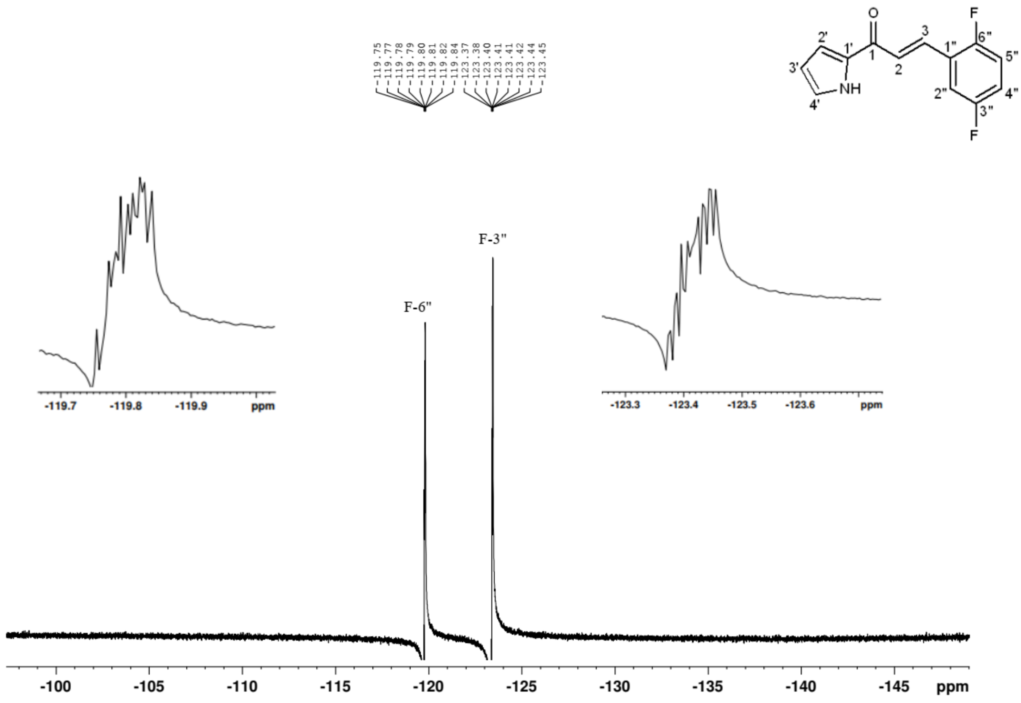

¹⁹F NMR (470 MHz, acetone-d_6) δ: -119.8 & -123.4

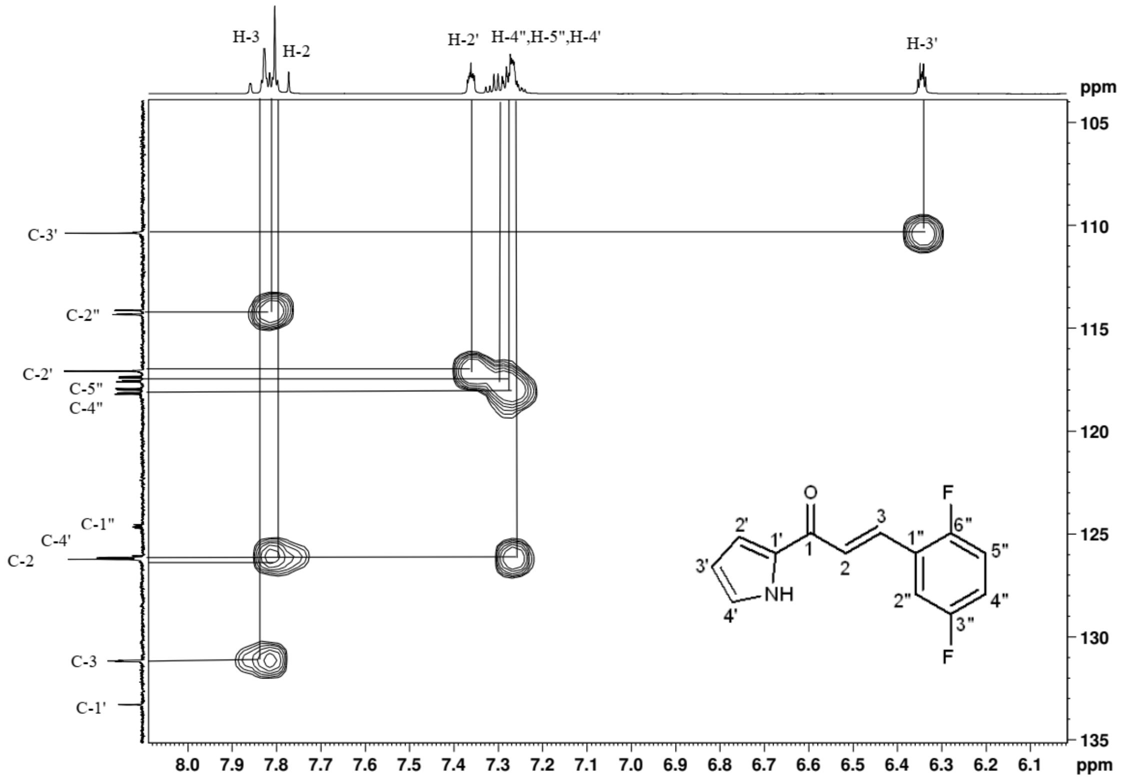

HSQC

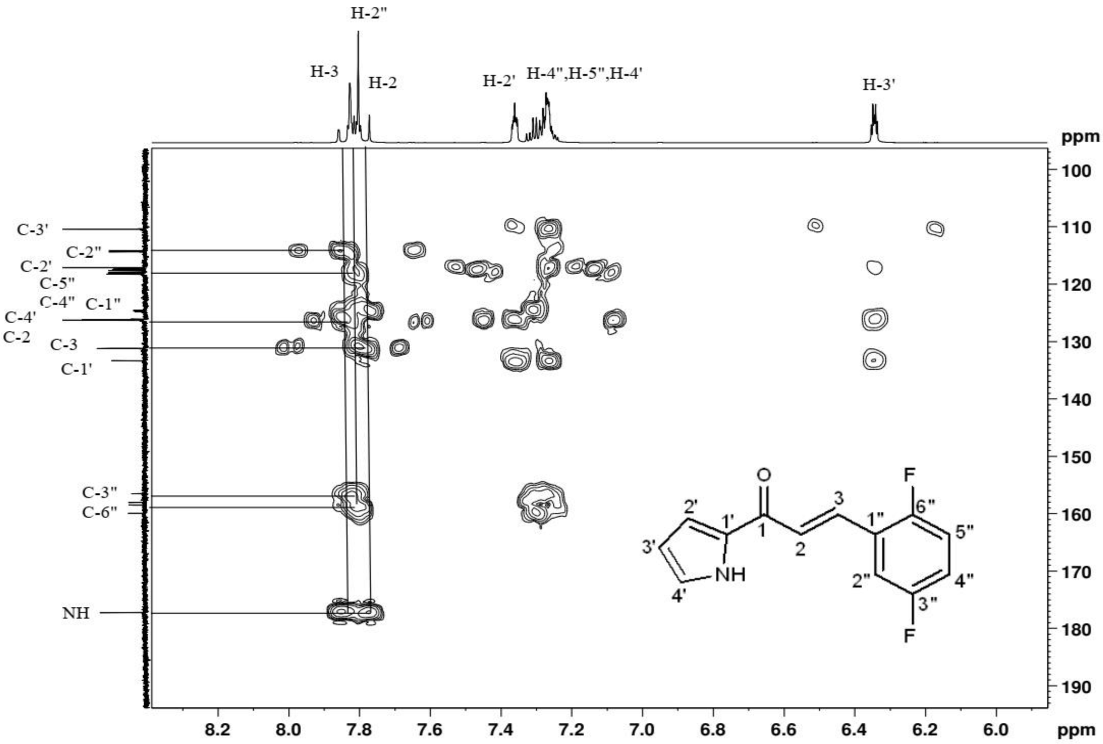

HMBC

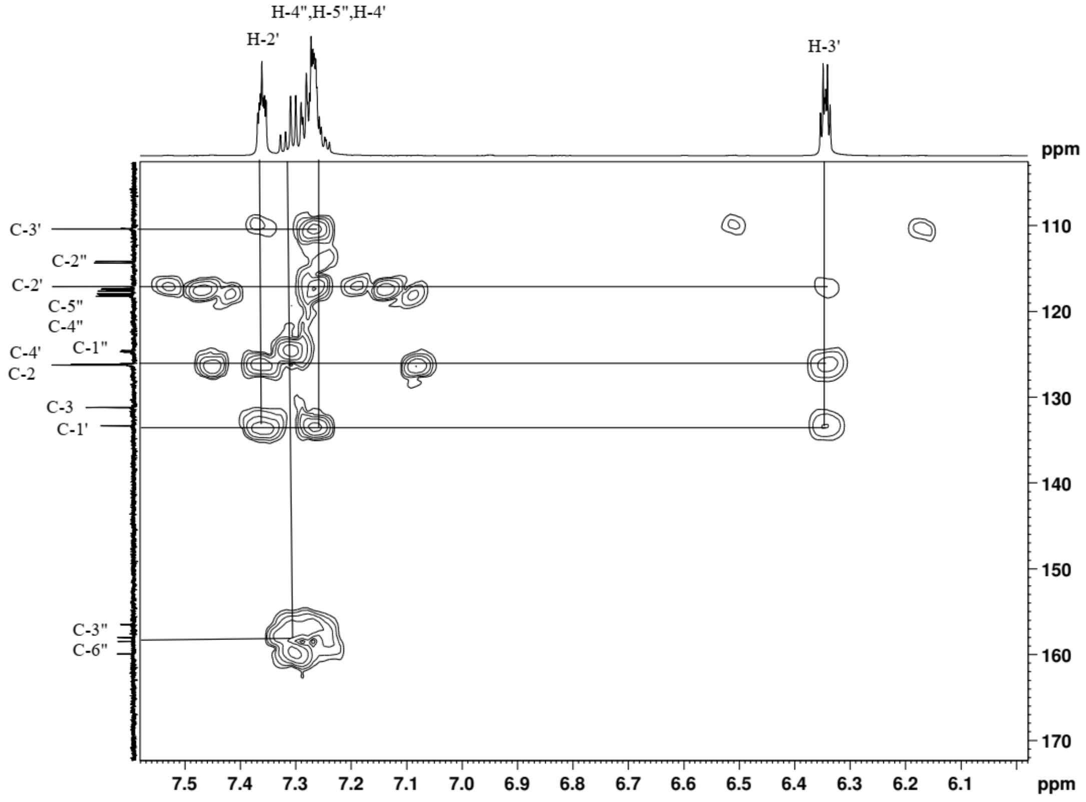

HMBC (expansion)

*3-(3, 4-Difluorophenyl)-1-(1H-pyrrol-2-yl) prop-2-en-1-one (**14**)*

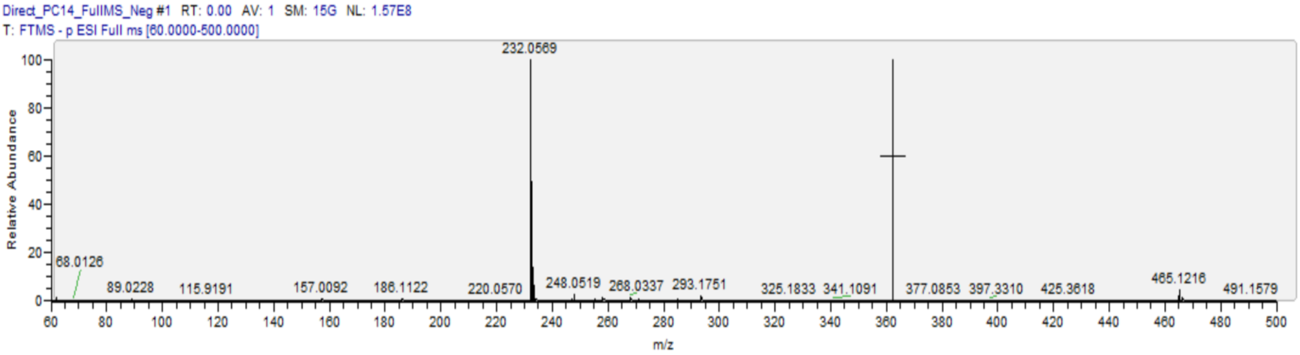

HRMS (ESI) [M-H]⁻ : found: 232.0569

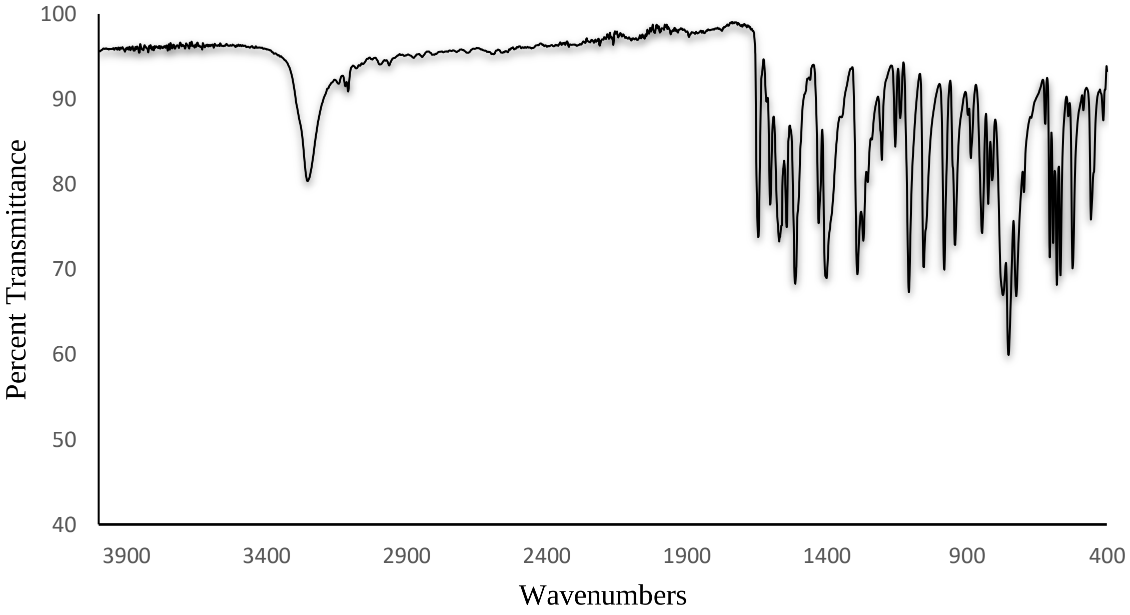

FT-IR

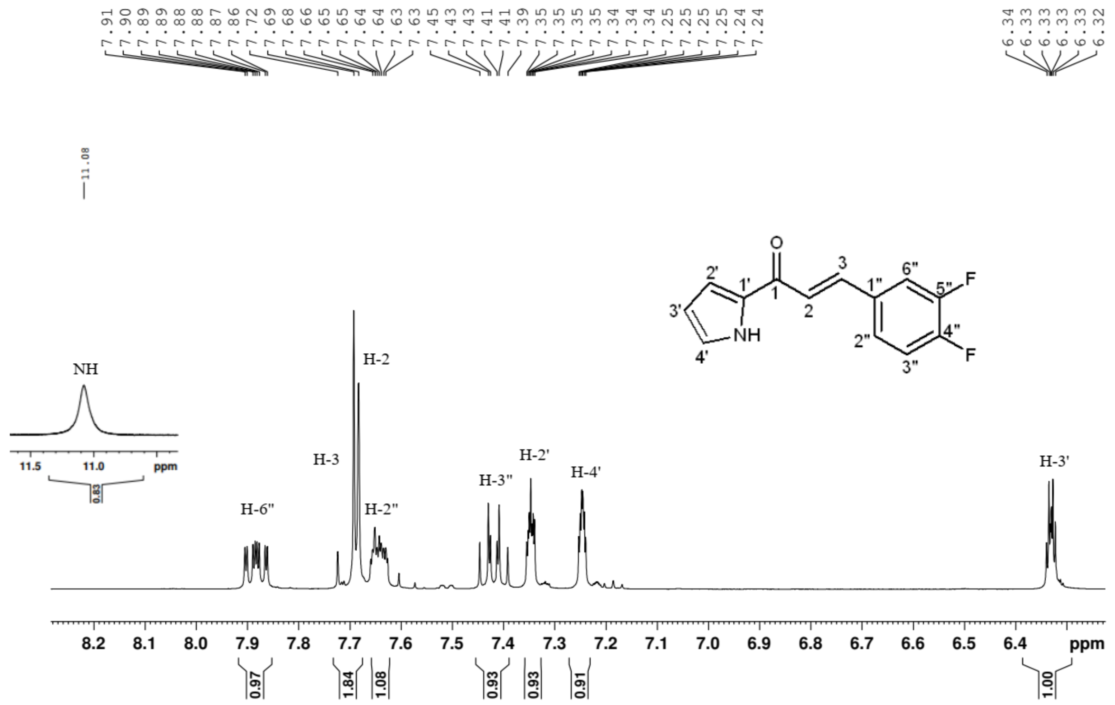

^{1}**H NMR** (500 MHz, acetone-d$_6$) δ: 6.33 (*m*, 1H , Py-H), 7.24 (*m*, 1H, Py-H), 7.34 (*m*, 1H, Py-H), 7.42 (*m*, 1H, Ar-H), 7.64 (*m*, 1H, Ar-H), 7.66 (*d*, *J*=16.1, 1H, CH=CH), 7.70 (*d*, *J*=15.7, 1H, CH=CH), 7.88 (*m*, 1H, Ar-H), 11.08 (*br. s*, 1H, NH)

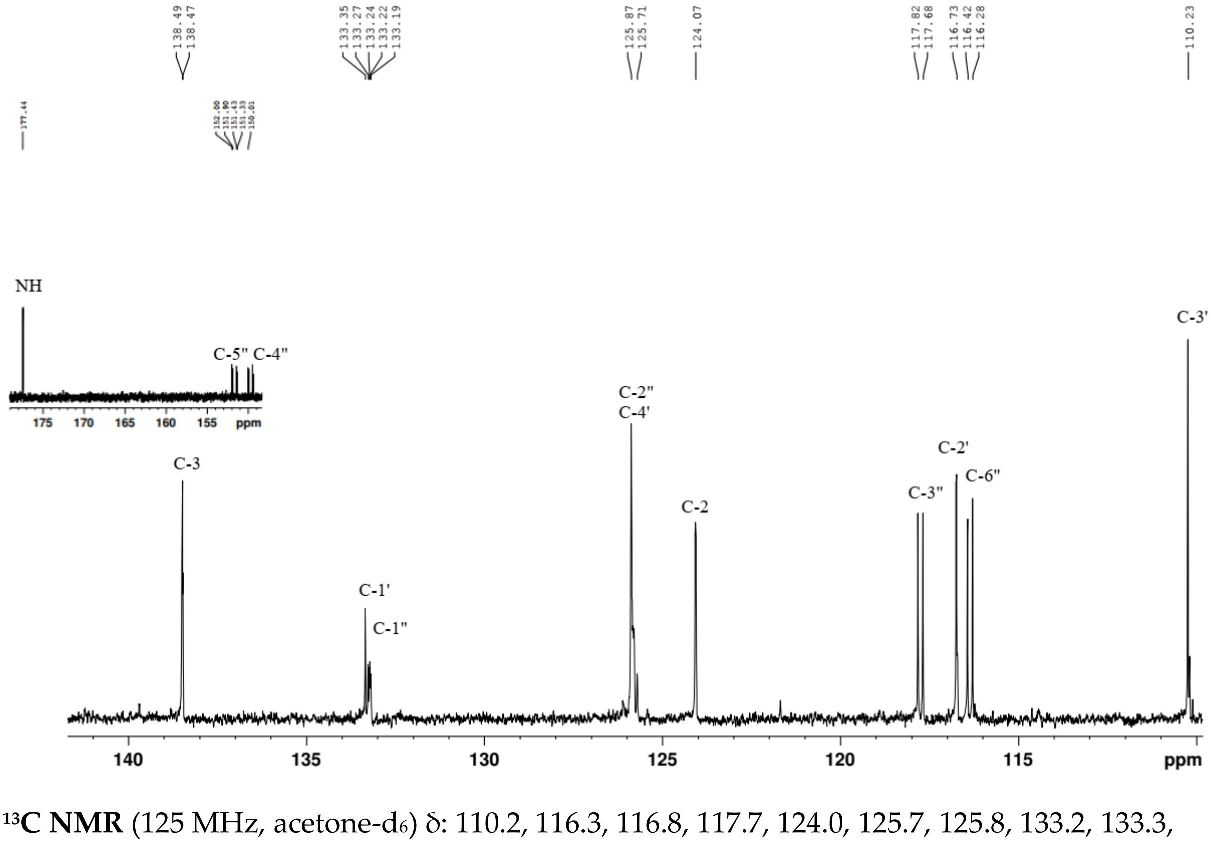

^{13}**C NMR** (125 MHz, acetone-d$_6$) δ: 110.2, 116.3, 116.8, 117.7, 124.0, 125.7, 125.8, 133.2, 133.3, 138.4, 149.7, 151.7, 177.4

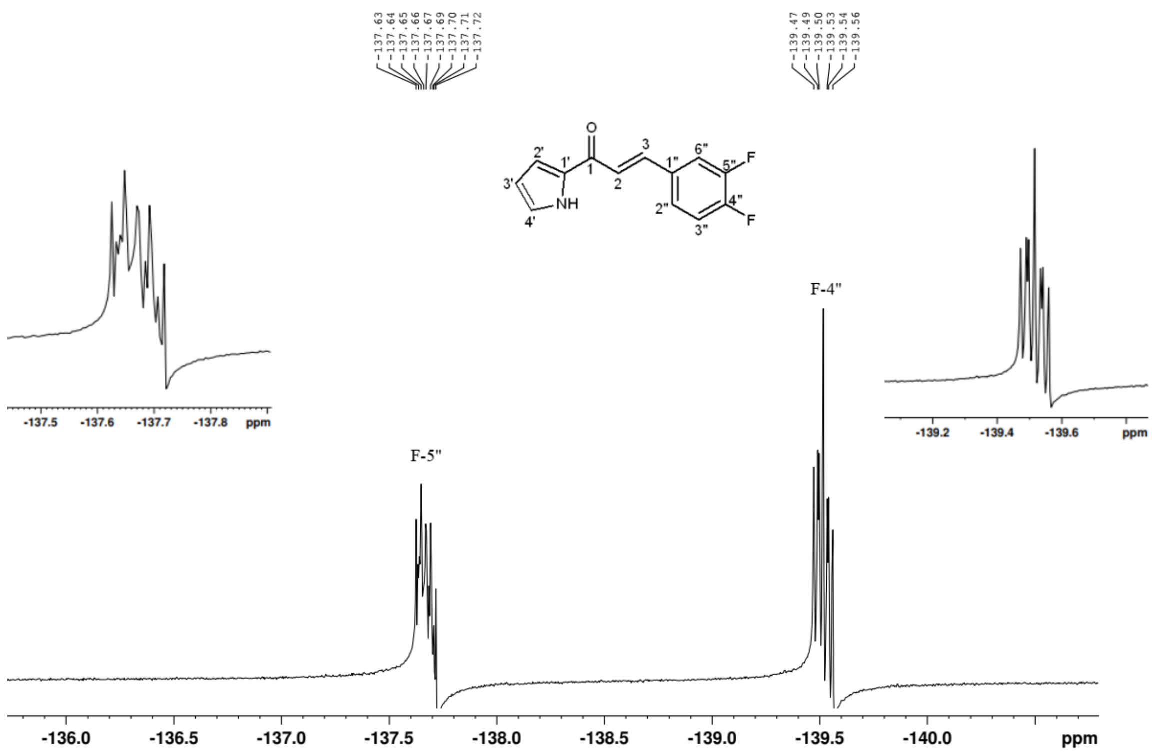

^{19}F NMR (470 MHz, acetone-d$_6$) δ: -137.6 & -139.5

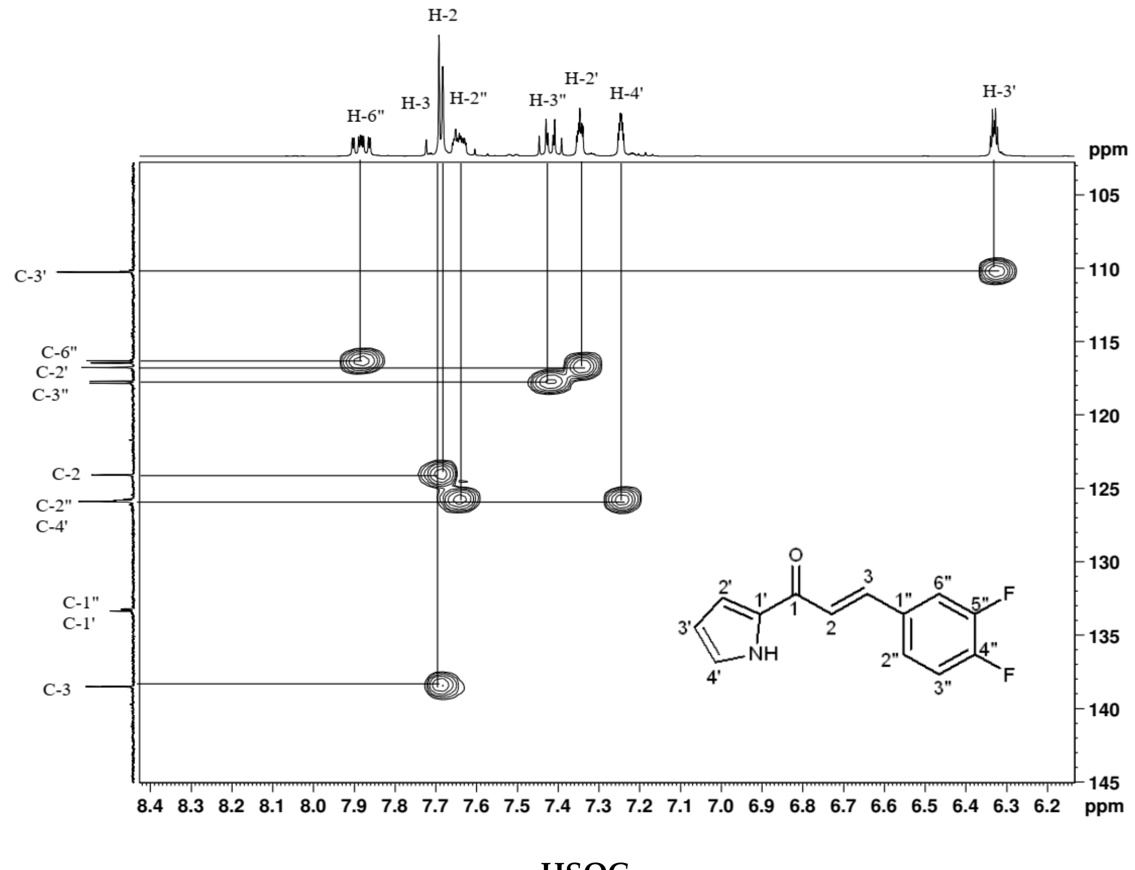

HSQC

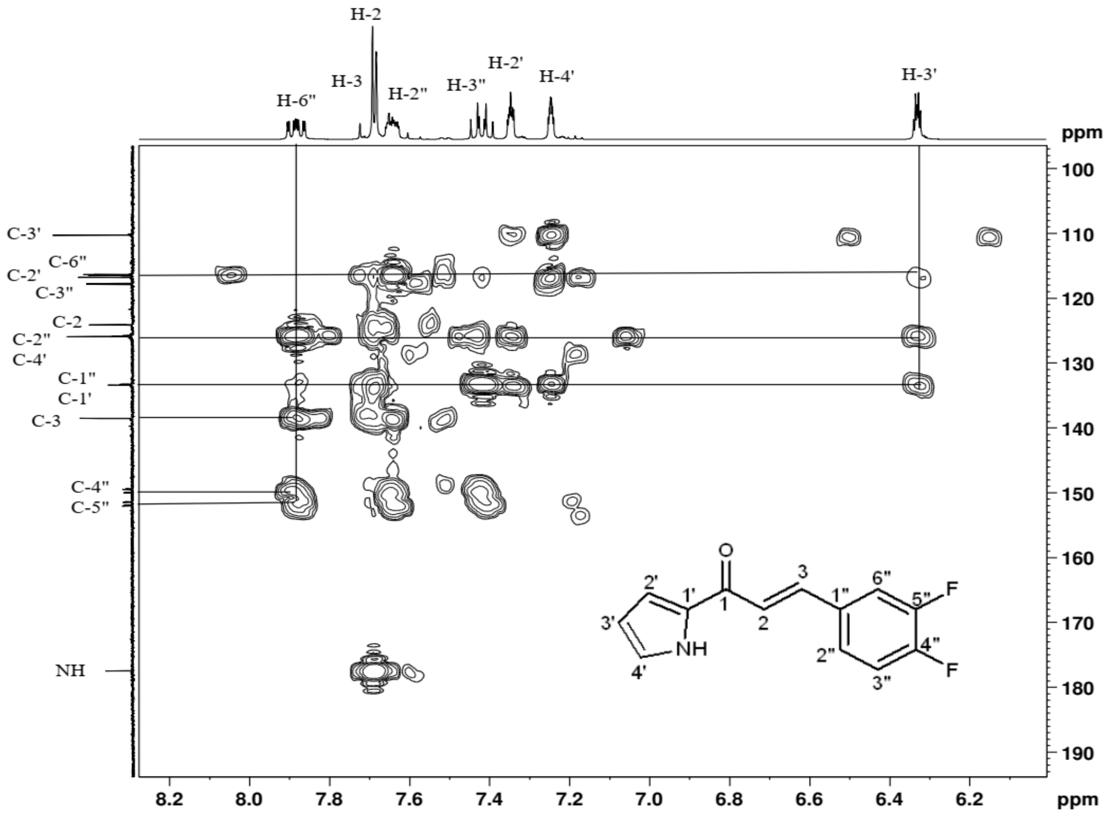

HMBC

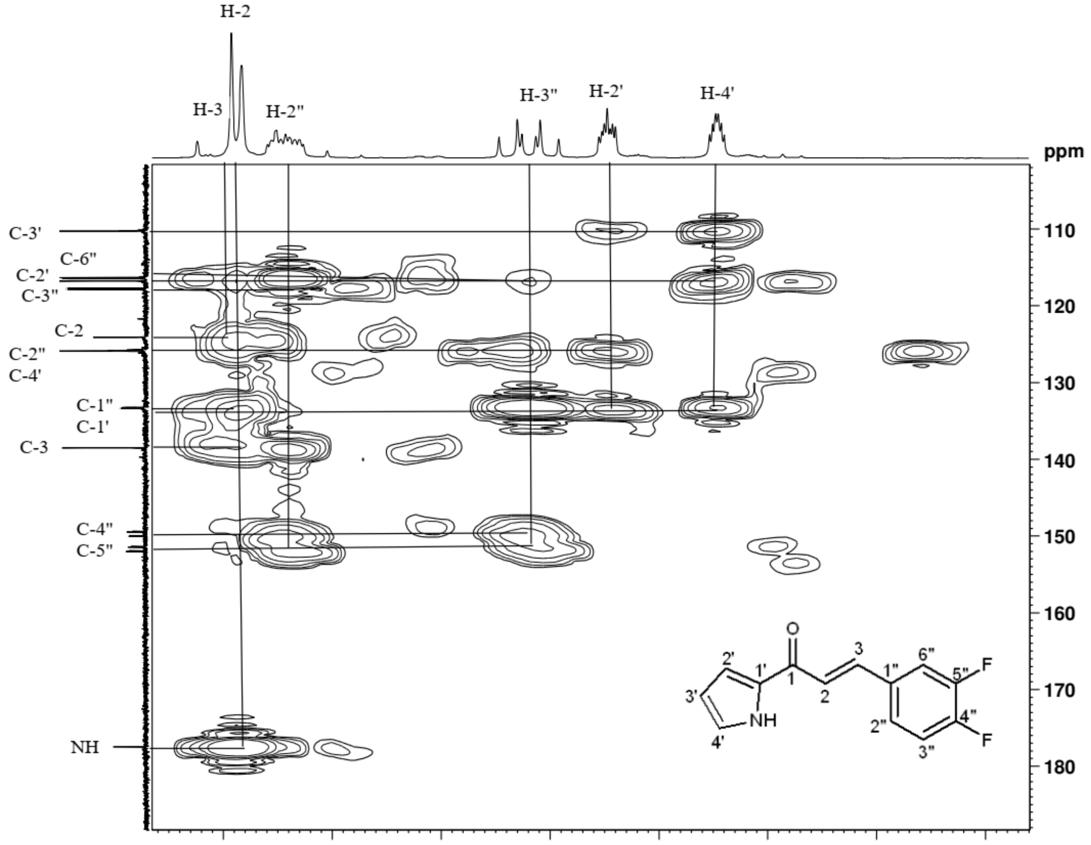

HMBC (expansion)

*3-(3, 5-Difluorophenyl)-1-(1H-pyrrol-2-yl) prop-2-en-1-one (**15**)*

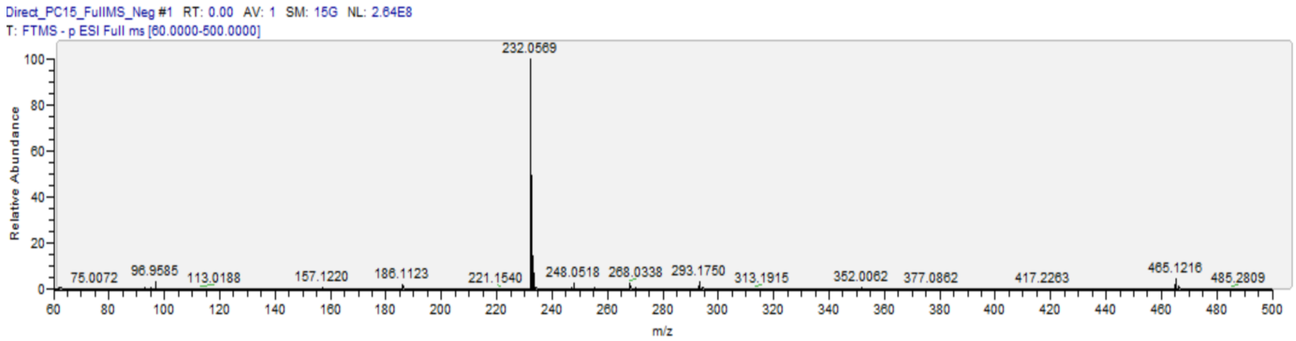

HRMS (ESI) [M-H]⁻ : found: 232.0569

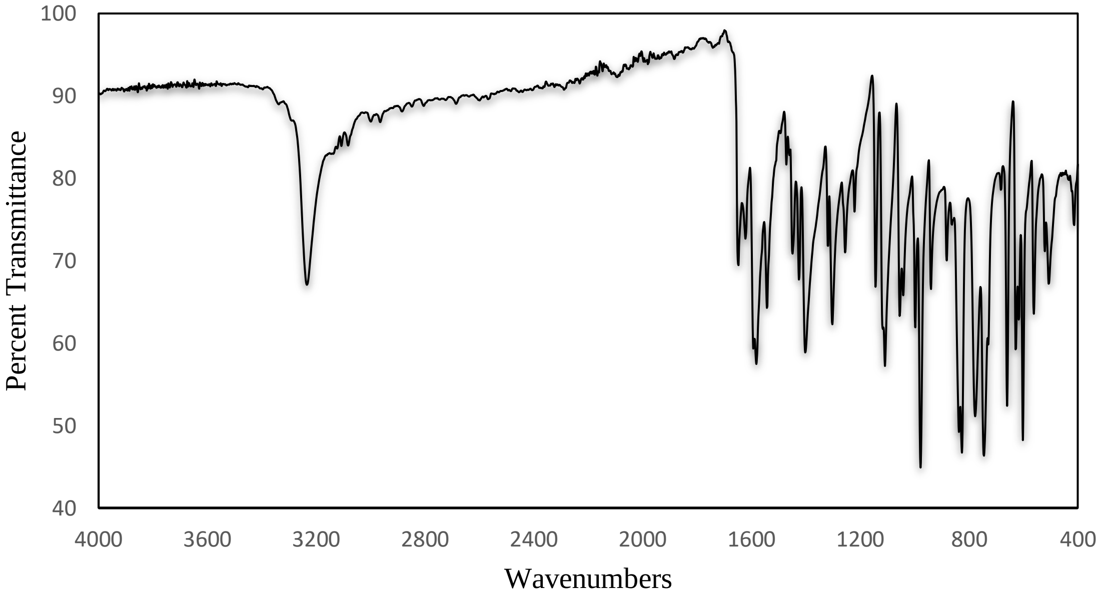

FT-IR

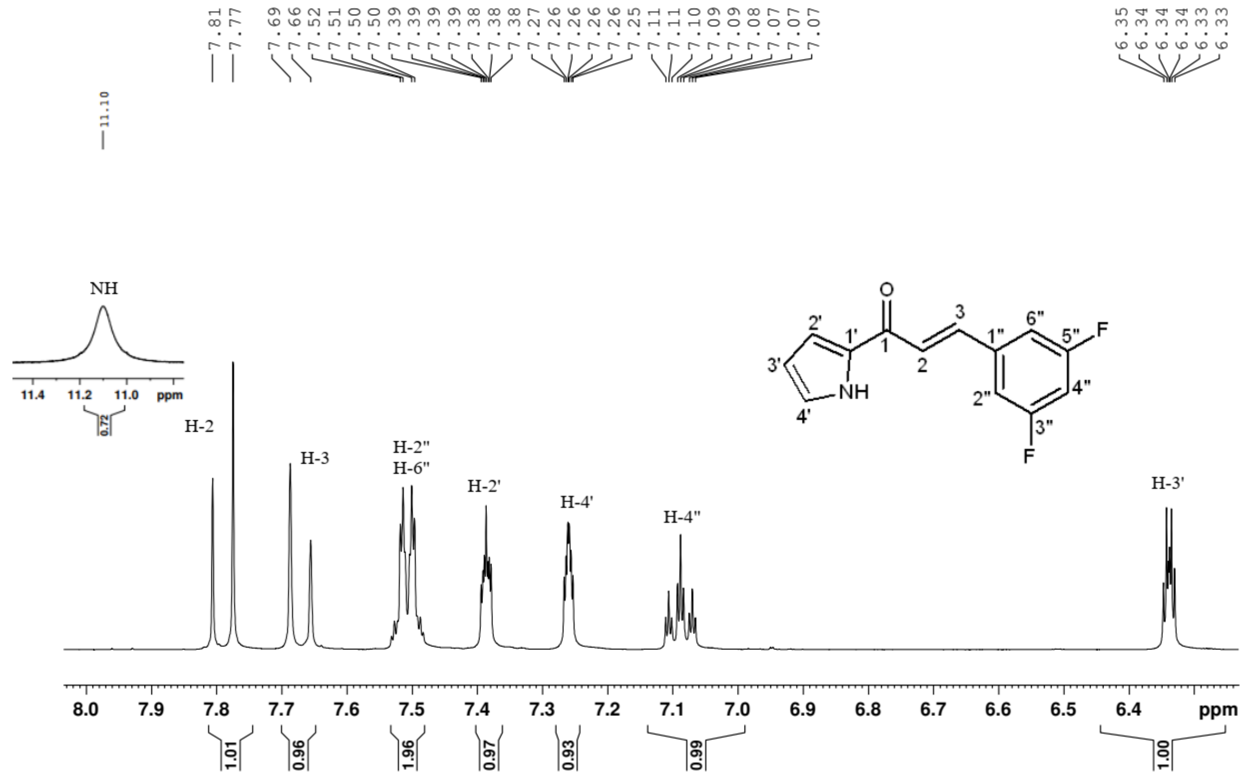

^{1}H NMR (500 MHz, acetone-d_6) δ: 6.34 (*m*, 1H, Py-H), 7.09 (*tt*, J=2.3 & 9.1 Hz 1H, Ar-H), 7.26 (*m*, 1H, Py-H), 7.39 (*m*, 1H, Py-H), 7.50 (*br. d*, *J*=2.1, 1H, Ar-H), 7.52 (*br d*, *J*=2.1, 1H, Ar-H), 7.67 (*d*, *J*=15.7, 1H, CH=CH), 7.79 (*d*, *J*=15.7, 1H, CH=CH), 11.10 (*br. s*, 1H, NH)

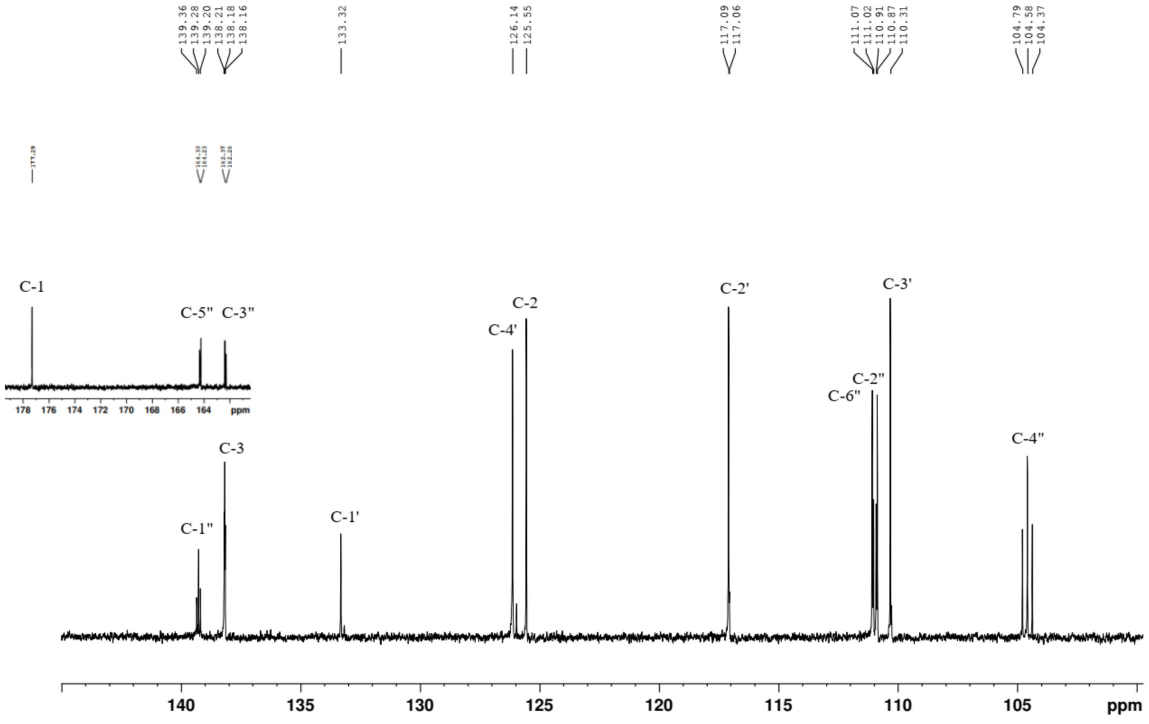

^{13}C NMR (125 MHz, acetone-d_6) δ: 104.5, 110.3, 110.9, 111.1, 117.0, 125.5, 126.1, 133.3, 138.1, 139.3, 162.3, 164.3, 177.3

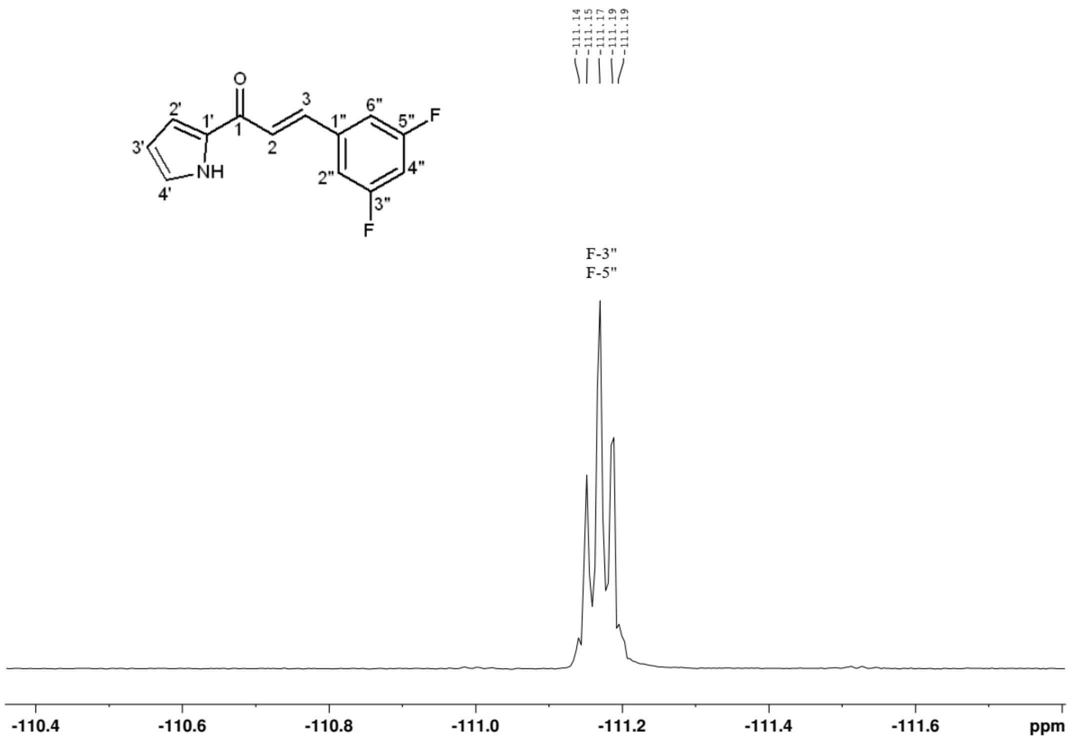

¹⁹F NMR (470 MHz, acetone-d_6) δ: -111.1

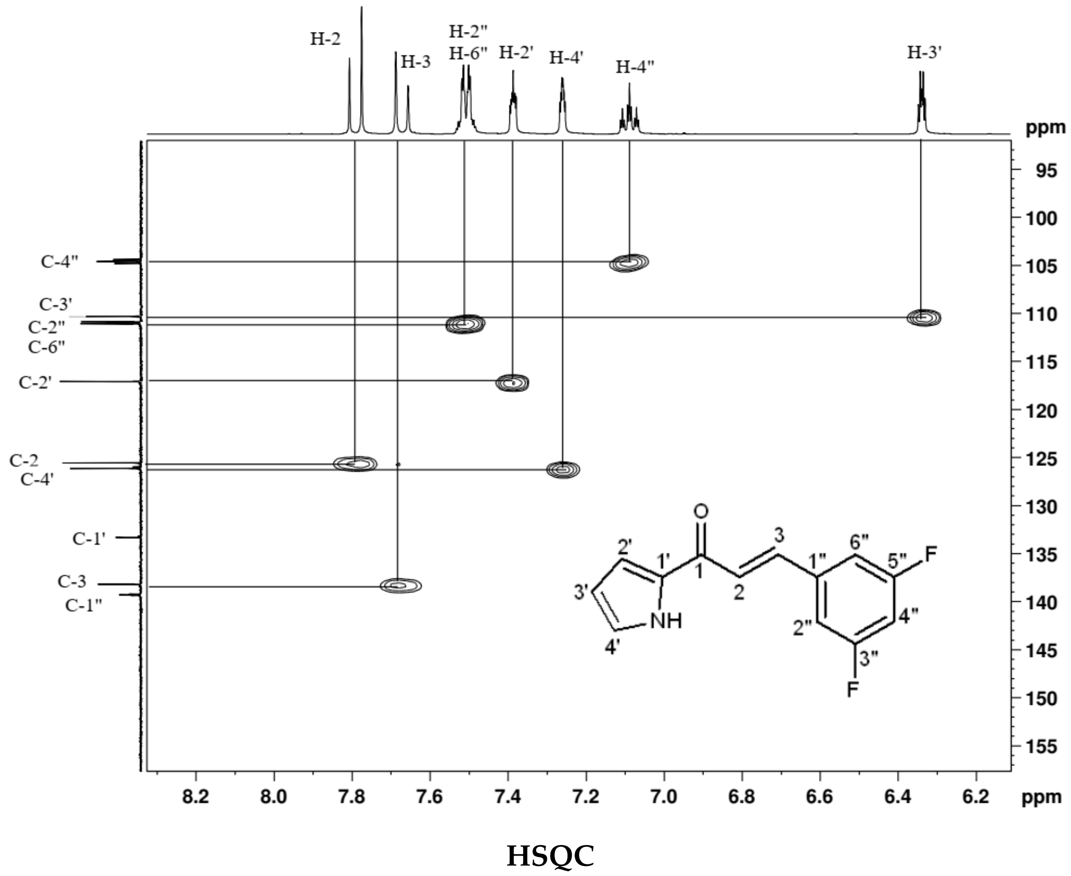

HSQC

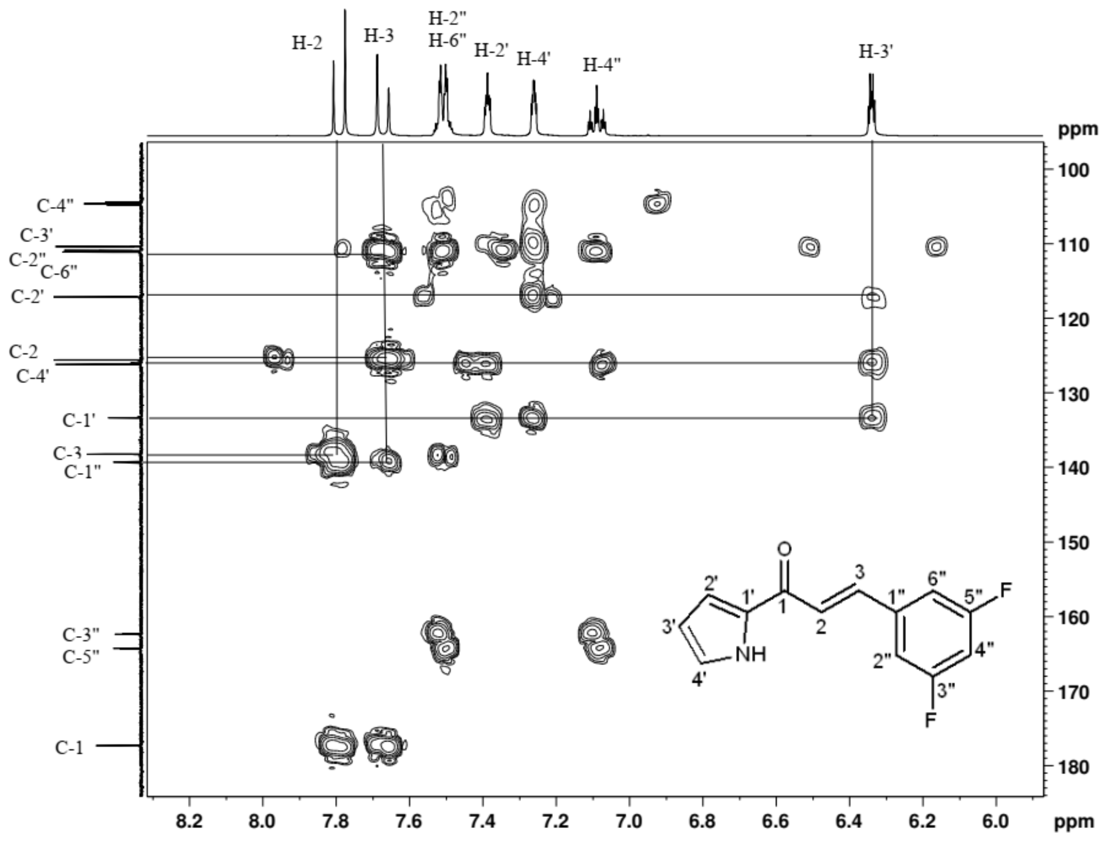

HMBC

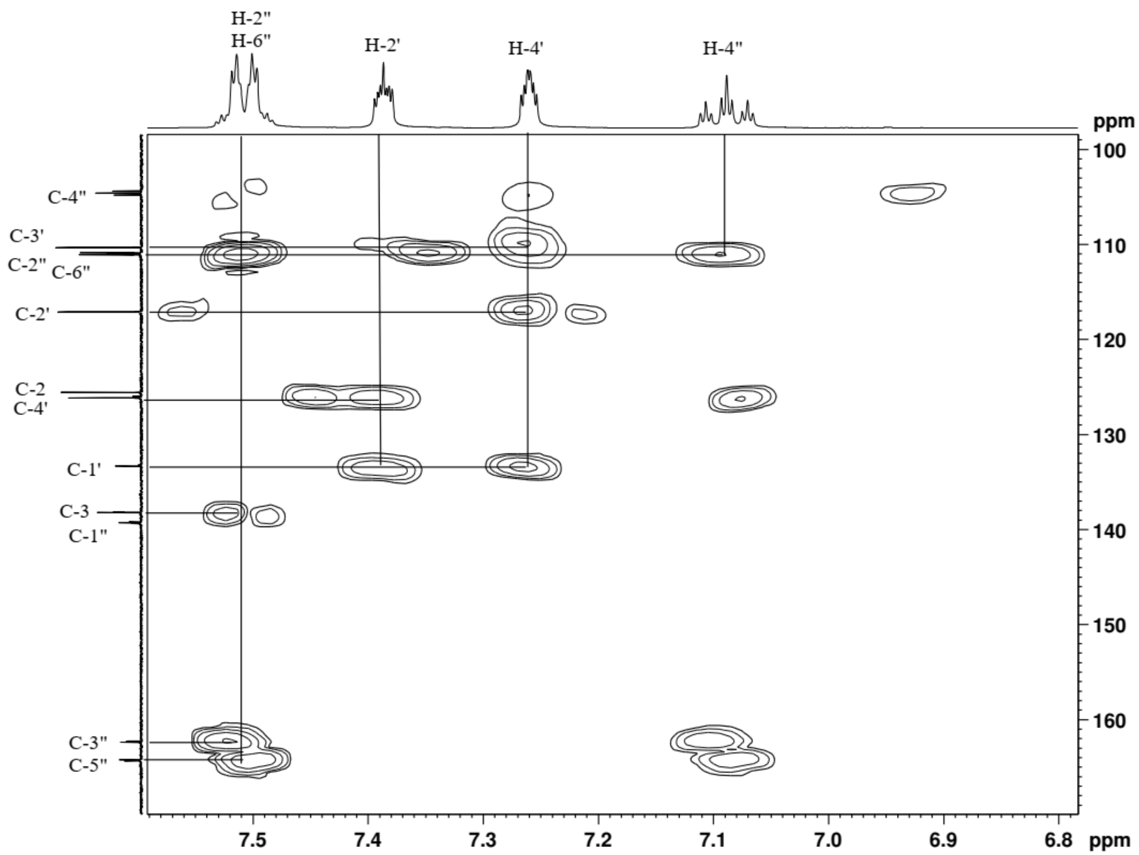

HMBC (expansion)

Table S1. Spectrometric and general data of pyrrolylated-chalcone analogues (**1-15**)

	**Compound**	**^{1}H NMR, ^{13}C NMR,^{19}F NMR**	**Mass found, [M]$^{+}$ (g/mol)**	**Yield (%)**	**Colour**	**MP (˚C)**
**1**	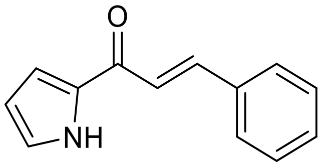	**^{1}H NMR** (700 MHz, acetone-d_6) δ: 6.32 (*m*, 1H, Py-H), 7.23 (*m*, 1H, Py-H), 7.32 (*m*, 1H, Py-H), 7.47-7.43 (*m*, 3H, Ar-H), 7.68 (*d*, *J*=15.4 Hz, 1H, CH=CH), 7.74 (*d*, *J*=16.1 Hz, 1H, CH=CH), 7.80 (*m*, 2H, Ar-H), 11.06 (*br. s*, 1H, NH)<br><br>**^{13}C NMR** (175 MHz, acetone-d_6) δ : 110.1, 116.3, 122.7, 125.5, 128.3,128.8, 129.9, 133.4, 135.3, 140.8, 177.8	197.05	13.28	Brown	133-134
**2**	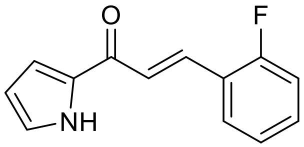	**^{1}H NMR** (500 MHz, acetone-d_6) δ : 6.33 (*m*, 1H, Py-H), 7.27 (*m* , 4H, Py-H and Ar-H), 7.50 (*m*, 1H, Ar-H), 7.73 (*d*, *J*=15 Hz, 1H, CH=CH), 7.90 (*d*, *J*=15 Hz, 1H, CH=CH), 7.98 (*td*, 1H, Ar-H), 11.11 (*br. s*, 1H, NH)<br><br>**^{13}C NMR** (125 MHz, acetone-d_6) δ : 110.3, 115.9, 116.6, 123.1, 124.7, 124.9, 125.9, 128.8, 131.8, 132.5, 133.3, 161.3, 177.5<br><br>**^{19}F NMR** (470 MHz, acetone-d_6) δ : -117.5	215.05	95.41	Pale brown	140-141
**3**	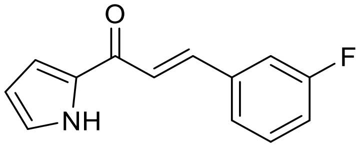	**^{1}H NMR** (500 MHz, acetone-d_6) δ : 6.34 (*m*, 1H, Py-H), 7.20 (*td*, 1H, Ar-H), 7.25 (*m*, 1H, Py-H), 7.36 (*m*, 1H, Py-H), 7.50 (*m*, 1H, Ar-H), 7.61 (*d*, 1H, Ar-H), 7.64 (*m*, 1H, Ar-H), 7.71 (*d*, *J*=15 Hz, 1H, CH=CH), 7.75 (*d*, *J*=15 Hz, 1H, CH=CH), 11.08 (*br. s*, 1H, NH)<br><br>**^{13}C NMR** (125 MHz, acetone-d_6) δ : 110.2, 114.0, 116.4, 116.7, 124.2, 124.7, 125.8, 130.7, 133.3, 137.9, 139.4, 163.1, 177.5<br><br>**^{19}F NMR** (470 MHz, acetone-d_6) δ: -114.5;	215.05	18.00	Pale yellow	138-139
**4**		**^{1}H NMR** (500 MHz, chloroform-d) δ: 6.36 (*m*, 1H, Py-H), 7.07 (*m*, 1H, Py-H), 7.10 (*br. d*, *J*=9.0 Hz, 2H, Ar-H), 7.12 (*m*, 1H, Py-H), 7.28	215.10	2.29	Pale yellow	137-38

	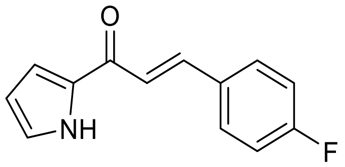	(*d*, *J*=15 Hz, 1H, CH=CH), 7.63 (*dd*, J=14.5 Hz, 2H, Ar-H), 7.79 (*d*, *J*=15 Hz, 1H, CH=CH), 11.11 (*br. s*, 1H, NH)<br><br>^{13}**C NMR** (125 MHz, chloroform-d) δ :111.0, 116.1, 116.2, 121.6, 125.2, 130.1, 131.2, 133.1, 141.0, 163.9, 178.6<br><br>^{19}**F NMR** (470 MHz, acetone-d_6) δ: -109.6				
**5**	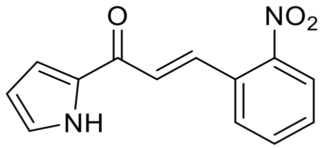	^{1}**H NMR** (700 MHz, DMSO-d_6) δ: 6.37 (*m*, 1H, Py-H), 7.37 (1H, Py-H), 7.42 (*td*, *J*=14.0 Hz, 1H, Ar-H), 7.64 (*s*, 1H, Py-H), 7.77 (*td*, *J*=14.0 Hz, 1H, Ar-H), 8.00 (*d*, *J*=7.0 Hz, 1H, Ar-H), 8.63 (*d*, *J*=8.4 Hz, 1H, Ar-H), 10.0 (*s*, 1H, NH), 12.33 (*br. s*, 2H, CH=CH)<br><br>^{13}**C NMR** (175 MHz, DMSO-d_6) δ: 112.2, 120.0, 123.7, 124.9, 128.6, 130.2, 136.2, 136.6, 138.6, 161.2, 173.5, 196.5	242.0	2.90	Grey	193-194
**6**	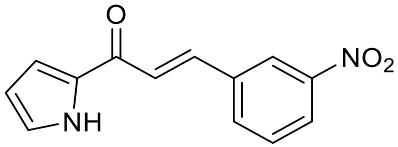	^{1}**H NMR** (700 MHz, DMSO-d_6) δ: 6.30 (*m*, 1H, Py-H), 7.21 (*br. s*, 1H, Py-H), 7.45 (*br. s*, 1H, Py-H), 7.73 (*d*, *J*=15.4 Hz, 1H, CH=CH), 7.73 (*br. dd*, *J*=14 Hz, 1H, Ar-H), 7.88 (*d*, *J*=16 Hz, 1H, CH=CH), 8.24 (*d*, *J*=10.5 Hz, 1H, Ar-H), 8.27 (*d*, *J*=7.7 Hz, 1H, Ar-H), 8.67 (*br. s*, 1H, Ar-H), 12.00 (*br. s*, 1H, NH)<br><br>^{13}**C NMR** (175 MHz, DMSO-d_6) δ : 110.9, 118.7, 123.1, 124.7, 126.1, 127.6, 130.8, 133.3, 135.1, 137.1, 138.8, 148.8, 177.8	242.0	21.71	Yellow	174-175
**7**	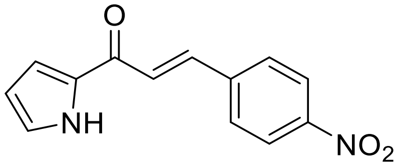	^{1}**H NMR** (700 MHz, DMSO-d_6) δ: 6.30 (*m*, 1H, Py-H), 7.21 (1H, Py-H), 7.3 (1H, Py-H), 7.72 (*d*, *J*=15.4 Hz, 1H, CH=CH), 7.87 (*d*, *J*=15.4 Hz, 1H, CH=CH), 8.10 (*d*, *J*=8.4 Hz, 2H, Ar-H), 8.26 (*d*, *J*=9.1 Hz, 2H, Ar-H), 12.04 (*br. s*, 1H, NH)<br><br>^{13}**C NMR** (175 MHz, DMSO-d_6) δ : 110.9, 118.7, 124.4, 127.5, 127.7, 129.9, 133.3, 138.5, 141.8, 148.2, 177.6	242.0	48.70	Yellow	184-185

**8**	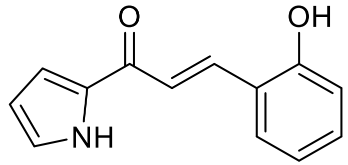	**^{1}H NMR** (700 MHz, acetone-d_6) δ : 6.17 (*m*, 1H, Py-H), 6.77 (*t*, *J*=14.0, 1H, Ar-H), 6.85 (*d*, *J*=8.4, 1H, Ar-H), 7.06 (*m*, 1H, Py-H), 7.08 (*m*, 1H, Py-H), 7.11 (*t*, *J*=16.8, 1H, Ar-H), 7.54 (*d*, *J*=15.4, 1H, CH=CH), 7.64 (*d*, *J*=9.8, 1H, Ar-H), 7.99 (*d*, *J*=15.4, 1H, CH=CH), 8.96 (1H, OH), 10.87 (*br. s*, 1H, NH)<br><br>**^{13}C NMR** (175 MHz, acetone-d_6) δ : 110.0, 115.7, 116.1, 119.9, 122.2, 125.1, 128.5, 131.1, 133.6, 136.4, 156.7, 178.4	213.05	10.83	Yellowish browm	151-152
**9**	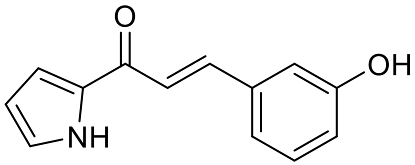	**^{1}H NMR** (700 MHz, acetone-d_6) δ : 6.33 (*m*, 1H, Py-H), 6.93 (*td*, *J*=10.0, 1H, Ar-H), 7.23 (*m*, 1H, Py-H), 7.24 (*m*, 1H, Ar-H), 7.27 (*m*, 1H, Ar-H), 7.29 (*s*, 1H, Ar-H), 7.31 (*m*, 1H, Py-H), 7.60 (*d*, *J*=15.0 Hz, 1H, CH=CH), 7.68 (*d*, *J*=15.0 Hz, 1H, CH=CH), 8.66 (1H, OH), 11.08 (*br. s*, 1H, NH)<br><br>**^{13}C NMR** (175 MHz, acetone-d_6) δ :110.1, 114.7, 116.3, 117.1, 119.7, 122.6, 125.5, 129.8, 133.4, 136.7, 141.1, 157.8, 177.9	213.05	5.09	Yellow	150-151
**10**	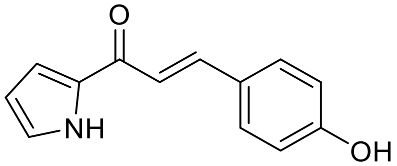	**^{1}H NMR** (700 MHz, acetone-d_6) δ : 6.30 (*m*, 1H, Py-H), 6.92 (*dd*, 2H, Ar-H), 7.20 (*m*, 1H, Py-H), 7.26 (*m*, 1H, Py-H), 7.50 (*d*, *J*=15.4, 1H, CH=CH), 7.68 (*d*, 2H, Ar-H), 7.69 (*d*, *J*=15.6, CH=CH), 8.95 (1H, OH), 11.00(*br. s*, 1H, NH)<br><br>**^{13}C NMR** (175 MHz, acetone-d_6) δ: 109.9, 115.6, 115.7, 119.5, 125.0, 126.9, 130.2, 133.5, 141.1, 159.5, 178.	213.0	12.85	Yellow	151-152
**11**	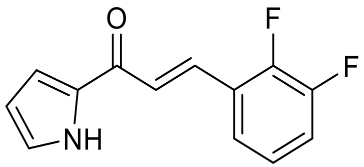	**^{1}H NMR** (500 MHz, acetone-d_6) δ : 6.34 (*m*, 1H, Py-H), 7.27 (*m*, 1H, Py-H), 7.30 (*m*, 1H, Ar-H), 7.32 (*m*, 1H, Py-H), 7.40 (*m*, 1H, Ar-H), 7.75 (*d*, *J*=16.0, 1H, CH=CH), 7.78 (*dd*, *J*=9.0, 1H, Ar-H), 7.85 (*d*, *J*=16.0, 1H, CH=CH), 11.15 (*br. s*, 1H, NH)<br><br>**^{13}C NMR** (125 MHz, acetone-d_6) δ : 110.3, 116.9, 118.2, 123.8, 124.8, 125.4, 126.2, 131.2, 133.2, 148.9, 150.7, 177.2<br><br>**^{19}F NMR** (470 MHz, acetone-d_6) δ: -140.2 & -143.6	233.0	65.37	Yellow	142-143

**12**	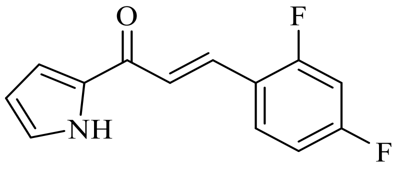	**^{1}H NMR** (500 MHz, acetone-d_6) δ : 6.33 (*br. s*, 1H, Py-H), 7.15 (*m*, 2H, Ar-H), 7.29 (*br. s*, 2H, Py-H), 7.70 (*d*, *J*=16.0, 1H, CH=CH), 7.80 (*d*, *J*=16.0, 1H, CH=CH), 8.06 (*m*, 1H, Ar-H), 11.12 (*br. s*, 1H, NH)<br><br>**^{13}C NMR** (125 MHz, acetone-d_6) δ : 104.2, 110.3, 112.1, 116.7, 119.8, 124.6, 125.9, 130.3, 131.5, 133.2, 160.6 & 162.6, 164.8 & 162.8, 177.4<br><br>**^{19}F NMR** (470 MHz,acetone-d_6) δ: -108.3 & -112.9	233.0	90.23	Pale white	118-119
**13**	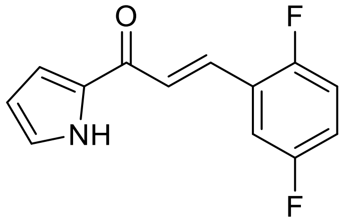	**^{1}H NMR** (500 MHz, acetone-d_6) δ : 6.34 (*m*, 1H, Py-H), 7.28 (*m*, 1H, Py-H), 7.31 (*dd*, *J*=14.0, 2H, Ar-H), 7.36 (*m*, 1H, Py-H), 7.78 (*d*, *J*=16.0, 1H, CH=CH), 7.81 (*m*, 1H, Py-H), 7.84 (*d*, *J*=16.0, 1H, CH=CH), 11.14 (*br. s*, 1H, NH)<br><br>**^{13}C NMR** (125 MHz, acetone-d_6) δ : 110.3, 114.2, 117.0, 117.5, 118.1, 124.6, 126.1, 126.2, 131.1, 133.2, 157.2, 159.2, 177.2<br><br>**^{19}F NMR** (470 MHz,acetone-d_6) δ: -119.8 & -123.4	233.0	85.28	Pale white	129-125
**14**	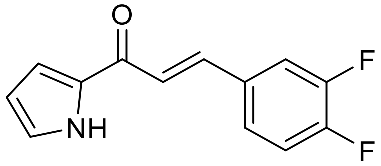	**^{1}H NMR** (500 MHz, acetone-d_6) δ: 6.33 (*m*, 1H , Py-H), 7.24 (*m*, 1H, Py-H), 7.34 (*m*, 1H, Py-H), 7.42 (*m*, 1H, Ar-H), 7.64 (*m*, 1H, Ar-H), 7.66 (*d*, *J*=16.0, 1H, CH=CH), 7.70 (*d*, *J*=16.0, 1H, CH=CH), 7.88 (*m*, 1H, Ar-H), 11.08 (*br. s*, 1H, NH)<br><br>**^{13}C NMR** (125 MHz, acetone-d_6) δ : 110.2, 116.3, 116.8, 117.7, 124.0, 125.7, 125.8, 133.2, 133.3, 138.4, 149.7, 151.7, 177.4<br><br>**^{19}F NMR** (470 MHz, acetone-d_6) δ: -137.6 & -139.5	233.0	47.51	Yellow	152-153
**15**	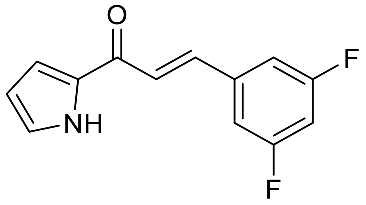	**^{1}H NMR** (500 MHz, acetone-d_6) δ: 6.34 (*m*, 1H, Py-H), 7.09 (*tt*, 1H, Ar-H), 7.26 (*m*, 1H, Py-H), 7.39 (*m*, 1H, Py-H), 7.50 (*br. d*, *J*=2.0, 1H, Ar-H), 7.52 (*br d*, *J*=2.0, 1H, Ar-H), 7.67 (*d*, *J*=15.5, 1H, CH=CH), 7.79 (*d*, *J*=15.5, 1H, CH=CH), 11.10 (*br. s*, 1H, NH)<br><br>**^{13}C NMR** (125 MHz, acetone-d_6) δ : 104.5, 110.3, 110.9, 111.1, 117.0, 125.5, 126.1, 133.3, 138.1, 139.3, 162.3, 164.3, 177.3	233.0	69.72	Yellow	151-152

**^{19}F NMR** (470 MHz,acetone-d_6) δ : -111.1

2. ***In silico* PAINS filtration, physicochemical and drug likeness properties of compounds 1-15 calculated via FAF-Drugs4.**

Filtering statistics

- **Input molecules:** 15

- **Duplicates:** 0
- **Mixtures:** 0
- **Large Compounds:** 0
- **Isotopes and Inorganics:** 0
- **Empty Structures:** 0
-

  Filtered molecules: 15
- **Rejected molecules:** 0
- **Accepted molecules:** 15
- **Intermediate molecules:** 0

- **PAINS (Pan Assays Interferences Compounds):** 0
- **Covalent Inhibitors:** 0

Table S2. The Lipinski rule values of pyrrolylated-chalcone analogues generated from FAF-Drugs4

	Structure	MW (g/mol)	Log P	HBD	HBA	RO5 violations
**1**	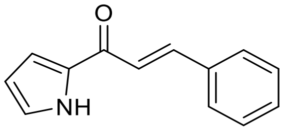	197.23	2.81	1	2	0
**2**	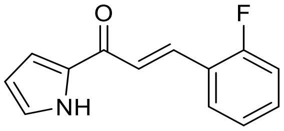	205.23	3.12	1	2	0
**3**	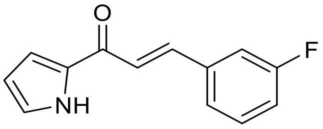	215.22	2.91	1	2	0
**4**	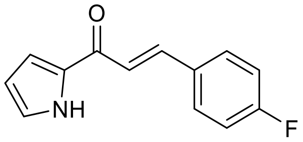	215.22	2.91	1	2	0
**5**	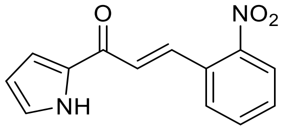	242.23	2.64	1	5	0

**6**	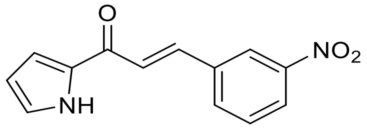	242.23	2.64	1	5	0
**7**	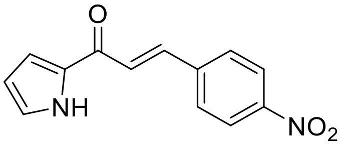	242.23	2.64	1	5	0
**8**	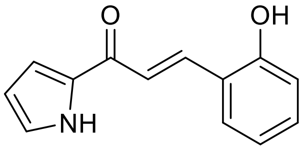	213.23	2.97	2	3	0
**9**	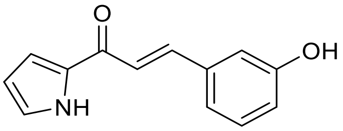	213.23	2.46	2	3	0
**10**	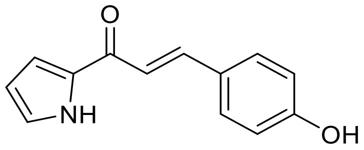	213.23	2.46	2	3	0
**11**	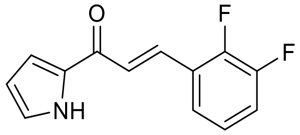	233.21	3.01	1	2	0
**12**	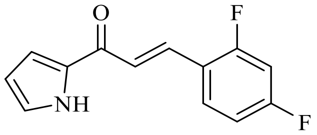	233.21	3.01	1	2	0
**13**	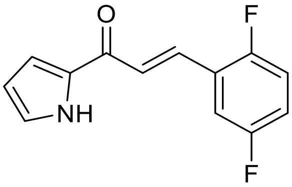	233.21	3.01	1	2	0
**14**	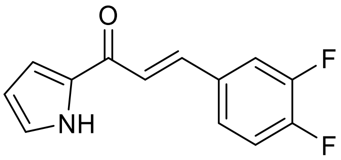	233.21	3.01	1	2	0
**15**	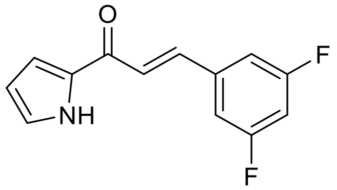	233.21	3.01	1	2	0
